# *Wolbachia* infection dynamics in a natural population of the pear psyllid *Cacopsylla pyri* (Hemiptera: Psylloidea) across its seasonal generations

**DOI:** 10.1038/s41598-022-20968-0

**Published:** 2022-10-03

**Authors:** Liliya Štarhová Serbina, Domagoj Gajski, Igor Malenovský, Erika Corretto, Hannes Schuler, Jessica Dittmer

**Affiliations:** 1 Faculty of Science and Technology, Free University of Bozen-Bolzano, Bolzano, Italy; 2 Department of Botany and Zoology, Faculty of Science, Masaryk University, Brno, Czech Republic; 3 Competence Centre for Plant Health, Free University of Bozen-Bolzano, Bolzano, Italy; 4 Present Address: Institut Agro, INRAE, IRHS, SFR Quasav, University of Angers, Angers, France

**Keywords:** Microbiology, Microbial communities, Environmental microbiology

## Abstract

*Wolbachia* is one of the most abundant intracellular symbionts of arthropods and has profound effects on host biology. *Wolbachia* transmission and host phenotypes often depend on its density within the host, which can be affected by multiple biotic and abiotic factors. However, very few studies measured *Wolbachia* density in natural host populations. Here, we describe *Wolbachia* in the pear psyllid *Cacopsylla pyri* from three populations in the Czech Republic. Using phylogenetic analyses based on *wsp* and multilocus sequence typing genes, we demonstrate that *C. pyri* harbours three new *Wolbachia* strains from supergroup B. A fourth *Wolbachia* strain from supergroup A was also detected in parasitised immatures of *C. pyri*, but likely came from a hymenopteran parasitoid. To obtain insights into natural *Wolbachia* infection dynamics, we quantified *Wolbachia* in psyllid individuals from the locality with the highest prevalence across an entire year, spanning several seasonal generations of the host. All tested females were infected and *Wolbachia* density remained stable across the entire period, suggesting a highly efficient vertical transmission and little influence from the environment and different host generations. In contrast, we observed a tendency towards reduced *Wolbachia* density in males which may suggest sex-related differences in *Wolbachia*-psyllid interactions.

## Introduction

*Wolbachia* is one of the most abundant intracellular symbionts of nematodes, insects and other arthropods^1,2^. It is particularly well-represented among terrestrial arthropod species, with 40–60% of all species estimated to be infected with *Wolbachia*^1,3^. *Wolbachia* has strong impacts on host biology, ecology and evolution, including manipulation of host reproduction via male-killing, feminization, parthenogenesis or cytoplasmatic incompatibility (CI) to promote its transmission^4–8^. On the other hand, there is strong evidence that *Wolbachia* acts as a nutritional mutualist in certain species, providing its host with vitamins and nutrients that are important for host survival^9–13^. Sixteen genetically distinct evolutionary lineages of *Wolbachia* (supergroups) have been found so far, which differ from each other in host range and biology^14–17^. The majority of the known *Wolbachia* strains fall into two major supergroups A and B, mostly consisting of reproductive parasites.

*Wolbachia* is mainly vertically transmitted from mother to offspring but can also be transmitted horizontally to new host lineages and infect populations of both related and unrelated host taxa^16–22^. Moreover, *Wolbachia* is used to limit the transmission of numerous human pathogens and agricultural pests^13,23–28^, which makes *Wolbachia* a promising tool for controlling vector-borne diseases^29–31^. Thus, several *Wolbachia* strains showed their efficiency in inhibiting mosquito-borne diseases when introduced into natural mosquito populations^23,32–34^. Likewise, several studies on agricultural pests suggested that *Wolbachia* affects the transmission of plant pathogens^24,26,27,35–38^.

While *Wolbachia*’s influence on their hosts was intensively investigated in the last two decades, e.g.^4,5,8,39,40^, factors that might affect its density dynamics were less often considered. Maintaining an optimal density is crucial for *Wolbachia* to ensure long-term and stable relationships with its host^8,41^. Hence, a reduced infection density may impede the vertical transmission of *Wolbachia*, while an excessive bacterial density could be detrimental for the host^42^. Moreover, many *Wolbachia*-induced phenotypes (reproductive manipulation, protection against pathogens) depend on sufficient *Wolbachia* density^43^. For instance, the most frequently observed *Wolbachia*-induced reproductive phenotype (CI) results in embryonic death when *Wolbachia*-infected males reproduce with uninfected females^4,8^. Hence, sufficient *Wolbachia* density in males is required to induce CI, while females are under selective pressure to maintain a sufficient *Wolbachia* concentration to protect themselves against CI. Nonetheless, it has also been observed that *Wolbachia* can be suppressed in males, potentially to reduce CI-related mortality when mating with uninfected females^4–8^. Environmental conditions (e.g. temperature, humidity), host factors (e.g. host longevity, ageing, genetic background) and interactions with other microorganisms can also have considerable effects on *Wolbachia* titer^41,44–48^. The importance of host genotype and *Wolbachia* strain for the regulation of *Wolbachia* density was demonstrated in fruit flies, parasitoid wasps, beetles, mosquitoes and isopods^49–54^. Competition for resources and space as well as interactions with other microorganisms, including viruses, bacteria and different *Wolbachia* strains, can also lead to changes in *Wolbachia* titer^55–57^. *Wolbachia* density can also fluctuate during the lifespan of a host, with varying outcomes depending on the host species. While the density declined with age in flies and *Aedes* mosquitoes^58–60^, it increased in aged *Culex pipiens* mosquitoes^61^. Similarly, a recent study on the flies *Drosophila simulans* and *D. melanogaster* demonstrated that their respective *Wolbachia* strains varied differently with host age: while *Wolbachia* density decreased in ageing males of *D. simulans*, its density in *D. melanogaster* increased^62^. These differences might be linked to differences in host immune response to *Wolbachia* infection^62^. In addition, the host mating pattern affected *Wolbachia* density dynamics in mites^63^.

The effect of temperature is especially important for *Wolbachia* density^41,45,51,64,65^. Depending on the *Wolbachia*-host association, high temperatures eliminated *Wolbachia* from their hosts^64,66–68^, increased the *Wolbachia* titer^69^or reduced the efficiency of *Wolbachia* transmission in in vitro experiments^70^. Thermal sensitivity of *Wolbachia* abundance in the fly *Drosophila melanogaster* on a global scale was recently demonstrated^71^. In contrast to experiments under controlled laboratory conditions, environmental factors such as temperature are much more variable in the wild, making it difficult to translate patterns from laboratory experiments to natural populations. Very few studies investigated the seasonal variations of *Wolbachia* infection in natural host populations^25,72–75^. Of these, only one study^73^ measured *Wolbachia* abundance using quantitative PCR (qPCR), and demonstrated a decrease of *Wolbachia* density across several generations of the pale grass blue butterfly, *Pseudozizeeria maha*, from early summer to autumn in Japan. The other studies on *Wolbachia* dynamics in natural host populations^25,72–75^ made their conclusions based on the relative abundance of *Wolbachia* in metabarcoding data. As a result, we are currently lacking insights into the dynamics of *Wolbachia* infections in natural host populations, particularly for non-model organisms.

*Wolbachia* was detected in many psyllid species (Insecta: Hemiptera: Psylloidea)^76–80^ but it was thoroughly studied only in pest species, such as the citrus psyllid *Diaphorina citri* and the potato psyllid *Bactericera cockerelli.* Particularly, the studies on *D. citri* suggest that *Wolbachia* could mitigate and even impede the transmission of the pathogen ‘*Candidatus* Liberibacter asiaticus’^35,37,81,82^. In addition, several *Wolbachia* strains from *B. cockerelli* are able to induce CI^83–86^. The recent metabarcoding study by^87^ revealed high relative abundance of *Wolbachia* in several populations of the pear psyllid *Cacopsylla pyri* (Psyllidae) from the Czech Republic. In one of these populations, *Wolbachia* was detected across different developmental stages and seasonal generations throughout an entire year.

In the present study, we aim to investigate *Wolbachia* infection and density dynamics throughout an entire year in natural populations of *C. pyri*. This psyllid is an economically important pest that transmits ‘*Candidatus* Phytoplasma pyri’, the causative agent of the Pear Decline disease of pear trees in Europe^88^. In contrast to many other psyllid species from northern temperate latitudes, *C. pyri* is a non-migrating species that usually spends its entire life cycle on pear trees and therefore it is present in pear orchards all year long, producing several summer generations and one overwintering generation^89,90^. In Central Europe, *C. pyri* starts reproducing in the middle or at the end of spring, giving rise to the first summer generation after overwintering on its host-plant^89^. This makes *C. pyri* a suitable model system to characterise *Wolbachia* seasonal dynamics in a natural environment. In the current study, we (i) characterise *Wolbachia* in *C. pyri* from three localities from the Czech Republic, (ii) analyse the phylogenetic relationships between the different *Wolbachia* strains and (iii) investigate the density dynamics of the most prevalent *Wolbachia* strain in one population throughout an entire year using qPCR. To our knowledge, this is the first study demonstrating *Wolbachia* density dynamics in a natural host population across an entire year using a targeted qPCR approach. Our results provide new insights into the impact of seasonal dynamics on *Wolbachia* density in a natural population of psyllids and will broaden our general understanding of *Wolbachia*-host interactions in natural environments.

## Results

### *Wolbachia* prevalence

**Figure 1:**
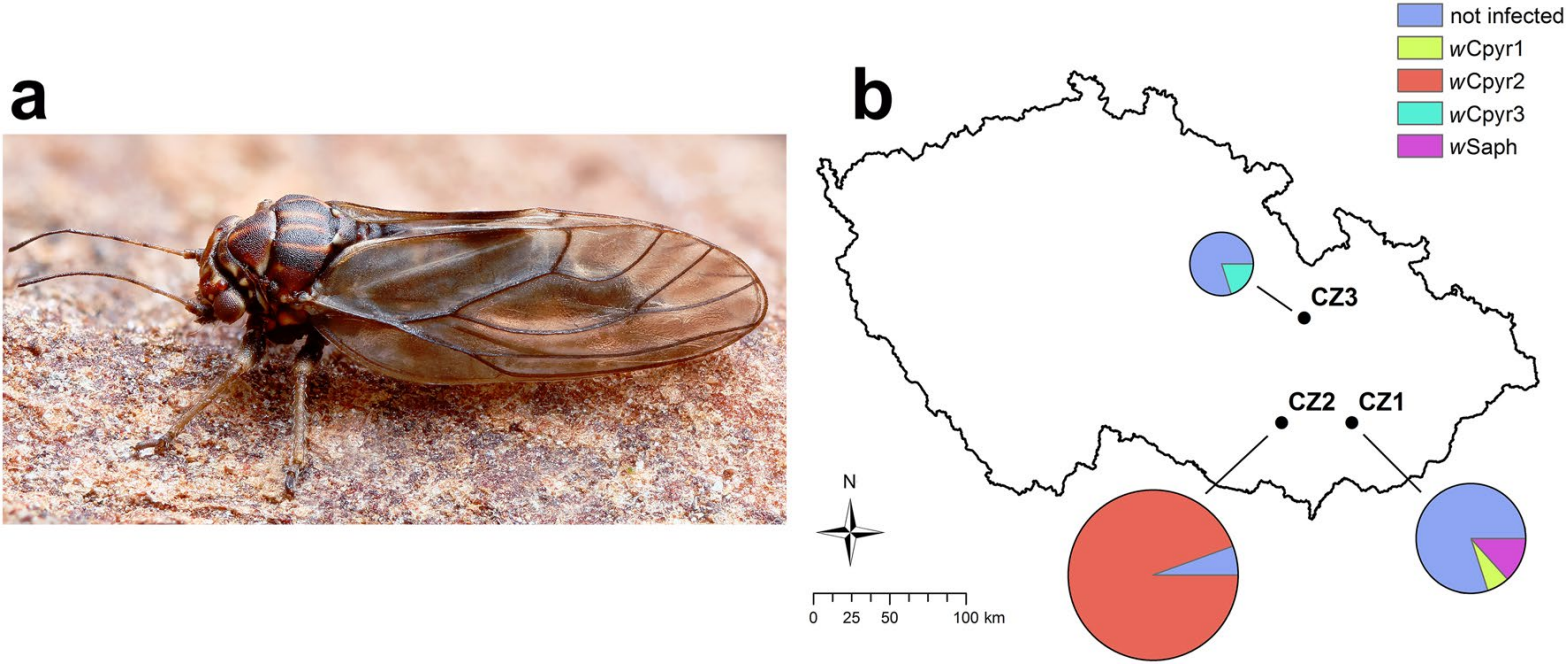
**(a)** Adult pear psyllid *Cacopsylla pyri* (photograph by O. Michálek). **(b)** *Wolbachia* prevalence in each of three studied populations of *C. pyri* in the Czech Republic. Pie charts represent the percentage of *C. pyri* individuals infected with different *Wolbachia* strains (*w*Cpyr1, *w*Cpyr2, *w*Cpyr3 and *w*Saph) or not infected with *Wolbachia*. Sample size per population is indicated by the size of the pie chart. Details of localities and sample size are given in Table 1. The map was produced in ArcGIS Desktop v10.8.2 (ESRI) (https://desktop.arcgis.com).

From the 56 analysed individuals of *C. pyri* (Fig. 1a), 38 were tested positive for *Wolbachia* (Table 1). The highest *Wolbachia* prevalence was observed in the population CZ2 (94.4%; N = 34/36), while in CZ1 (N = 1/10) and CZ3 (N = 1/5) the *Wolbachia* prevalence reached only 10% and 20%, respectively (Fig. 1b). Moreover, the two out of five (40%) parasitised immatures from CZ1 were infected with *Wolbachia*.

**Table 1:** Prevalence of the identified *Wolbachia* strains in three populations of *C. pyri.*

Host species: *Wolbachia* strain	Total N	Infected N	Date	Locality
*C. pyri*: *w*Cpyr1	5 ♂, 5 ♀	1 ♂	04.12.2019	CZ1: Litenčice, CZ
*C. pyri*: *w*Cpyr2	12 ♂, 22 ♀, 2 immatures	10 ♂, 22 ♀, 2 immatures	15.02.2020 – 14.02.2021	CZ2: Starý Lískovec, Brno, CZ
*C. pyri*: *w*Cpyr3	5 immatures	1 immature	07.07.2020	CZ3: Staré Město, Svitavy, CZ
Parasitoid of *C. pyri* (identified as *Syrphophagus aphidivorus*): *w*Saph	5 immatures	2 immatures	04.12.2019	CZ1: Litenčice, CZ

Information on developmental stage, sex, number of tested specimens (Total N), number of specimens infected with *Wolbachia* (Infected N), collection date and locality are provided. CZ = Czech Republic. This information is also visualized in Fig. 1b.

### Identification of *Wolbachia* strains and supergroups in *Cacopsylla pyri*

Sanger sequencing of *wsp* and MLST genes was performed for all 38 *Wolbachia*-infected individuals, except for the *ftsZ* gene of individuals from CZ3 which failed to amplify. Sequence analysis indicated the presence of three novel alleles for *fbpA*, two novel alleles for *wsp* and *ftsZ*, and one for *coxA*, *gatB* and *hcpA* (Table 2). Together, these alleles represented four new *Wolbachia* sequence types (Table 2, Supplementary Table S1). Chromatograms showed clear single peaks, suggesting the presence of just one *Wolbachia* strain in each individual, which allowed to assign the different alleles to three different strains. They were named *w*Cpyr1, *w*Cpyr2 and *w*Cpyr3. None of the strains were previously described in other organisms.

**Table 2:** *Wsp* and MLST gene allelic profiles.

Host species: *Wolbachia* strain	*wsp*	*coxA*	*fbpA*	*ftsZ*	*gatB*	*hcpA*	ST
*C. pyri*: *w*Cpyr1	*(3)	131	*(3)	*(3)	9	227	*
*C. pyri*: *w*Cpyr2	*(3)	131	*(2)	*(3)	9	227	*
*C. pyri*: *w*Cpyr3	521	173	187	–	39	*(1)	*
Parasitoid of *C. pyri* (identified as *Syrphophagus aphidivorus*): *w*Saph	*(4)	*(3)	*(6)	*(1)	*(2)	60	*

Novel alleles and sequence types (ST) are indicated with an asterisk. Numbers of single nucleotide differences to the closest allele match in the MLST database are provided in parentheses. Identical new alleles in *w*Cpyr1 and *w*Cpyr2 are highlighted in bold.

**Figure 2:**
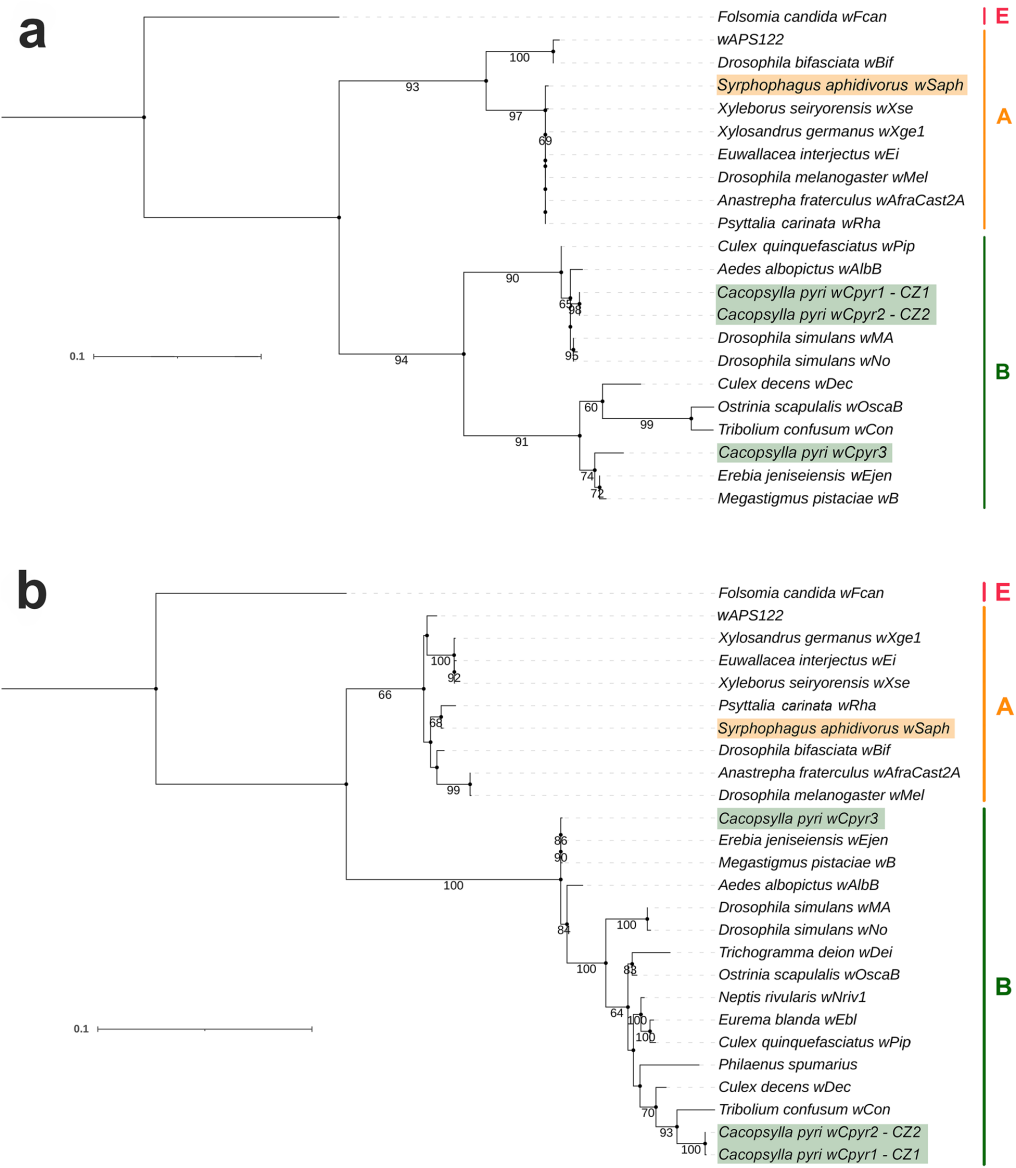
Maximum likelihood tree of *Wolbachia* **(a)** *wsp* gene sequences, **(b)** concatenated MLST gene sequences. Bootstrap values > 60% are shown. The new strains *w*Saph, *w*Cpyr1, *w*Cpyr2 and *w*Cpyr3 are highlighted. *Wolbachia* supergroups are indicated by the coloured bar on the right-hand side. GenBank accession numbers for the strains used for the phylogenetic analyses are provided in the Supplementary Table S3.

Both ML and BI phylogenetic analyses based on the *wsp* and MLST genes showed that all three *Wolbachia* strains found in *C. pyri* belong to supergroup B (Fig. 2, Supplementary Fig. S1). The strains *w*Cpyr1 and *w*Cpyr2 were differentiated by a single nucleotide in the *fbpA* gene (*p*-distance = 0.003), whereas the strain *w*Cpyr3 was more divergent from both *w*Cpyr1 and *w*Cpyr2 for three of the five analysed genes (*wsp*: *p*-distance = 0.144; *coxA*: *p*-distance = 0.148; *fbpA*: *p*-distance = 0.065 and 0.064, respectively; *gatB*: *p*-distance = 0.003; *hcpA*: *p*-distance = 0.002) (Supplementary Table S1). MLST alleles *gatB* and *hcpA* of *w*Cpyr1 and *w*Cpyr2 showed 100% identity with the strain *w*Nriv1 from the butterfly *Neptis rivularis* (Table 2, Supplementary Table S1). Similarly, BLAST identified identical sequences to *gatB* and *hcpA* of *w*Cpyr1 and *w*Cpyr2 in several other strains. Among them, *gatB* sequences were identical to *w*Dec from the mosquito *Culex decens* and *w*OscaB from the butterfly *Ostrinia scapulalis*; *hcpA* sequences were identical to *w*Con from the beetle *Tribolium confusum* and an unnamed strain from the froghopper *Philaenus spumarius*. In contrast, *wsp*, *fbpA* and *ftsZ* of *w*Cpyr1 and *w*Cpyr2 represented new alleles based on comparisons with both the MLST database and BLAST. In spite of the aforementioned similarities in two MLST genes, the *wsp* sequences of *w*Dec, *w*OscaB and *w*Con were quite different from the *wsp* of *w*Cpyr1 and *w*Cpyr2 with a genetic divergence of 0.167, 0.209 and 0.194, respectively. Based on the comparisons of *p*-distances, *gatB* demonstrated the least sequence divergence, ranging from 0.001 to 0.010. These differences between the *wsp* and the MLST genes were also reflected in the phylogenetic trees: according to the *wsp* tree, *w*Cpyr1 and *w*Cpyr2 were most closely related to two strains from *Drosophila simulans* (*w*No, *w*Ma) and two strains from the mosquitoes *Aedes albopictus* (*w*AlbB) and *Culex quinquefasciatus* (*w*Pip) (Fig. 2a, Supplementary Fig. S1a).

Based on comparisons with alleles from the MLST database, four genes (*wsp*, *coxA*, *fbpA* and *gatB*) of *w*Cpyr3 were identical to those of the strain *w*Ejen from the butterfly *Erebia jeniseiensis* (Table 2, Supplementary Table S1). Additionally, BLAST searches showed that *gatB* of *w*Cpyr3 was identical to several other *Wolbachia* strains, including *w*Ebl from the butterfly *Eurema blanda* and *w*B from the parasitoid wasp *Megastigmus pistaciae*, and *fbpA* of *w*Cpyr3 was identical to *w*AlbB from the mosquito *Aedes albopictus*. In contrast to *wsp*, *coxA*, *fbpA* and *gatB*, we found no sequences identical to the *hcpA* allele of *w*Cpyr3. Similar to the results on the allele comparison, the ML and BI *wsp* trees strongly supported the clade with *w*Cpyr3 and the two closely-related strains *w*B and *w*Ejen within supergroup B (BS = 91; BI = 1) (Fig. 2a, Supplementary Fig. S1a).

Additional ML and BI phylogenetic analyses were performed including also the *wsp* sequences of previously identified *Wolbachia* strains from other psyllid species. The results demonstrated that the *Wolbachia* strains from *C. pyri* belonged to different clades from the strains of other psyllid species, including those from other *Cacopsylla* species (Supplementary Fig. S2). Nevertheless, the *Wolbachia* strains from all analysed psyllid species belong to supergroup B.

### Identification of a *Wolbachia* strain of potentially parasitoid origin

Two of the five immature individuals of *C. pyri* from CZ1 were infected with another *Wolbachia* strain that was placed within supergroup A (*wsp*: BS = 93, BI = 1; MLST: BS = 66, BI = 1) (Figs. 2a–b, Supplementary Figs. S2a–b, Table 1). These immatures had been shown to be parasitised according to a COI barcoding approach^87^. Although the identification of the parasitoid present in the immatures was not formal, the detected *Wolbachia* strain was named *w*Saph after its potential host, *S. aphidivorus*. Based on comparisons with the MLST database, the *wsp* and MLST alleles (except *hcpA*) of *w*Saph were different from previously sequenced strains present in this database (Table 2, Supplementary Table S1). Additionally, BLAST searches revealed that the *hcpA* gene of *w*Saph was identical to several other strains, including *w*Bif from the fly *Drosophila bifasciata* and *w*Rha from the parasitoid wasp *Psyttalia carinata* (Braconidae), whereas *fbpA* of *w*Saph was identical to *w*Bif.

### Density dynamics of wCpyr2 throughout an entire year

**Figure 3:**
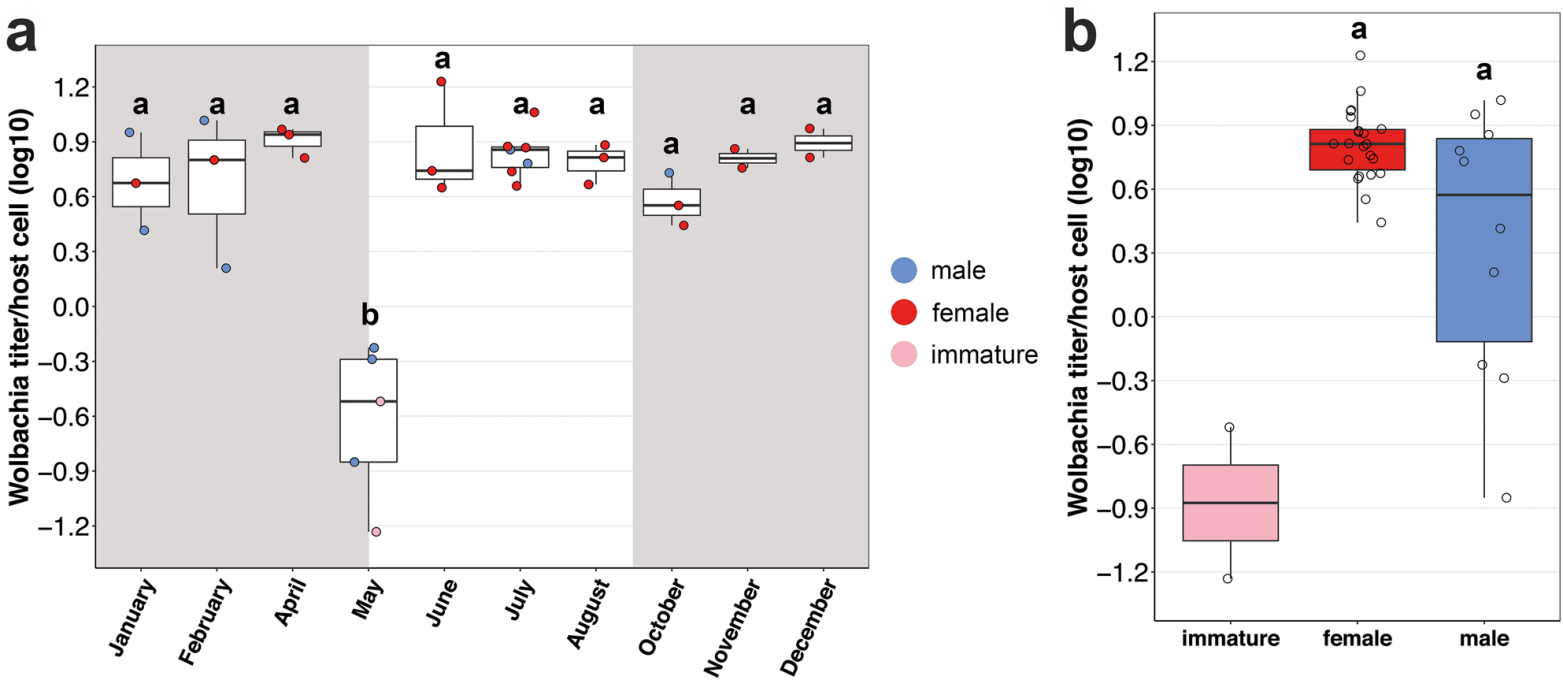
Seasonal dynamics of *Wolbachia* (*w*Cpyr2) density in *Cacopsylla pyri* individuals **(a)** throughout the sampling year, **(b)** between immatures, females and males. The shaded areas correspond to the period of occurrence of the overwintering generation of *C. pyri*, the clear area represent the period of occurrence of its summer generations. For more details on the analysed individuals of *C. pyri,* see Supplementary Table S2.

The *C. pyri* population CZ2 had the highest *Wolbachia* prevalence among the studied populations (94.4%; Fig. 1b) and therefore was chosen for monitoring of the *Wolbachia* density dynamics. To investigate whether seasonal changes of local abiotic (Supplementary Fig. S3) and biotic factors affected the density of *Wolbachia* in *C. pyri*, we compared the densities of *w*Cpyr2 from psyllid individuals sampled from CZ2 throughout the year. Among all quantified psyllid specimens (N = 36), only two males (P58 and P61; Supplementary Table S2) collected in November and December, were negative. Hence, either these individuals were not infected with *w*Cpyr2 or this strain was present at low titers not detectable with our qPCR approach (Fig. 3a). Psyllid individuals sampled in November and December were excluded from the statistical analysis as only two infected individuals remained for these months after eliminating the two uninfected males from the dataset.

Monitoring of *w*Cpyr2 titers throughout the remaining eight months revealed that *Wolbachia* titer/host cell in all individuals ranged between 0.06 and 16.91 (Supplementary Table S2). The individuals collected in May showed a significantly lower titer (mean *Wolbachia* titer/host cell: 0.42) compared to individuals sampled in all other months (mean *Wolbachia* titer/host cell: 1.62 in February—16.91 in June) (Tukey’s post-hoc test: *p* ≤ 0.0002). When considering also the sex of the tested individuals, our data showed that the *Wolbachia* titer in females was relatively stable throughout the year, while it was more variable in males (Figs. 3a–b). In May, only males were captured and those had the lowest *Wolbachia* titers detected during the entire study period, thereby driving this drop in *Wolbachia* titer in May. Despite a tendency towards lower *Wolbachia* titers in males, a global comparison of the *Wolbachia* titers between males and females across all time points was not significant (Welsh’s *t*-test: *t* = 2.2067, df = 9.6116, *p* = 0.0529) (Fig. 3b). This outcome may have been influenced by the low number of males (N = 12) collected only in five out of ten sampling months (Supplementary Table S2). In addition, two immatures collected in May also had low *Wolbachia* titers (0.06 and 0.30 *Wolbachia*/host cell) (Fig. 3b), but these individuals were not included in the statistical analysis due to their different developmental stage.

## Discussion

*Wolbachia* density plays an important role in the evolution of symbiotic associations and ecological interactions, mainly through its impact on symbiont transmission and host extended phenotype^8,41,50,52,66,71^. Although different environmental factors have a strong influence on *Wolbachia* titre^64,66–68,70,91^, the majority of studies focusing on *Wolbachia* density dynamics were performed under controlled laboratory conditions. Given that host-microbe associations in the wild are likely influenced by multiple environmental factors (e.g. temperature, humidity), host factors (e.g. host longevity, ageing, genetic background) and interactions with other organisms, controlled laboratory environments cannot encompass the full spectrum of these factors and their potential impacts^41,44–48,92^. In the current study, we present one of the few examples of the dynamics of *Wolbachia* in a natural host population across different generations and seasons.

We assessed the *Wolbachia* density dynamics in a natural population of the pear psyllid *C. pyri*, across several generations throughout an entire year. *Cacopsylla pyri* completes its life cycle on pear trees and therefore represents an interesting model to study *Wolbachia* density dynamics through different seasons. This psyllid species produces several summer generations and one overwintering generation on pear trees^89,90^, which allows studying the impact of varying environmental conditions on the *Wolbachia* density throughout the year*.* Only a few studies attempted to describe *Wolbachia* seasonal dynamics in natural host populations so far^72–74^. In the butterfly *Pseudozizeeria maha*, the *Wolbachia* density decreased across several host generations from spring/early summer to autumn^73^. Likewise, the relative abundance of *Wolbachia* within the microbiome declined from spring to summer in the flea *Synosternus cleopatrae*^74^. A decrease in *Wolbachia* relative abundance, through its vertical transmission to the subsequent host generation, was correlated with higher temperatures in natural populations of the mosquito complexes *Aedes vexans* and *Culex pipiens/restuans*^25^. However, as none of these studies included individuals sampled in winter, the effect of harsh winter conditions on *Wolbachia* dynamics in overwintering hosts has not been analysed yet.

To investigate *Wolbachia* density dynamics in a natural population of *C. pyri* across all seasons, we measured the titer of the most frequently occurring strain (*w*Cpyr2) at a single locality throughout an entire year. Although the bacterial density in natural populations can be influenced by multiple factors, *Wolbachia* density was stable in the analysed females of *C. pyri*, which were abundantly infected with *Wolbachia* throughout the sampling period, suggesting a highly efficient vertical transmission of *w*Cpyr2 from mothers to daughters across different host generations. Regardless of their fluctuations (e.g. a significant decrease of local temperature during the winter, Fig. S3), the environmental conditions had no impact on *Wolbachia* density in females. This might indicate a strong selective pressure on females to maintain a sufficient *Wolbachia* density in order to protect themselves against cytoplasmatic incompatibility (CI)^4^. As such, several *Wolbachia* strains from supergroup B that are closely related to *w*Cpyr2 are known to induce CI, i.e. two strains from the fly *Drosophila simulans* (*w*Ma, *w*No) and two strains from the mosquitoes *Aedes albopictus* (*w*AlbB) and *Culex quinquefasciatus* (*w*Pip)^93–95^. Taken together, this suggests that *w*Cpyr2 might also be able to induce CI but this needs to be validated experimentally.

In contrast to females, not all tested males harboured *Wolbachia* and its titer in males was more variable compared to the females. Considering the efficient *Wolbachia* transmission from mothers to daughters, it is unlikely that male embryos received less *Wolbachia* from their mothers. Instead, *Wolbachia* could multiply less in males or could be suppressed in males in order to reduce CI-related mortality when mating with an uninfected female. This would result in reduced *Wolbachia* titers in aged males. Such a pattern was predicted by population genetic models and observed in the mosquito *Aedes albopictus* and the fly *Drosophila innubila*^58,59,96^. In accordance with these predictions, male individuals in our study showed a general tendency towards reduced *Wolbachia* density compared to females, culminating in a significant drop in *Wolbachia* titer in May. However, since no females were sampled in May, we cannot exclude that the titer might have been low also in females at this time point. In Central Europe, a new annual cycle of *C. pyri* begins in April–May, giving rise to the first summer generation after overwintering and newly-emerged adults from this generation can be already found in the second half of May^89^. At this time, aged adults from the previous overwintering generation can still be alive and present in the orchard as well. In contrast to *D. innubila* and *A. albopictus* mosquitoes, the *Wolbachia* titer of mosquitoes from the *Culex pipiens* group increased with ageing and therefore young males of *C. pipiens* had a lower *Wolbachia* titer compared to older individuals^61^. Consequently, the males of *C. pyri* collected in May with a low *Wolbachia* titer could have been either old individuals from the overwintering generation (if *Wolbachia* titer decreases with age) or young summer-form adults (if *Wolbachia* titer increases with age). Both scenarios would agree with those from the previous studies that demonstrated the impact of age on *Wolbachia* titer in laboratory studies of different systems^58–62^. Moreover, the number of males collected throughout the study period was half the number of females. While this could be a coincidence, it could also be the result of another *Wolbachia*-induced reproductive manipulation, e.g. male-killing or feminization^4–8^. However, the presence of *Wolbachia* in males, albeit at more variable titer, makes this scenario less likely.

In the current study, we characterised not only *w*Cpyr2 but also two additional *Wolbachia* strains (*w*Cpyr1 and *w*Cpyr3) which all belong to *Wolbachia* supergroup B. Each strain was detected as a single infection in individuals from three different localities in the Czech Republic. Hence, multiple *Wolbachia* strains occur in this host species, but they appear not to co-occur in a same individual of *C. pyri*. Currently, we cannot draw any conclusions whether the observed patterns of *Wolbachia* infection reflect stable host-symbiont associations with at least two divergent strains (e.g.^97^), an ongoing spread of one or more newly-acquired *Wolbachia* strains (e.g.^98^), or replacement of a pre-existing strain by a new, more competitive strain (e.g.^99^). To predict *Wolbachia* infection spread and potential introductions into new host populations, further studies should investigate the infection dynamics of the newly discovered *Wolbachia* strains on a larger geographical scale. In future studies, it is also necessary to sequence the whole genome of the new *Wolbachia* strains to understand how they diverged. This is particularly relevant for the strains *w*Cpyr1 and *w*Cpyr2 that differed from each other only slightly based on the MLST genes. Wolfe et al*.*^100^ demonstrated that small differences in the MLST genes can in fact reflect bigger structural differences in the whole genome of the symbiont. Additionally, it was striking to find that neither of the strains *w*Cpyr1, *w*Cpyr2 and *w*Cpyr3 showed close relatedness to the *Wolbachia* strains from other psyllid species, including other *Cacopsylla* species (e.g.^79^).

An additional *Wolbachia* strain was detected in immatures of *C. pyri* parasitised by a hymenopteran wasp that was tentatively identified as *Syrphophagus aphidivorus* based on insect DNA barcoding. *Syrphophagus* spp. are relatively frequent parasitoids or hyperparasitoids of various psyllids, including *Cacopsylla* spp. on pear trees^101^. This *Wolbachia* strain clustered within supergroup A and was closely related to *Wolbachia* strains from other hymenopteran parasitoid wasps. Despite a high importance of parasitoids for a biological control of psyllids, only limited information is available on the microbial communities of their parasitoids^102,103^. Particularly, *Wolbachia* was recently detected in the parasitoids of the citrus psyllid *Diaphorina citri*^103^, whereas to our knowledge, our study represents the first detection of *Wolbachia* in parasitised individuals of *Cacopsylla*. Considering the high incidence of parasitoids in pear psyllid populations, particularly in immatures^104,105^, horizontal transmission of *Wolbachia* between psyllids via shared parasitoids might occur*,* as it was described in other host-parasitoid associations^18,106–108^. However, a horizontal transmission from parasitoids to their hosts is quite unlikely, as parasitised hosts die and therefore are not able to maintain the bacteria throughout later life stages and pass them onto next generations^1,19,102^. Thus, it is more likely that bacterial symbionts hitchhike from hosts to parasitoids. In the case of *w*Saph, we can only speculate whether this *Wolbachia* strain originally belonged to the parasitoid or to *C. pyri*. To investigate the possibilities of horizontal transmission of *w*Saph in the wild, further experimental studies on host-parasitoid interactions in pear orchards are required.

In the current work, we studied the density dynamics of the newly-described *Wolbachia* strain *w*Cpyr2 of the pear psyllid *C. pyri* across an entire year, spanning several host generations. We demonstrated that *Wolbachia* density remained stable in females, while we observed a tendency towards reduced *Wolbachia* density in males, with a significant drop in *Wolbachia* titer in May. However, in light of a relatively low sample size of *C. pyri*, these results should be interpreted with caution. Moreover, in similar studies we highly recommend to do the non-destructive DNA extractions and retain voucher specimens in order to be able to identify the generations of the analysed individuals. Nevertheless, our study is the first demonstration of the seasonal dynamics of *Wolbachia* in psyllids and the first study to monitor *Wolbachia* abundance in a natural host population across an entire year. The identification of three different *Wolbachia* strains in *C. pyri* and another strain in parasitised individuals of *C. pyri* highlight that additional studies are needed to study phenotypic effects and spatial dynamics of *Wolbachia* in natural host populations of this important pest species. Taken together, this study provides insights into the ecological and evolutionary dynamics of *Wolbachia*-host interactions in the natural environment and opens new research questions on this study system.

## Methods

### Psyllid sampling

Adult and immature specimens of *Cacopsylla pyri* (Fig. 1a) were collected from three different localities in the Czech Republic: Litenčice = CZ1, Starý Lískovec (Brno) = CZ2, and Staré Město (Svitavy) = CZ3 (Fig. 1b, Table 1). In the locality CZ2, *C. pyri* adults were collected throughout an entire year, from February 2020 to February 2021, with a time interval of 7–10 days between each visit. In contrast, only one sampling was performed in CZ1 and CZ3. At each visit, psyllids were collected from five branches of five randomly chosen pear trees. Adult specimens of *C. pyri* were sampled using entomological sweep nets and a beating tray, while 4–5th instar immatures were collected with a camelhair brush. All individuals were immediately stored in absolute ethanol and later kept at − 20 °C. *Cacopsylla pyri* was identified based on the morphological keys by^109,110^.

Several immatures of *C. pyri* from CZ1 were shown to be parasitised based on COI gene sequences amplified from the sample DNA^87^. This barcoding approach allowed a tentative identification of the parasitoid as *Syrphophagus aphidivorus* (Hymenoptera: Encyrtidae) based on the closest BLAST hit. The immatures of *C. pyri* from CZ1 were included in the current study to explore *Wolbachia* infection in parasitised psyllid individuals*.*

### DNA extraction and strain identification

Fifty-six individuals (17 ♂, 27 ♀, 12 imm.) of *C*. *pyri* from the three aforementioned populations, CZ1 (N = 15), CZ2 (N = 36) and CZ3 (N = 5), were tested for *Wolbachia* infection (Table 1). The DNA of 43 individuals (14 ♂, 24 ♀, 5 imm.) was extracted using the DNeasy Blood and Tissue Kit (Qiagen), while the DNA of 13 specimens (3 ♂, 3 ♀, 7 imm.), included in our previous metabarcoding study^87^, had been extracted using E.Z.N.A.® Tissue DNA Kit (Omega Bio-tek).

Identification of *Wolbachia* strains and supergroups was based on the amplification of the *Wolbachia* surface protein *wsp*^111^ and the five multilocus sequence typing (MLST) genes *coxA*, *fbpA*, *ftsZ*, *gatB* and *hcpA*^112^. Due to its fast-evolving rate, *wsp* provides many informative characters that help resolving the relationships between different *Wolbachia* strains^113,114^. The MLST genes are more conserved and were broadly applied for *Wolbachia* strain detection in numerous studies (e.g^14,15,17,114–117^). Each 25 μl PCR reaction was composed of 1X DreamTaq PCR Master Mix (Thermo Fisher), 7 μl of sterile water, 0.7 μM of each primer and 2 μl of DNA template. PCR conditions were as follows: 95 °C for 5 min; 40 cycles of 95 °C for 15 s, the optimal annealing temperature (see below) for 30 s and 72 °C for 1 min; 72 °C for 10 min. The optimal PCR annealing temperature was 53 °C for *hcpA*, 54 °C for both *gatB* and *ftsZ*, 55 °C for *coxA*, and 59 °C for *fbpA*^112^. PCR conditions for *wsp* were as follows: 95 °C for 5 min; 40 cycles of 95 °C for 15 s, 58 °C for 30 s and 72 °C for 1 min; 72 °C for 10 min, using the primer pair 81F and 691R^111^. PCR products were purified and Sanger sequenced by Eurofins Genomics (Ebersberg, Germany). Each PCR product was sequenced in both directions and the consensus sequences were obtained using ‘MEGA X’^118^.

### Phylogenetic characterization of *Wolbachia* strains based on *wsp* and MLST

The *wsp* sequences were aligned with the 21 most closely related *Wolbachia* strains (Supplementary Table S3) identified from both the *Wolbachia* MLST database^119^ and GenBank based on BLAST^120^. The alignment (Supplementary File 1) was produced using the MAFFT v7 web server, applying the G-INS-I strategy^121^. Additionally, an extended *wsp* tree was produced including the *wsp* sequences from other psyllid-associated *Wolbachia* strains from GenBank as well (Supplementary File 2). The five MLST genes were concatenated (1970 bp, Supplementary File 3) and manually aligned with the same 21 *Wolbachia* strains (Supplementary Table S3) using ‘MEGA X’^118^. The phylogenetic relationships were evaluated through maximum likelihood (ML) analyses using IQ-TREE v1.6.12^122^ and Bayesian Inference (BI) analysis using MrBayes v3.2.7a^123^. As an outgroup, we used the *Wolbachia* strain of *Folsomia candida* from supergroup E (Supplementary Table S3). Nodal support for the ML analysis was calculated using a standard nonparametric bootstrap (BS) with 1000 replicates. Clades with BS > 70% were considered strongly supported and with BS 60–70% moderately supported. For the BI analysis, the best substitution model for each gene partition was estimated using Jmodeltest v2.1.10^124^ based on Akaike Information Criterion (AIC) and Bayesian Information Criterion (BIC) scores (Supplementary File 4). The analysis of 15 million generations was run on the CIPRES platform^125^. Nodal support was assessed by posterior probabilities (PP). Nodes were considered strongly supported with PP > 90%. Both ML and BI trees were visualized with iTOL v6^126^.

Consensus sequences obtained in this study were submitted to GenBank with the following accession numbers: ON146571–ON146574 and ON157498–ON157516 (Supplementary Table S3).

### Quantitative PCR and statistical analyses

In total, for the quantitative PCR we analysed 36 specimens (12 ♂, 22 ♀, 2 imm.) of *C. pyri* collected from CZ2 across an entire year from February 2020 to February 2021. At least three psyllid individuals were captured for each month of the sampling year, with the exception of March and September, when no *C. pyri* individuals were found.

All samples were run in duplicates on a CFX96 real-time PCR system (Bio-Rad, Hercules, CA, USA). *Wsp* and the host gene wingless (*wg*) were amplified using primers specific for the analysed *Wolbachia* strain *w*Cpyr2 and the *wg* gene of *C. pyri*. For *wsp*, the primer set from Le Clec'h et al*.*^127^ was modified, producing a 202 bp-amplicon: *w*Cpyr_Fq (5'-TGGTGCAGCATTTACTCCAAC-3') and *w*Cpyr_Rq (5'-TTGCTTGATAAGCAAAACC-3'). PCR conditions were as follows: 95 °C for 3 min; 40 cycles of 95 °C for 15 s, 55 °C for 30 s and 72 °C for 30 s. For *wg*, we designed new primers amplifying a 186 bp amplicon: Wg-202Fq (5'-CTCGTCTACCTGGAGACCTC-3') and Wg-362Rq (5'-ACGCAGGAAATCACTGTT-3'). PCR was performed with the following conditions: 95 °C for 3 min; 40 cycles of 95 °C for 15 s, 58 °C for 30 s and 72 °C for 30 s. Each 20 μl reaction contained 1X IQ SYBR Green Supermix (Bio-Rad), 6 μl of sterile water, 0.5 μM of each primer and 2 μl of DNA template. The amplification efficiency of the *wsp* and *wg* primer pairs was tested using a standard curve at different annealing temperatures to determine the optimal annealing temperature for the highest amplification efficiency, which was 92% for *wsp* and 95% for *wg*. To verify the amplification of the target PCR product across all reactions, a melting curve analysis was performed at the end of each run. Gene copy numbers were determined based on standard curves consisting of tenfold serial dilutions of longer PCR products of the same genes. The longer PCR products for the standard curves were amplified by standard PCR using the DreamTaq PCR Master Mix (Thermo Fisher) and the following primer sets: 81F and 691R for *wsp*^111^ and 19F 5'-ACATGYTGGATGAGAYTACCA-3' and 388R 5'-TCTTGTGTTCTATAACCACGCCCAC-3' for *wg* (this study). PCR conditions for *wg* were as follows: 95 °C for 5 min; 40 cycles of 95 °C for 15 s, 58 °C for 30 s and 72 °C for 1 min; 72 °C for 10 min. The resulting amplicons were purified using AMPure XP beads (Beckman-Coulter) and quantified using the Qubit dsDNA High Sensitivity Assay Kit (Invitrogen).

To calculate the *Wolbachia* titer/host cell ratio, the mean number of *wsp* copies was divided by the mean number of *wg* copies for each specimen. All qPCR data were log-transformed and analysed in R v3.6.3 using the packages agricolae and car^128–130^. Psyllid specimens that were not infected with *Wolbachia* as well as immatures (for which only two biological replicates were available), were eliminated from the statistical analyses. The dataset was tested for normality and homogeneity of variance using the Shapiro–Wilk and Levene tests, respectively. Welsh’s *t*-test was used to test for potential differences in *Wolbachia* titer between males and females. Analysis of variance (ANOVA) followed by Tukey’s post-hoc test for multiple comparisons was used to test for differences in *Wolbachia* titer between different months of the year. The data was visualized using the ggplot package in R^131^. The map showing the representation of different *Wolbachia* strains in each studied population of *C. pyri* (Fig. 1b) was produced in ArcGIS Desktop v10.8.2 (ESRI).

## Supplementary Information

Supplementary Information.

## Data Availability

All data generated or analysed during this study are included in the current paper and in its Supplementary Information Files. The datasets generated during the current study in GenBank with the following accession numbers, ON146571–ON146574 and ON157498–ON157516.
