# Supplementary material for: Wolbachia infection dynamics in a natural population of the pear psyllid Cacopsylla pyri (Hemiptera: Psylloidea) across its seasonal generations
Source: Sci Rep. 2022 Oct 3;12:16502. doi: 10.1038/s41598-022-20968-0 (PMC9529970; doi:10.1038/s41598-022-20968-0)
Supplement: Supplementary file 1 — Supplementary Information. [file 41598_2022_20968_MOESM1_ESM.pdf]

***Wolbachia* infection dynamics in a natural population of the pear psyllid *Cacopsylla pyri* (Hemiptera: Psylloidea) across its seasonal generations**

Liliya Štarhová Serbina, Domagoj Gajski, Igor Malenovský, Erika Corretto, Hannes Schuler and Jessica Dittmer

Figure S1. Bayesian inference tree of *Wolbachia* (a) *wsp* gene sequences, (b) concatenated MLST gene sequences. Posterior probability values > 0.9 are shown. The new strains *w*Saph, *w*Cpyr1, *w*Cpyr2 and *w*Cpyr3 are highlighted. *Wolbachia* supergroups are indicated by the coloured bar on the right-hand side. GenBank accession numbers for the strains used for the phylogenetic analyses are provided in Table S1.

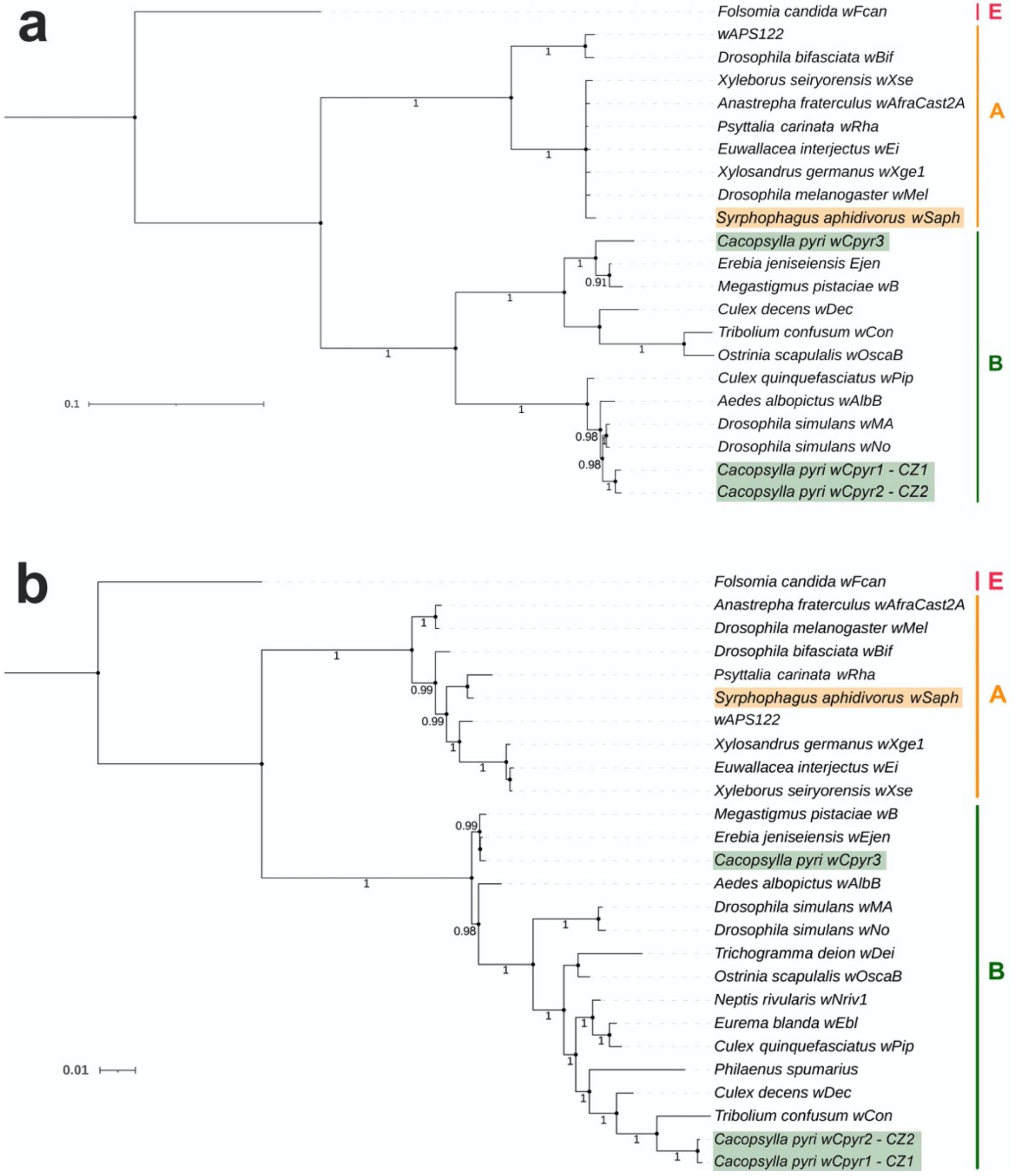

Figure S2. Maximum likelihood tree (a) and Bayesian inference tree (b) of *wsp* gene sequences of *Wolbachia*. Bootstrap values > 60% and posterior probability values > 0.9 are shown. The new strains *w*Saph, *w*Cpyr1, *w*Cpyr2 and *w*Cpyr3 are highlighted in orange and green. The strains from other psyllid species are highlighted in blue. Supergroups are indicated by the coloured bar on the right-hand side. GenBank accession numbers for the strains used for the phylogenetic analyses are provided in Table S1.

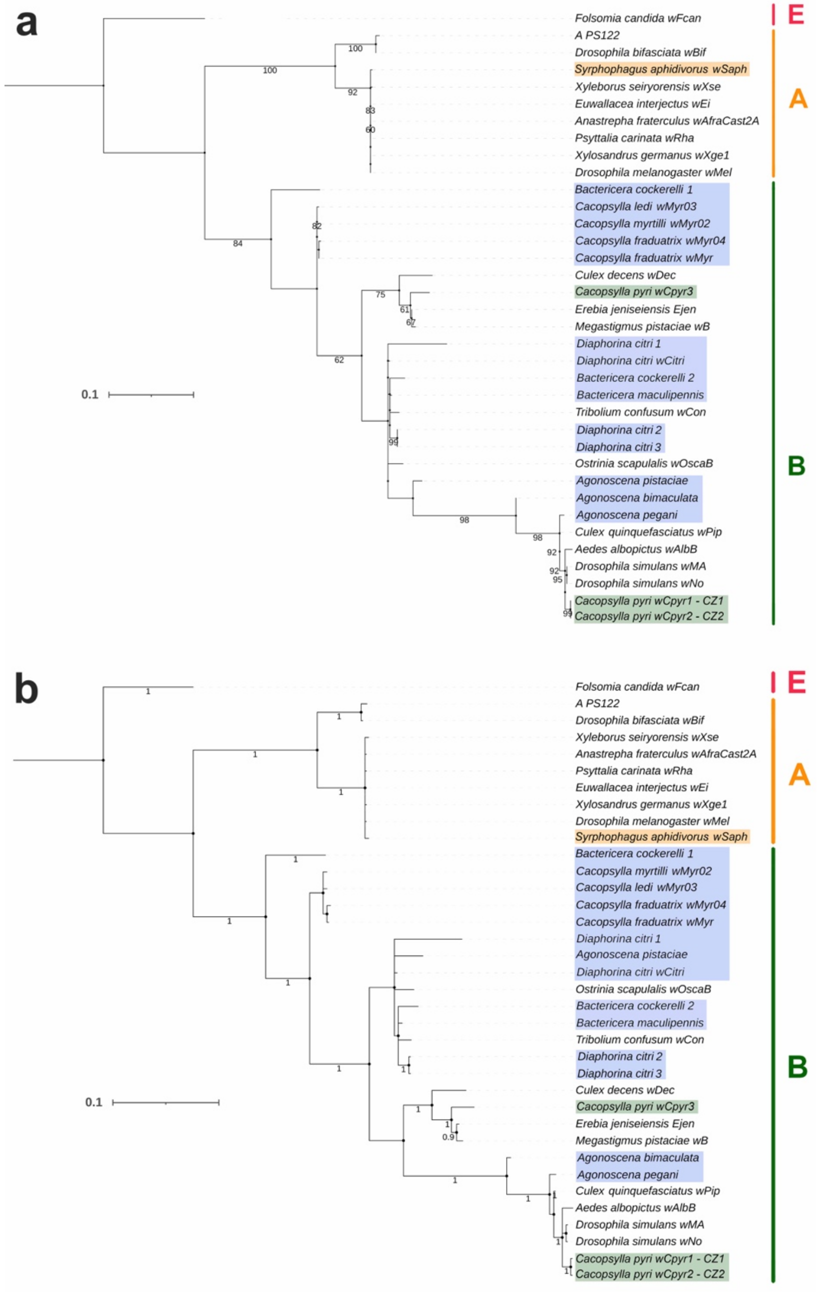

Figure S3. Monthly average temperature and monthly precipitation during the sampling period of *Cacopsylla pyri* (data from January 2020 to February 2021 measured at the weather station in Brno-Tuřany, Czech Hydrometeorological Institute, www.chmi.cz).

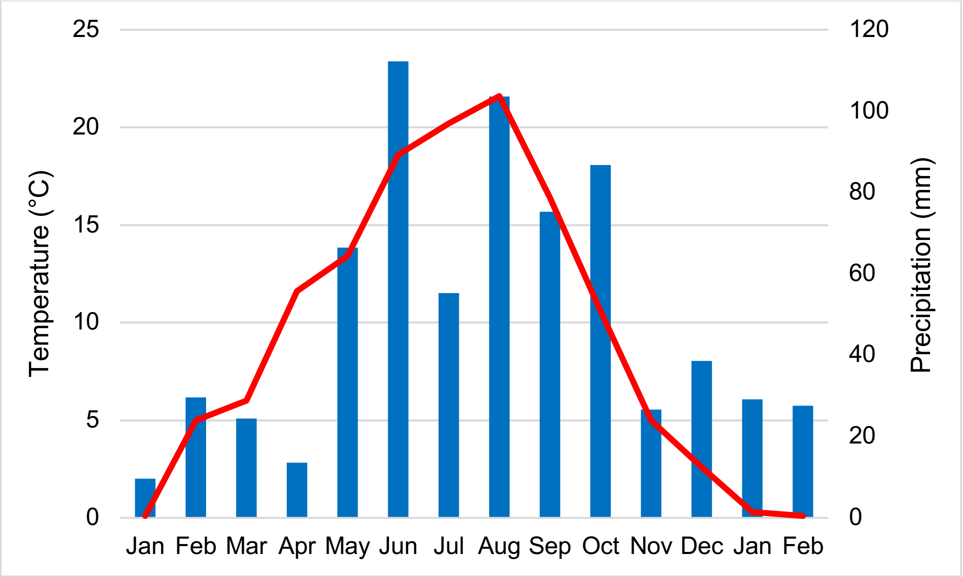

Table S1. Pairwise genetic distances for wsp and the five MLST genes between the strains *w*Saph, *w*Cpyr1, *w*Cpyr2, *w*Cpyr3 and their most closely related strains according to the comparisons from the MLST database and BLAST.

Wolbachia strain	*wsp*	*coxA*	*fbpA*	*ftsZ*	*gatB*	*hcpA*
***w*Saph**	**0**	**0**	**0**	**0**	**0**	**0**
*w*AfraCast2A	0.002	0.022	0.037	0.004	0.022	0.017
*w*Ei	0.002	0.005	0.023	0.002	0.036	0.019
*w*Mel	0.002	0.022	0.037	0.003	0.022	0.017
*w*Rha	0.002	0.003	0.028	0.053	0.005	0
*w*Xge1	0.002	0.005	0.017	0.002	0.036	0.020
*w*Xse	0.004	0.005	0.017	0.002	0.036	0.019
*w*APS122	–	0.011	0.026	0.005	0.028	0
*w*Bif	0.091	0.022	0	0.007	0.022	0
***w*Cpyr3**	**0**	**0**	**0**	**–**	**0**	**0**
*w*Ejen	0.023	0	0	–	0	0.001
*w*B	0.028	0.003	–	–	0	0.002
*w*Cpyr2 (CZ2)	0.144	0.148	0.064	–	0.003	0.002
*w*Cpyr1 (CZ1)	0.144	0.148	0.065	–	0.003	0.002
*w*AlbB	0.161	0.016	0	–	0.015	0.012
*w*Ebl	–	0.141	–	–	0	0.019
*w*Nriv1	–	0.141	0.008	–	0.003	0.001
***w*Cpyr2 (CZ2)**	**0**	**0**	**0**	**0**	**0**	**0**
***w*Cpyr1 (CZ1)**	0	0	0.003	0	0	0
*w*Ma	0.009	0.043	0.083	0.042	0.001	0.009
*w*No	0.009	0.043	0.089	0.042	0.001	0.012
*w*Pip	0.013	0.072	0.073	0.042	0.006	0.019
*w*AlbB	0.015	0.140	0.065	0.047	0.004	0.009
*w*Con	0.194	0.064	0.008	0.039	0.010	0
*w*Dei	–	0.093	0.077	0.053	0.003	0.019
*w*Dec	0.167	0.075	0.003	0.042	0	0.009
*w*Ebl	–	0.069	0.073	0.045	0.001	0.017
*w*Nriv1	–	0.069	0.073	0.037	0	0
*w*OscaB	0.209	0.069	0.067	0.045	0	0.002

unnamed strain of *Philaenus spumarius*	–	0.081	0.112	0.048	0.006	0
*w*Cpyr3	0.144	0.148	0.064	–	0.003	0.002

Table S2. The list of *Cacopsylla pyri* specimens analysed with qPCR collected in the pear orchard (CZ2 = Starý Lískovec, Brno, Czech Republic) with full information on their developmental stage, sex and collection date. Specimens P1–P8 were also included in the metabarcoding study by Štarhová Serbina et al. (2022). SQ Mean-*wsp* and SQ Mean-*wg* correspond to mean copy numbers of *wsp* and *wg* genes in whole insects based on two technical replicates.

Specimen ID	Gender	Development	Collection date	SQ Mean-*wsp*	SQ Mean-*wg*
P1	male	adult	07.07.2020	9792.82708	1362.62293
P2	male	adult	07.07.2020	43189.08681	7147.43211
P3	female	adult	07.07.2020	75429.87113	13781.91750
P4	female	adult	07.07.2020	34748.50855	4644.04911
P5	male	adult	15.02.2020	9722.07853	6012.29900
P6	female	adult	15.02.2020	63067.66415	9974.11788
P7	NA	immature	17.05.2020	991.58746	16888.23769
P8	NA	immature	17.05.2020	4814.38489	15910.69640
P41	male	adult	03.05.2020	5643.26182	9497.74361
P42	male	adult	17.05.2020	694.26488	4923.19041
P43	male	adult	30.05.2020	3309.1327	6434.00868
P44	female	adult	14.06.2020	54200.4726	9815.06083
P45	female	adult	14.06.2020	124563.9269	7365.23663
P46	female	adult	14.06.2020	21898.35606	4906.6765
P47	female	adult	26.07.2020	68794.16184	9311.48605
P48	female	adult	26.07.2020	47036.83607	10308.4938
P49	female	adult	12.07.2020	70351.5657	6107.04684
P50	female	adult	09.08.2020	49975.29203	6552.3498
P51	female	adult	09.08.2020	75450.4927	11562.28845
P52	female	adult	09.08.2020	34066.45272	7331.6883
P53	female	adult	31.01.2021	23187.1327	4904.63147
P54	male	adult	08.10.2020	19863.67008	3694.23206
P55	female	adult	08.10.2020	34350.46668	9623.24007
P56	female	adult	08.10.2020	21330.53125	7686.7668
P57	female	adult	01.11.2020	53965.83122	7418.48496
P58	male	adult	08.11.2020	0	7127.94112
P59	female	adult	15.11.2020	54984.8632	9600.5351
P60	female	adult	06.12.2020	40801.18236	6269.19535
P61	male	adult	06.12.2020	0	3845.25024

P62	female	adult	06.12.2020	41149.76197	4385.65104
P63	male	adult	03.01.2021	12727.1282	4890.15635
P64	male	adult	03.01.2021	46367.05137	5183.05977
P65	male	adult	14.02.2021	25337.79857	2433.68618
P66	female	adult	05.04.2020	101480.5524	11663.3574
P67	female	adult	19.04.2020	127470.4051	19661.02067
P68	female	adult	26.04.2020	150840.6701	16224.38794

Table S3. The list of analysed *Wolbachia* strains and corresponding supergroups. GenBank accession numbers and *wsp*/MLST gene allelic profiles are provided. GenBank numbers in bold correspond to the sequences obtained in the current study.

		GenBank accession numbers/MLST database alleles					
Host species: *Wolbachia* strain	Supergroup	*wsp*	*coxA*	*fbpA*	*ftsZ*	*gatB*	*hcpA*
Cacopsylla pyri: *w*Cpyr1 (CZ1)	B	**ON157514**	**ON146572**	**ON157499**	**ON157502**	**ON157506**	**ON157510**
Cacopsylla pyri: *w*Cpyr2 (CZ2)	B	**ON157516**	**ON146574**	**ON157501**	**ON157504**	**ON157508**	**ON157512**
Cacopsylla pyri: *w*Cpyr3 (CZ3)	B	**ON157513**	**ON146571**	**ON157498**	–	**ON157505**	**ON157509**
Parasitoid of *Cacopsylla pyri*: *w*Saph (identified as *Syrphophagus aphidivorus*)	A	**ON157515**	**ON146573**	**ON157500**	**ON157503**	**ON157507**	**ON157511**
Aedes albopictus: *w*AlbB	B	KX650069	MK809742	MK809674	MK809810	MK809606	MK809878
Agonoscena bimaculata: unnamed strain	B	MF538800	–	–	–	–	–
Agonoscena pegani: unnamed strain	B	MF538802	–	–	–	–	–
Agonoscena pistaciae: unnamed strain	B	MF538804	–	–	–	–	–
Anastrepha fraterculus: *w*AfraCast2A	A	allele 663	allele 1	allele 1	allele 3	allele 1	allele 1
Bactericera cockerelli 1: unnamed strain	B	AY971950	–	–	–	–	–
Bactericera cockerelli 2: unnamed strain	B	MW647482	–	–	–	–	–
Bactericera maculipennis: unnamed strain	B	MW647483	–	–	–	–	–
Cacopsylla fraduatrix: *w*Myr	B	MZ684135	–	–	–	–	–
Cacopsylla fraduatrix: *w*Myr04	B	MZ684134	–	–	–	–	–
Cacopsylla ledi: *w*Myr03	B	MZ684131	–	–	–	–	–
Cacopsylla myrtilli: *w*Myr02	B	MZ684110	–	–	–	–	–
Culex decens: *w*Dec	B	MK033274	MK033290	MK033314	MK033306	MK033281	MK033299
Culex quinquefasciatus: *w*Pip	B	DQ842462	DQ842278	DQ842352	DQ842314	DQ842426	DQ842389
Diaphorina citri: *w*Citri	B	GQ385975	–	–	–	–	–
Diaphorina citri 1: unnamed strain	B	MK303765	–	–	–	–	–
Diaphorina citri 2: unnamed strain	B	MN809922	–	–	–	–	–
Diaphorina citri 3: unnamed strain	B	KY472727	–	–	–	–	–
Drosophila bifasciata: *w*Bif	A	DQ842463	DQ842279	DQ842353	DQ842315	DQ842427	DQ842390
Drosophila melanogaster: *w*Mel	A	DQ842486	DQ842304	DQ842378	DQ842340	DQ842452	DQ842415
Drosophila simulans: *w*Ma	B	allele 15	allele 4	allele 6	allele 4	allele 5	allele 5
Drosophila simulans: *w*No	B	DQ842470	DQ842287	DQ842361	DQ266426	DQ842435	DQ842398
Erebia jeniseiensis: *w*Ejen	B	allele 521	allele 173	allele 27	allele 106	allele 39	allele 6

Eurema blanda: *w*Ebl	B	–	MZ821089	MZ821105	MZ821121	MZ821137	MZ821153
Euwallacea interjectus: *w*Ei	A	AB588925	LC062513	LC062516	LC062515	LC062512	LC062514
Folsomia candida: *w*Fcan	E	KT799615	KT799590	KT799595	KT799600	KT799605	KT799610
Megastigmus pistaciae: *w*B	B	KF531897	KF531864	–	KF531888	KF531872	KF531880
Neptis rivularis: *w*Nriv1	A	allele 374	allele 14	allele 4	allele 36	allele 9	allele 227
Ostrinia scapulalis: *w*OscaB	B	DQ842481	DQ842298	DQ842372	DQ842334	DQ842446	DQ842409
Philaenus spumarius: unnamed strain	B	–	KM377691	KM377739	KM377767	KM377776	KM377678
Psyttalia carinata: *w*Rha	A	KX503396	KX503398	KX503401	KX503400	KX503397	KX503399
Tribolium confusum: *w*Con	B	DQ842484	DQ842301	DQ842375	DQ842337	DQ842449	DQ842412
Trichogramma deion: *w*Dei	B	–	DQ842302	DQ842376	DQ842338	DQ842450	DQ842413
Unnamed host: *w*APS122	A	allele 79	allele 48	allele 60	allele 6	allele 58	allele 60
Xyleborus seiryorensis: *w*Xse	A	AB588929	LC062523	LC062526	LC062525	LC062522	LC062524
Xylosandrus germanus: *w*Xge1	A	AB359039	allele 77	allele 61	allele 55	allele 53	allele 96

Supplementary File 1. Alignment of the *wsp* gene sequences used for the phylogenetic analysis (**Figs 2A**, S2A).
>wCpyr3
ACAAGAATTGATGGTATTGAACATACATCAKGTCAA------------
AGTCCCTTAAAAGCATCTTTTATAGCTGGTGGTGGTGCATTTGGTTATAAAATGGACGACATTRSAGTTGATGTTGAAGGGCTTTACTCACAAT
TGGCTAAAGATGCAACT------GTAGTA------
TCTGGTGACAGGGCTGCAGATAGTGTAACAGCATTTTCAGGATTGGTTAACGTTTATTACGATATAGCT---
ATTGAAGATATGCCTATCACTCCATACGTTGGTGTTGGTGTTGGTGCAGCATGTATCAGCAATCCTTCAAAAGCTGATGCAGTTAAAGATCAA
AAAGGATTTGGTTTTGCTTATCAAGCAAAAGCTGGTGTTAGTTATGATGTAACTCCAGAAATCAAACTCTTTGCTGGTGCTCGTTATTTTGGTT
CTTATGGTGCTAGTTTTAATAAAGAAACAGTATCAGCT
>wCpyr2
ACAAGAATTGACGGCATTGAATATAAAAAAGGAGACGAA---
GTTCATGATCCTTTAAAAGCATCTTTTATGGCTGGTGGTGCTGCATTTGGTTATAAAATGGACGATATCAGGGTTGATGTTGAGGGACTTTACT
CACAACTAAACAAAAACGACGTTAGTGGTGCAACATTTA---
CTCCAACAACTGTTGCAAACAGTGTGGCAGCATTTTCAGGATTGGTTAACGTTTATTACGATATAGCG---
ATTGAAGATATGCCTATCACTCCATACGTTGGTGTTGGTGTTGGTGCAGCATATATCAGCAATCCTTCAGAAGCTAGTGCAGTTAAAGATCAA
AAAGGATTTGGTCTTGCTTATCAAGCAAAAGCTGGTGTTAGTTATGATGTAACCCCAGAAATCAAGCTTTATGCTGGTGCTCGTTATTTTGGTT
CTTATGGTGCTAGTTTTAATAAAGAAGCAGTATCAGCT
>wCpyr1
ACAAGAATTGACGGCATTGAATATAAAAAAGGAGACGAA---
GTTCATGATCCTTTAAAAGCATCTTTTATGGCTGGTGGTGCTGCATTTGGTTATAAAATGGACGATATCAGGGTTGATGTTGAGGGACTTTACT
CACAACTAAACAAAAACGACGTTAGTGGTGCAACATTTA---
CTCCAACAACTGTTGCAAACAGTGTGGCAGCATTTTCAGGATTGGTTAACGTTTATTACGATATAGCG---
ATTGAAGATATGCCTATCACTCCATACGTTGGTGTTGGTGTTGGTGCAGCATATATCAGCAATCCTTCAGAAGCTAGTGCAGTTAAAGATCAA
AAAGGATTTGGTCTTGCTTATCAAGCAAAAGCTGGTGTTAGTTATGATGTAACCCCAGAAATCAAGCTTTATGCTGGTGCTCGTTATTTTGGTT
CTTATGGTGCTAGTTTTAATAAAGAAGCAGTATCAGCT
>wSaph
ACAAAAGTTGATGGTATTACCTATAAGAAAGACAAGAGT---
GATTACAGTCCATTAAAACCATCTTTTATAGCTGGTGGTGGTGCATTTGGTTACAAAATGGACGACATCAGGGTTGATGTTGAAGGAGTTTAT
TCATACCTAAACAAAAATSATGTTAAAGGTGTAACATTTGACCCAGCAAATACTATTGCAGACAGTGTAACAGCAATTTCAGGATTAGTGAAC
GTGTATTACGATATAGCA---
ATTGAAGATATGCCTATCACTCCATACATTGGTGTTGGTGTTGGTGCAGCGTATATTAGCACTCCTTTGGAACCCGCTGTGAATGATCAAAAA
AGTAAATTTGGTTTTGCTGGTCAAGTAAAAGCTGGTGTTAGYTATGATGTAACTCCAGAARTCAAACTTTATGCTGGAGCTCGTTATTTCGGTT
CTTATGGTGCTAATTTTGATGGAAAAAAAACAGATCCT
>Megastigmus_pistaciae_wB
ACAAAGATTGATGGTATTACATATACATCAGGTCAA------------
AGTCCCTTAAAAGCATCTTTTATAGCTGGTGGTGGTGCATTTGGTTATAAAATGGACGACATTAGAGTTGATGTTGAAGGGCTTTACTCACAA
TTGGCTAAAGATGCAACT------GTAGTA------
TCTGATAACAAGGCTGCAGATAGTGTAATAGCATTTTCAGGATTGGTTAACGTTTATTACGATATAGCG---
ATTGAAGATATGCCTATCACTCCATACGTTGGTGTTGGTGTTGGTGCAGCATATATCAGCAATCCTTCAAAAGCTGATGCAGTTAAAGAGCAA
AAAGGATTTGGTTTTGCTTATCAAGCAAAAGCTGGTGTTAGTTATGATGTAACTCCAGAAATCAAACTCTTTGCTGGTGCTCGTTATTTTGGTT
CTTATGGTGCTAGTTTTAATAAAGAAACAGTATCAGCT
>Erebia_jeniseiensis_Ejen

ACAAAGATTGATGGTATTACACATACATCAGGTCAA------------
AGTCCCTTAAAAGCATCTTTTATAGCTGGTGGTGGTGCATTTGGTTATAAAATGGACGACATTAGAGTTGATGTTGAAGGGCTTTACTCACAA
TTGGCTAAAGATGCAACT------GTAGTA------
TCTGATAACAAGGCTGCAGATAGTGTAACAGCATTTTCAGGATTGGTTAACGTTTATTACGATATAGCG---
ATTGAAGATATGCCTATCACTCCATACGTTGGTGTTGGTGTTGGTGCAGCATATATCAGCAATCCTTCAAAAGCTGATGCAGTTAAAGAGCAA
AAAGGATTTGGTTTTGCTTATCAAGCAAAAGCTGGTGTTAGTTATGATGTAACTCCAGAAATCAAACTCTTTGCTGGTGCTCGTTATTTTGGTT
CTTATGGTGCTAGTTTTAATAAAGAAACAGTATCAGCT
>Culex_decens_wDec
ACAAAAGTTGATGGTATTACAAAGGCAACAGGTAAAGAA---
AAGGATAGTCCCTTAACAAGATCTTTTATAGCTGGTGGTGGTGCATTTGGTTATAAAATGGATGACATTAGAGTTGATGTTGAAGGGCTTTAC
TCACAATTGACTAAAGATGCAACT------GTAGTA------
TCTGATAACAGCGCTGCAGATAGTGTAACAGCATTCTCAGGATTGGTTAACGTTTATTACGATATAGCG---
ATTGAAGATATGCCTATCACTCCATACGTTGGTGTTGGTGTTGGTGCAGCATATATCAGCAATCCTTCAAAAGTTGATGCAGTTAAAGAGCAA
AAAAGATTTGGTTTTGCTTATCAAGCAAAAGCTGGTGTTAGTTATGATGTAACCTCAGAAATCAAGCTTTATGCTGGTGCTCGTTATTTTGGTT
CTTATGGTGCTAGTTTTAATAAAGAAACAGTATCAGCT
>Ostrinia_scapulalis_wOscaB
ACAAAAGTTGATGGTATTACAAATGCAACAGGTAAACAA---
AAGGATAGTCCCTTTACAAGATCTTTTATAGCTGGTGGTGGTGCATTTGGTTATAAAATGGATGACATTAGAGTTGATGTTGAAGGGCTTTAC
TCACAATTGGCTAAAGATACAGCT------
GTAGTAAATACTTCTGAAACAAATGTTGCAGACAGTTTAACAGCATTTTCAGGATTGGTTAACGTTTATTACGATATAGCG---
ATTGAAGATATGCCTATCACTCCATACGTTGGTGTTGGTGTTGGTGCAGCATATATCAGCAATCCTTCAAAAGCTGATACAGTTAAAGATCAA
AAAGGATTTGGTTTTGCTTATCAAGCAAAAGCTGGTGTTAGTTATGATGTAACCCCAGAAATCAAACTCTTTGCTGGAGCTCGTTACTTCGGTT
CTTATGGTGCTAGTTTTGATAAGACAAATAAGGATAAT
>Tribolium_confusum_wCon
ACAAAAGTTGAGGGTATTACAAATGTAACAGGTAAAGAA---
AAAGATAGTCCCTTAACAAGATCTTTTATAGCTGGTGGTGGTGCATTTGGTTATAAAATGGACGACATTAGAGTTGATGTTGAAGGGCTTTAC
TCACAATTGGCTAAAGATACAGCT------
GTAGTAAATACTTCTGAAACAAATGTTGCAGACAGTTTAACAGCATTTTCAGGATTGGTTAACGTTTATTACGATATAGCG---
ATTGAAGATATGCCTATCACTCCATACGTTGGTGTTGGTGTTGGTGCAGCATATATCAGCAATCCTTCAAAAGCTGATGCAGTTAAAGATCAA
AAAGGATTTGGTTTTGCTTATCAAGCAAAAGCTGGTGTTAGCTATGATGTAACTCCAGAAATCAAACTCTTTGCTGGAGCTCGTTACTTCGGTT
CTTATGGTGCTAGTTTTGATAAGGCGGCTAAGGATGAT
>Drosophila_simulans_wNo
ACAAGAATTGACGGCATTGAATATAAAAAAGGAACCGAA---
GTTCATGATCCTTTAAAAGCATCTTTTATGGCTGGTGGTGCTGCATTTGGTTATAAAATGGACGATATCAGGGTTGATGTTGAGGGACTTTACT
CACAACTAAACAAAAACGACGTTAGTGGTGCAACATTTA---
CTCCAACAACTGTTGCAAACAGTGTGGCAGCATTTTCAGGATTGGTTAACGTTTATTACGATATAGCG---
ATTGAAGATATGCCTATCACTCCATACGTTGGTGTTGGTGTTGGTGCAGCATATATCAGCAATCCTTCAGAAGCTAGTGCAGTTAAAGATCAA
AAAGAATTTGGTTTTGCTTATCAAGCAAAAGCTGGTGTTAGTTATGATGTAACCCCAGAAATCAAGCTTTATGCTGGTGCTCGTTATTTTGGTT
CTTATGGTGCTAGTTTTAATAAAGAAGCAGTATCAGCT
>Drosophila_simulans_wMA
ACAAGAATTGACGGCATTGAATATAAAAAAGGAACCGAA---
GTTCATGATCCTTTAAAAGCATCTTTTATGGCTGGTGGTGCTGCATTTGGTTATAAAATGGACGATATCAGGGTTGATGTTGAGGGACTTTACT
CACAACTAAACAAAAACGACGTTAGTGGTGCAACATTTA---

CTCCAACAACTGTTGCAAACAGTGTGGCAGCATTTTCAGGATTGGTTAACGTTTATTACGATATAGCG---
ATTGAAGATATGCCTATCACTCCATACGTTGGTGTTGGTGTTGGTGCAGCATATATCAGCAATCCTTCAGAAGCTAGTGCAGTTAAAGATCAA
AAAGAATTTGGTTTTGCTTATCAAGCAAAAGCTGGTGTTAGTTATGATGTAACCCCAGAAATCAAGCTTTATGCTGGTGCTCGTTATTTTGGTT
CTTATGGTGCTAGTTTTAATAAAGAAGCAGTATCAGCT
>Culex_quinquefasciatus_wPip
ACAAGAATTGACGGCATTGAATATAAAAAAGGAACCGAA---
GTTCATGATCCTTTAAAAGCATCTTTTATGGCTGGTGGTGCTGCATTTGGTTATAAAATGGACGATATCAGGGTTGATGTTGAGGGACTTTACT
CACAACTAAACAAAAACGACGTTAGTGGTGCAACATTTA---
CTCCAACAACTGTTGCAAACAGTGTGGCAGCATTTTCAGGATTGGTTAACGTTTATTACGATATAGCG---
ATTGAAGATATGCCTATCACTCCATACGTTGGTGTTGGTGTTGGTGCAGCATATATCAGCAATCCTTCAGAAGCTAGTGCAGTTAAAGATCAA
AAAGGATTTGGTTTTGCTTATCAAGCAAAAGCTGGTGTTAGTTATGATGTAACCCCAGAAATCAAACTCTTTGCTGGTGCTCGTTATTTTGGTT
CTTATGGTGCTAGTTTTAATAAAGAAGCAGTATCAGCT
>Aedes_albopictus_wAlbB
ACAAGAATTGACGGCATTGAATATAAAAAAGGAACCGAA---
GTTCATGATCCTTTAAAAGCATCTTTTATGGCTGGTGGTGCTGCATTTGGTTATAAAATGGACGATATCAGGGTTGATGTTGAGGGACTTTACT
CACAACTAAACAAAAACGACGTTGGTGGTGCAACATTTG---
CTCCAACAACTGTTGCAAACAGTGTGGCAGTATTTTCAGGATTGGTTAACGTTTATTACGATATAGCG---
ATTGAAGATATGCCTATCACTCCATACGTTGGTGTTGGTGTTGGTGCAGCATATATCAGCAATCCTTCAGAAGCTAGTGCAGTTAAAGATCAA
AAAGGATTTGGTTTTGCTTATCAAGCAAAAGCTGGTGTTAGTTATGATGTAACCCCAGAAATCAAGCTTTATGCTGGTGCTCGTTATTTTGGTT
CTTATGGTGCTAGTTTTAATAAAGAAACAGTATCAGCT
>Drosophila_melanogaster_wMel
ACAAAAGTTGATGGTATTACCTATAAGAAAGACAAGAGT---
GATTACAGTCCATTAAAACCATCTTTTATAGCTGGTGGTGGTGCATTTGGTTACAAAATGGACGACATCAGGGTTGATGTTGAAGGAGTTTAT
TCATACCTAAACAAAAATGATGTTAAAGATGTAACATTTGACCCAGCAAATACTATTGCAGACAGTGTAACAGCAATTTCAGGATTAGTGAA
CGTGTATTACGATATAGCA---
ATTGAAGATATGCCTATCACTCCATACATTGGTGTTGGTGTTGGTGCAGCGTATATTAGCACTCCTTTGGAACCCGCTGTGAATGATCAAAAA
AGTAAATTTGGTTTTGCTGGTCAAGTAAAAGCTGGTGTTAGTTATGATGTAACTCCAGAAGTCAAACTTTATGCTGGAGCTCGTTATTTCGGTT
CTTATGGTGCTAATTTTGATGGAAAAAAAACAGATCCT
>Xylosandrus_germanus_wXge1
ACAAAAGTTGATGGTATTACCTATAAGAAAGACAAGAGT---
GATTACAGTCCATTAAAACCATCTTTTATAGCTGGTGGTGGTGCATTTGGTTACAAAATGGACGACATCAGGGTTGATGTTGAAGGAGTTTAT
TCATACCTAAACAAAAATGATGTTAAAGATGTAACATTTGACCCAGCAAATACTATTGCAGACAGTGTAACAGCAATTTCAGGATTAGTGAA
CGTGTATTACGATATAGCA---
ATTGAAGATATGCCTATCACTCCATACATTGGTGTTGGTGTTGGTGCAGCGTATATTAGCACTCCTTTGGAACCCGCTGTGAATGATCAAAAA
AGTAAATTTGGTTTTGCTGGTCAAGTAAAAGCTGGTGTTAGTTATGATGTAACTCCAGAAGTCAAACTTTATGCTGGAGCTCGTTATTTCGGTT
CTTATGGTGCTAATTTTGATGGAAAAAAAACAGATCCT
>Euwallacea_interjectus_wEi
ACAAAAGTTGATGGTATTACCTATAAGAAAGACAAGAGT---
GATTACAGTCCATTAAAACCATCTTTTATAGCTGGTGGTGGTGCATTTGGTTACAAAATGGACGACATCAGGGTTGATGTTGAAGGAGTTTAT
TCATACCTAAACAAAAATGATGTTAAAGATGTAACATTTGACCCAGCAAATACTATTGCAGACAGTGTAACAGCAATTTCAGGATTAGTGAA
CGTGTATTACGATATAGCA---
ATTGAAGATATGCCTATCACTCCATACATTGGTGTTGGTGTTGGTGCAGCGTATATTAGCACTCCTTTGGAACCCGCTGTGAATGATCAAAAA

AGTAAATTTGGTTTTGCTGGTCAAGTAAAAGCTGGTGTTAGTTATGATGTAACTCCAGAAGTCAAACTTTATGCTGGAGCTCGTTATTTCGGTT
CTTATGGTGCTAATTTTGATGGAAAAAAAACAGATCCT
>Psyttalia_rhagoleticola_wRha
ACAAAAGTTGATGGTATTACCTATAAGAAAGACAAGAGT---
GATTACAGTCCATTAAAACCATCTTTTATAGCTGGTGGTGGTGCATTTGGTTACAAAATGGACGACATCAGGGTTGATGTTGAAGGAGTTTAT
TCATACCTAAACAAAAATGATGTTAAAGATGTAACATTTGACCCAGCAAATACTATTGCAGACAGTGTAACAGCAATTTCAGGATTAGTGAA
CGTGTATTACGATATAGCA---
ATTGAAGATATGCCTATCACTCCATACATTGGTGTTGGTGTTGGTGCAGCGTATATTAGCACTCCTTTGGAACCCGCTGTGAATGATCAAAAA
AGTAAATTTGGTTTTGCTGGTCAAGTAAAAGCTGGTGTTAGTTATGATGTAACTCCAGAAGTCAAACTTTATGCTGGAGCTCGTTATTTCGGTT
CTTATGGTGCTAATTTTGATGGAAAAAAAACAGATCCT
>Anastrepha_fraterculus_wAfraCast2A
ACAAAAGTTGATGGTATTACCTATAAGAAAGACAAGAGT---
GATTACAGTCCATTAAAACCATCTTTTATAGCTGGTGGTGGTGCATTTGGTTACAAAATGGACGACATCAGGGTTGATGTTGAAGGAGTTTAT
TCATACCTAAACAAAAATGATGTTAAAGATGTAACATTTGACCCAGCAAATACTATTGCAGACAGTGTAACAGCAATTTCAGGATTAGTGAA
CGTGTATTACGATATAGCA---
ATTGAAGATATGCCTATCACTCCATACATTGGTGTTGGTGTTGGTGCAGCGTATATTAGCACTCCTTTGGAACCCGCTGTGAATGATCAAAAA
AGTAAATTTGGTTTTGCTGGTCAAGTAAAAGCTGGTGTTAGTTATGATGTAACTCCAGAAGTCAAACTTTATGCTGGAGCTCGTTATTTCGGTT
CTTATGGTGCTAATTTTGATGGAAAAAAAACAGATCCT
>Xyleborus_seiryorensis_wXse
ACAAAAGTTGATGGTATTACCTATAAGAAAGACAAGAGT---
GATTACAGTCCATTAAAACCATCTTTTATAGCTGGTGGTGGTGCATTTGGTTACAAAATGGACGACATCAGGGTTGATGTTGAAGGAGTTTAT
TCATACCTAAACAAAAATGATGTTAAAGATGTAACATTTGACCCAGCAAATACTATTGCAGACAGTGTAACAGCAATTTCAGGATTAGTGAA
CGTGTATTACGATATAGCA---
ATTGAAGATATGCCTATCACTCCATACATTGGTGTTGGTGTTGGTGCAGCGTATATTAGCACTCCTTTGGAACCAGCTGTGAATGATCAAAAA
AGTAAATTTGGTTTTGCTGGTCAAGTAAAAGCTGGTGTTAGTTATGATGTAACTCCAGAAGTCAAACTTTATGCTGGAGCTCGTTATTTCGGTT
CTTATGGTGCTAATTTTGATGGAAAAAAAACAGATCCT
>Drosophila_bifasciata_wBif
ACAAAAGTTGATGGTATTACCTATAAGAAAGACAATAGT---
GATTACAGTCCATTAAAAGCGTCTTTTATAGCTGGTGGTGGTGCGTTTGGTTACAAAATGGACGACATCAGGGTTGATGTTGAAGGAGTTTAT
TCATACCTAAACAAAAATGATGTTACAGATGCAAAATTTA---
CGCCAGATACTATTGCAGACAGTTTAACAGCAATTTCAGGACTAGTTAACGTTTATTACGATATAGCA---
ATTGAAGATATGCCTATCACTCCATATATTGGTGTTGGTGTTGGTGCAGCGTATATTAGCACTCCTTTGAAAGACGCTGTGAATGATCAAAAA
AGTAAATTTGGTTTTGCTGGTCAAGTAAAAGCTGGTGTTAGTTATGATGTAACTCCGGAAGTCAAACTTTATGCTGGAGCTCGTTATTTCGGTT
CTTTTGGTGCTCATTTTGATAAAGATGCTGCTGCAGGC
>Folsomia_candida_wFcan
ACAAAGATTGATACATTTACAGCTACAAAAGATAGCGATACCACTCTTGATCCTTTTAAGGCTTCTTTCATAGGTGGTGGAGCCGAATTTGGT
TACAAAATGGAGGACTTCAGAGTTGGAGTTGAAGGGGTTTACTCACAATTGAATAAGAATGCTGACACAGGTGTAGTGCTTGCACCTGCAGA
TGCAGCTGCAGAAAAATTAACAGCAATTGCAGTATTAGTTAATGTGAATTACGATATAGCAATTATTGAAGATCTGCCTGTAGCTCCATACGT
TGGTGTGGGTGTTGGTGCAGCATATATCAACAATCCTTTAAAAGCTCCTTTTAATGAGCAAAAAAGTGGATTCGGTGTTGCTTATCAAGCAAA
AGCTGGTGTTAGTTATGCTGTAACCCCAGAAATTAATCTCTATGCTGGTGCTCGCTACTTCGGCTCTTATGGTGCTAAATTTGATAAAAGTGGT
GAGGAAGGT
>A_PS122

ACAAAAGTTGATGGTATTACCTATAAGAAAGACAATAGT---
GATTACAGTCCATTAAAAGCGTCTTTTATAGCTGGTGGTGGTGCGTTTGGTTACAAAATGGACGACATCAGGGTTGATGTTGAAGGAGTTTAT
TCATACCTAAACAAAAATGATGTTACAGATGCACAATTTA---
CGCCAGATACTATTGCAGACAGTTTAACAGCAATTTCAGGACTAGTTAACGTTTATTACGATATAGCA---
ATTGAAGATATGCCTATCACTCCATATATTGGTGTTGGTGTTGGTGCAGCGCATATTAGCACTCCTTTGAAAGACGCTGTGAATGATCAAAAA
AGTAAATTTGGTTTTGCTGGTCAAGTAAAAGCTGGTGTTAGTTATGATGTAACTCCGGAAGTCAAACTTTATGCTGGAGCTCGTTATTTCGGTT
CTTTTGGTGCTCATTTTGATAAAGATGCTGCTGCAGGC

Supplementary File 2. Alignment of the *wsp* gene sequences used for the phylogenetic analysis, including the psyllid-associated *Wolbachia* strains (Figure S3).
>wCpyr3
ACAAGAATTGATGGTATTGAACATACATCAKGTCAA------------
AGTCCCTTAAAAGCATCTTTTATAGCTGGTGGTGGTGCATTTGGTTATAAAATGGACGACATTRSAGTTGATGTTGAAGGGCTTTACTCACAAT
TGGCTAAAGATGCAAC------TGTAGTA------
TCTGGTGACAGGGCTGCAGATAGTGTAACAGCATTTTCAGGATTGGTTAACGTTTATTACGATATAGCT---
ATTGAAGATATGCCTATCACTCCATACGTTGGTGTTGGTGTTGGTGCAGCATGTATCAGCAATCCTTCAAAAGCTGATGCAGTTAAAGATCAA
AAA---
GGATTTGGTTTTGCTTATCAAGCAAAAGCTGGTGTTAGTTATGATGTAACTCCAGAAATCAAACTCTTTGCTGGTGCTCGTTATTTTGGTTCTT
ATGGTGCTAGTTTTAATAAAGAAACAGTATCAGCT
>Megastigmus_pistaciae_wB
ACAAAGATTGATGGTATTACATATACATCAGGTCAA------------
AGTCCCTTAAAAGCATCTTTTATAGCTGGTGGTGGTGCATTTGGTTATAAAATGGACGACATTAGAGTTGATGTTGAAGGGCTTTACTCACAA
TTGGCTAAAGATGCAAC------TGTAGTA------
TCTGATAACAAGGCTGCAGATAGTGTAATAGCATTTTCAGGATTGGTTAACGTTTATTACGATATAGCG---
ATTGAAGATATGCCTATCACTCCATACGTTGGTGTTGGTGTTGGTGCAGCATATATCAGCAATCCTTCAAAAGCTGATGCAGTTAAAGAGCAA
AAA---
GGATTTGGTTTTGCTTATCAAGCAAAAGCTGGTGTTAGTTATGATGTAACTCCAGAAATCAAACTCTTTGCTGGTGCTCGTTATTTTGGTTCTT
ATGGTGCTAGTTTTAATAAAGAAACAGTATCAGCT
>Erebia_jeniseiensis_Ejen
ACAAAGATTGATGGTATTACACATACATCAGGTCAA------------
AGTCCCTTAAAAGCATCTTTTATAGCTGGTGGTGGTGCATTTGGTTATAAAATGGACGACATTAGAGTTGATGTTGAAGGGCTTTACTCACAA
TTGGCTAAAGATGCAAC------TGTAGTA------
TCTGATAACAAGGCTGCAGATAGTGTAACAGCATTTTCAGGATTGGTTAACGTTTATTACGATATAGCG---
ATTGAAGATATGCCTATCACTCCATACGTTGGTGTTGGTGTTGGTGCAGCATATATCAGCAATCCTTCAAAAGCTGATGCAGTTAAAGAGCAA
AAA---
GGATTTGGTTTTGCTTATCAAGCAAAAGCTGGTGTTAGTTATGATGTAACTCCAGAAATCAAACTCTTTGCTGGTGCTCGTTATTTTGGTTCTT
ATGGTGCTAGTTTTAATAAAGAAACAGTATCAGCT
>Culex_decens_wDec
ACAAAAGTTGATGGTATTACAAAGGCAACAGGTAAAGAAAAGG---
ATAGTCCCTTAACAAGATCTTTTATAGCTGGTGGTGGTGCATTTGGTTATAAAATGGATGACATTAGAGTTGATGTTGAAGGGCTTTACTCAC
AATTGACTAAAGATGCAAC------TGTAGTA------
TCTGATAACAGCGCTGCAGATAGTGTAACAGCATTCTCAGGATTGGTTAACGTTTATTACGATATAGCG---
ATTGAAGATATGCCTATCACTCCATACGTTGGTGTTGGTGTTGGTGCAGCATATATCAGCAATCCTTCAAAAGTTGATGCAGTTAAAGAGCAA
AAA---
AGATTTGGTTTTGCTTATCAAGCAAAAGCTGGTGTTAGTTATGATGTAACCTCAGAAATCAAGCTTTATGCTGGTGCTCGTTATTTTGGTTCTT
ATGGTGCTAGTTTTAATAAAGAAACAGTATCAGCT
>Ostrinia_scapulalis_wOscaB
ACAAAAGTTGATGGTATTACAAATGCAACAGGTAAACAAAAGG---
ATAGTCCCTTTACAAGATCTTTTATAGCTGGTGGTGGTGCATTTGGTTATAAAATGGATGACATTAGAGTTGATGTTGAAGGGCTTTACTCACA
ATTGGCTAAAGATACAGC------
TGTAGTAAATACTTCTGAAACAAATGTTGCAGACAGTTTAACAGCATTTTCAGGATTGGTTAACGTTTATTACGATATAGCG---

ATTGAAGATATGCCTATCACTCCATACGTTGGTGTTGGTGTTGGTGCAGCATATATCAGCAATCCTTCAAAAGCTGATACAGTTAAAGATCAA
AAA---
GGATTTGGTTTTGCTTATCAAGCAAAAGCTGGTGTTAGTTATGATGTAACCCCAGAAATCAAACTCTTTGCTGGAGCTCGTTACTTCGGTTCTT
ATGGTGCTAGTTTTGATAAGACAAATAAGGATAAT
>Tribolium_confusum_wCon
ACAAAAGTTGAGGGTATTACAAATGTAACAGGTAAAGAAAAAG---
ATAGTCCCTTAACAAGATCTTTTATAGCTGGTGGTGGTGCATTTGGTTATAAAATGGACGACATTAGAGTTGATGTTGAAGGGCTTTACTCAC
AATTGGCTAAAGATACAGC------
TGTAGTAAATACTTCTGAAACAAATGTTGCAGACAGTTTAACAGCATTTTCAGGATTGGTTAACGTTTATTACGATATAGCG---
ATTGAAGATATGCCTATCACTCCATACGTTGGTGTTGGTGTTGGTGCAGCATATATCAGCAATCCTTCAAAAGCTGATGCAGTTAAAGATCAA
AAA---
GGATTTGGTTTTGCTTATCAAGCAAAAGCTGGTGTTAGCTATGATGTAACTCCAGAAATCAAACTCTTTGCTGGAGCTCGTTACTTCGGTTCTT
ATGGTGCTAGTTTTGATAAGGCGGCTAAGGATGAT
>Bactericera_maculipennis
ACAAAAGTTGATGGTATTACAAATGCAACAGGTAAAGAAAAGG---
ATAGTCCATTAACAAGATCTTTTATAGCTGGTGGTGGTGCATTTGGTTATAAAATGGATGACATTAGAGTTGATGTTGAAGGGCTTTACTCAC
AATTGGCTAAAGATACAGC------
TGTAGTAAATACTTCTGAAACAAATGTTGCAGACAGTTTAACAGCATTTTCAGGATTGGTTAACGTTTATTACGATATAGCG---
ATTGAAGATATGCCTATCACTCCATACGTTGGTGTTGGTGTTGGTGCAGCATATATCAGCAATCCTTCAAAAGCTGATGCAGTTAAAGATCAA
AAA---
GGATTTGGTTTTGCTTATCAAGCAAAAGCTGGTGTTAGCTATGATGTAACTCCAGAAATCAAACTCTTTGCTGGAGCTCGTTACTTCGGTTCTT
ATGGTGCTAGTTTTGATAAGGCAGCTAAGGATGAT
>Diaphorina_citri_wCitri
ACAAAAGTTGATGGTATTACAAATGCAACAGGTAAAGAAAAGG---
ATAGTCCCTTAACAAGATCTTTTATAGCTGGTGGTGGTGCATTTGGTTATAAAATGGATGACATTAGAGTTGATGTTGAAGGGCTTTACTCAC
AATTGGCTAAAGATACAGC------
TGTAGTAGATACTTCTGAAACAAATGTTGCAGACAGTTTAACAGCATTTTCAGGATTGGTTAACGTTTATTACGATATAGCG---
ATTGAAGATATGCCTATCACTCCATACGTTGGTGTTGGTGTTGGTGCAGCATATATCAGCAATCCTTCAAAAGCTGATGCAGTTAAAGATCAA
AAA---
GGATTTGGTTTTGCTTATCAAGCAAAAGCTGGTGTTAGTTATGATGTAACTCCAGAAATCAAACTCTTTGCTGGAGCTCGTTACTTCGGTTCTT
ATGGTGCTAGTTTTGATAAGGCAGCTAAGGATGAT
>Bactericera_cockerelli 2
ACAAAAGTTGATGGTATTAAAAATGCAAAAGATAAAGAAAAGG---
ATAGTCCCTTAACAAGATCTTTTATAGCTGCTGGTGGTGCATTTGGTTATAAAATGGATGACATTAGAGTTGATGTTGAAGGGCTTTACTCAC
AATTGGCTAAAGATACAGC------
TGTAGTAGATACTTCTGAAACAAATGTTGCAGACAGTTTAACAGCATTTTCAGGATTGGTTAACGTTTATTATGATATAGCG---
ATTGAAGATATGCCTATCACTCCATACGTTGGTGTTGGTGTTGGTGCAGCATATATCAGCAATCCTTCAAAAGCTGATACAGTTAAAGATCAA
AAA---
GGATTTGGTTTTGCTTATCAAGCAAAAGCTGGTGTTAGCTATGATGTAACTCCAGAAATCAAACTCTTTGCTGGAGCTCGTTACTTCGGTTCTT
ATGGTGCTAGTTTTGATAAGGCAGCTAACGATGAT
>Diaphorina_citri 3
-------TTGATGGTATTACAAATGCAACAGGTAAAGAAAAGG---
ATAGTCCCTTAACAAGATCTTTTATAGCTGGTGGTGGTGCATTTGGTTATAAAATGGATGACATTAGAGTTGATGTTGAAGGGCTTTACTCAA

AATTGGCTAAAGATACAGA------
TGTAGTAAATACTTCTGAAACAAATGTTGCAGACAGTTTAACAGCATTTTCAGGATTGGTTAACGTTTATTACGATATAGCG---
ATTGAAGATATGCCTATCACTCCATACGTTGGTGTTGGTATTGGTGCAGCATATATCAGCAATCCTTCAAAAGCTGATGTAGTTAAAGATCAA
AAA---
GGATTTGGTTTTGCTTATCAAGCAAAAGCTGGTGTTAGCTATGATGTAACTCCAGAAATCAAACTCTTTGCTGGAGCTCGTTACTTCGGTTCTT
ATGGTGCTAGTTTTGATAAGGCAGCTAAGGATGAT
>Diaphorina_citri 2
ACAAAAGTTGATGGTATTACAAATGCAACAGGTAAAGAAAAGG---
ATAGTCCCTTAACAAGATCTTTTATAGCTGGTGGTGGTGCATTTGGTTATAAAATGGATGACATTAGAGTTGATGTTGAAGGGCTTTACTCAA
AATTGGCTAAAGATACAGA------
TGTAGTAAATACTTCTGAAACAAATGTTGCAGACAGTTTAACAGCATTTTCAGGATTGGTTAACGTTTATTACGATATAGCG---
ATTGAAGATATGCCTATCACTCCATACGTTGGTGTTGGTATTGGTGCAGCATATATCAGCAATCCTTCAAAAGCTGATGTAGTTAAAGATCAA
AAA---
GGATTTGGTTTTGCTTATCAAGCAAAAGCTGGTGTTAGCTATGATGTAACTCCAGAAATCAAACTCTTTGCTGGAGCTCGTTACTTCGGTTCTT
ATGGTGCTAGTTTTGATAAGGCAGCTAAGGATGAT
>MF538804_Agonoscena_pistaciae
---------------------AATGCAAGAGGTAAAGAAAAGG---
ATAGTCCCTTAACAAGATCTTTTATAGCTGGTGATGGTGCATTTGGTTATAAAATGGATGACATTAGAGTTGATGTTGAAGGGCTTTACTCAC
AATTGGCTAAAGATACAGCTA---
GTAGTACAATGCTTACTGAAACAAATGTTGCAGACAGTTTAACAGCATTTTCAGGATTGGTTAACGTTTATTACGATATAGCG---
ATTGAAGATATGCCTATCACTCCGTACGTTGGTGTTGGTGTTGGTGCAGCATATATCAGCAATCCTTCAAAAGCTGATGCAGTTAAAGATCAA
AAA---
GGATTTGGTTTTGCTTATCAAGCAAAAGCTGGTGTGAGTTATGATGTAACTCCAGAAATCAAACTCTTTGCTGGAGCTCGTTACTTCGGTTCTT
ATGGTGCTAGT------------------------
>Diaphorina_citri 1
ACAAAAGTTGTTGGTATTACAAATGCAACAGGTATAGAAAAGG---
ATACTCCCTTATCAAGATCTTTTATACCTGGTGGTGGTGCATTTGGTTATATAATGGATGTCATTATAGTTGTTGTTGTAGGGCTTTACTCACA
ATTGGCTATAGATACAGC------
TGTACTATATACTTCTGTAACAAATGTTGCAGACAGTTTATCAGCATTTTCAGGATTGGTTATCGTTTATTACGATATACCG---
ATTGTAGATATGCCTATCACTCCATACGTTGGTGTTGGTGTTGGTGCAGCATATATCAGCAATCCTTCAAAAGCTGTTGCAGTTATAGATCAA
AAA---
GGATTTGGTTTTGCTTATCAAGCAAAAGCTGGTGTTTGTTATGTTGTATCTCCAGAAATCAAACTCTTTGCTGGAGCTCGTTACTTCGGTTCTT
ATGGTGCTACTTTTGTTATGGCAGCTATGGATGTT
>Cacopsylla_fraudatrix_wMyr
ACAAAGATTGATGGTGTTACATATAAATCAGGTAAGGAGAACA---
ATAGTCCCTTAAAAGCATCTTTTCTAGCTGGAGGTGGTGCATTTGGTTATAAAATGGATGATATCAGGGTTGATGTTGAAGGACTTTACTCAC
AATTGAGTAAAGATGCAGA------TGTAGTA------
TCTGATGACAAGGCTGCAGATAGTGTAACAGCATTTTCAGGATTGGTTAACGTTTATTACGATATAGCG---
ATTGAAGATATGCCTATCACTCCATACGTTGGTGTTGGTGTTGGTGCAGCATATATCAGCAATCCTTCAAAAGCTGATGAAGTTAAAGATCAA
AAA---
GGATTTGGTTTTGCTTATCAAGCAAAAGCTGGTGTTAGTTATGATGTAACCCCAGAAATCAAACTCTTTGCTGGAGCTCGTTACTTCGGTTCTT
ATGGTGCTAGTTTTGATAAGGCAGATAAGGATGAT
>Cacopsylla_fraudatrix_wMyr04

ACAAAGATTGATGGTGTTACATATAAATCAGGTAAGGAGAACA---
ATAGTCCCTTAAAAGCATCTTTTCTAGCTGGAGGTGGTGCATTTGGTTATAAAATGGATGATATCAGGGTTGATGTTGAAGGACTTTACTCAC
AATTGAGTAAAGATGCAGA------TGTAGTA------
TCTGATGACAAGGCTGCAGATAGTGTAACAGCATTTTCAGGATTGGTTAACGTTTATTACGATATAGCG---
ATTGAAGATATGCCTATCACTCCATACGTTGGTGTTGGTGTTGGTGCAGCATATATCAGCAATCCTTCAAAAGCTGATGCAGTTAAAGATCAA
AAA---
GGATTTGGTTTTGCTTATCAAGCAAAAGCTGGTGTTAGTTATGATGTAACCCCAGAAATCAAACTCTTTGCTGGAGCTCGTTACTTCGGTTCTT
ATGGTGCTAGTTTTGATAAGGCAGATAAGGATGAT
>Cacopsylla_ledi_wMyr03
ACAAAGATTGATGGTGTTACATATAAATCAGGTAAGGAGAACA---
ATAGTCCCTTAAAAGCATCTTTTCTAGCTGGAGGTGGTGCATTTGGTTATAAAATGGATGATATCAGGGTTGATGTTGAAGGACTTTACTCAC
AATTGAGTAAAGATGCAGA------TGTAGTA------
TCTGATGACAAGGCTGCAGATAGTGTAACAGCATTTTCAGGATTGGTTAACGTTTATTACGATATAGCG---
ATTGAAGATATGCCTATCACTCCATACGTTGGTGTTGGTGTTGGTGCAGCATATATCAGCAATCCTTCAAAAGCTGATGAAGTTAAAGATCAA
AAA---
GGATTTGGTTTTGCTTATCAAGCAAAAGCTGGTGTTAGTTATGATGTAACCCCAGAAATCAAACTCTTTGCTGGAGCTCGTTACTTCGGTTCTT
ATGGTGCTAGTTTTGATAAGGCAGCTAACGATGAT
>Cacopsylla_myrtilli_wMyr02
ACAAAGATTGATGGTGTTACATATAAATCAGGTAAGGAGAACA---
ATAGTCCCTTAAAAGCATCTTTTCTAGCTGGAGGTGGTGCATTTGGTTATAAAATGGATGATATCAGGGTTGATGTTGAAGGACTTTACTCAC
AATTGAGTAAAGATGCAGA------TGTAGTA------
TCTGATGACAAGGCTGCAGATAGTGTAACAGCATTTTCAGGATTGGTTAACGTTTATTACGATATAGCG---
ATTGAAGATATGCCTATCACTCCATACGTTGGTGTTGGTGTTGGTGCAGCATATATCAGCAATCCTTCAAAAGCTGATGAAGTTAAAGATCAA
AAA---
GGATTTGGTTTTGCTTATCAAGCAAAAGCTGGTGTTAGTTATGATGTAACCCCAGAAATCAAACTCTTTGCTGGAGCTCGTTACTTCGGTTCTT
ATGGTGCTAGTTTTGATAAGGCAGCTAGGGATGAT
>wCpyr2
ACAAGAATTGACGGCATTGAATATAAAAAAGGAGACGAAGTTC---
ATGATCCTTTAAAAGCATCTTTTATGGCTGGTGGTGCTGCATTTGGTTATAAAATGGACGATATCAGGGTTGATGTTGAGGGACTTTACTCAC
AACTAAACAAAAACGACGTTA---
GTGGTGCAACATTTACTCCAACAACTGTTGCAAACAGTGTGGCAGCATTTTCAGGATTGGTTAACGTTTATTACGATATAGCG---
ATTGAAGATATGCCTATCACTCCATACGTTGGTGTTGGTGTTGGTGCAGCATATATCAGCAATCCTTCAGAAGCTAGTGCAGTTAAAGATCAA
AAA---
GGATTTGGTCTTGCTTATCAAGCAAAAGCTGGTGTTAGTTATGATGTAACCCCAGAAATCAAGCTTTATGCTGGTGCTCGTTATTTTGGTTCTT
ATGGTGCTAGTTTTAATAAAGAAGCAGTATCAGCT
>wCpyr1
ACAAGAATTGACGGCATTGAATATAAAAAAGGAGACGAAGTTC---
ATGATCCTTTAAAAGCATCTTTTATGGCTGGTGGTGCTGCATTTGGTTATAAAATGGACGATATCAGGGTTGATGTTGAGGGACTTTACTCAC
AACTAAACAAAAACGACGTTA---
GTGGTGCAACATTTACTCCAACAACTGTTGCAAACAGTGTGGCAGCATTTTCAGGATTGGTTAACGTTTATTACGATATAGCG---
ATTGAAGATATGCCTATCACTCCATACGTTGGTGTTGGTGTTGGTGCAGCATATATCAGCAATCCTTCAGAAGCTAGTGCAGTTAAAGATCAA
AAA---

GGATTTGGTCTTGCTTATCAAGCAAAAGCTGGTGTTAGTTATGATGTAACCCCAGAAATCAAGCTTTATGCTGGTGCTCGTTATTTTGGTTCTT
ATGGTGCTAGTTTTAATAAAGAAGCAGTATCAGCT
>Drosophila_simulans_wNo
ACAAGAATTGACGGCATTGAATATAAAAAAGGAACCGAAGTTC---
ATGATCCTTTAAAAGCATCTTTTATGGCTGGTGGTGCTGCATTTGGTTATAAAATGGACGATATCAGGGTTGATGTTGAGGGACTTTACTCAC
AACTAAACAAAAACGACGTTA---
GTGGTGCAACATTTACTCCAACAACTGTTGCAAACAGTGTGGCAGCATTTTCAGGATTGGTTAACGTTTATTACGATATAGCG---
ATTGAAGATATGCCTATCACTCCATACGTTGGTGTTGGTGTTGGTGCAGCATATATCAGCAATCCTTCAGAAGCTAGTGCAGTTAAAGATCAA
AAA---
GAATTTGGTTTTGCTTATCAAGCAAAAGCTGGTGTTAGTTATGATGTAACCCCAGAAATCAAGCTTTATGCTGGTGCTCGTTATTTTGGTTCTT
ATGGTGCTAGTTTTAATAAAGAAGCAGTATCAGCT
>Drosophila_simulans_wMA
ACAAGAATTGACGGCATTGAATATAAAAAAGGAACCGAAGTTC---
ATGATCCTTTAAAAGCATCTTTTATGGCTGGTGGTGCTGCATTTGGTTATAAAATGGACGATATCAGGGTTGATGTTGAGGGACTTTACTCAC
AACTAAACAAAAACGACGTTA---
GTGGTGCAACATTTACTCCAACAACTGTTGCAAACAGTGTGGCAGCATTTTCAGGATTGGTTAACGTTTATTACGATATAGCG---
ATTGAAGATATGCCTATCACTCCATACGTTGGTGTTGGTGTTGGTGCAGCATATATCAGCAATCCTTCAGAAGCTAGTGCAGTTAAAGATCAA
AAA---
GAATTTGGTTTTGCTTATCAAGCAAAAGCTGGTGTTAGTTATGATGTAACCCCAGAAATCAAGCTTTATGCTGGTGCTCGTTATTTTGGTTCTT
ATGGTGCTAGTTTTAATAAAGAAGCAGTATCAGCT
>Culex_quinquefasciatus_wPip
ACAAGAATTGACGGCATTGAATATAAAAAAGGAACCGAAGTTC---
ATGATCCTTTAAAAGCATCTTTTATGGCTGGTGGTGCTGCATTTGGTTATAAAATGGACGATATCAGGGTTGATGTTGAGGGACTTTACTCAC
AACTAAACAAAAACGACGTTA---
GTGGTGCAACATTTACTCCAACAACTGTTGCAAACAGTGTGGCAGCATTTTCAGGATTGGTTAACGTTTATTACGATATAGCG---
ATTGAAGATATGCCTATCACTCCATACGTTGGTGTTGGTGTTGGTGCAGCATATATCAGCAATCCTTCAGAAGCTAGTGCAGTTAAAGATCAA
AAA---
GGATTTGGTTTTGCTTATCAAGCAAAAGCTGGTGTTAGTTATGATGTAACCCCAGAAATCAAACTCTTTGCTGGTGCTCGTTATTTTGGTTCTT
ATGGTGCTAGTTTTAATAAAGAAGCAGTATCAGCT
>Aedes_albopictus_wAlbB
ACAAGAATTGACGGCATTGAATATAAAAAAGGAACCGAAGTTC---
ATGATCCTTTAAAAGCATCTTTTATGGCTGGTGGTGCTGCATTTGGTTATAAAATGGACGATATCAGGGTTGATGTTGAGGGACTTTACTCAC
AACTAAACAAAAACGACGTTG---
GTGGTGCAACATTTGCTCCAACAACTGTTGCAAACAGTGTGGCAGTATTTTCAGGATTGGTTAACGTTTATTACGATATAGCG---
ATTGAAGATATGCCTATCACTCCATACGTTGGTGTTGGTGTTGGTGCAGCATATATCAGCAATCCTTCAGAAGCTAGTGCAGTTAAAGATCAA
AAA---
GGATTTGGTTTTGCTTATCAAGCAAAAGCTGGTGTTAGTTATGATGTAACCCCAGAAATCAAGCTTTATGCTGGTGCTCGTTATTTTGGTTCTT
ATGGTGCTAGTTTTAATAAAGAAACAGTATCAGCT
>Agonoscena_pegani
---------------------------------ACCGAATTTC---
ATGATCCTTTAAAAGCATCTTTTATGGCTGGTAGTGCTGCATTTGGTTATAAAATGGACGATATCAGGGTTGATGTTGAGGGACTTTACTCAC
AACTAAACAAAAACGACGTTA---
GTGGTGCAACATTTACTCCAACAACTGTTGCAAACAGTGTGGCAGCATTTTCAGGATTGGTTAACGTTTATTACGATATAGCG---

ATTGAAGATATGCCTATCACTCCATACGTTGGTGTTGGTGTTGGTGCAGCATATATCAGCAATCCTTCAGAAGCTAGTGCAGTTAAAGATCAA
AAA---
GGATTTGGTTTTGCTTATCAAGCAAAAGCTGGTGTTAGTTATGATGTAACCCCAGAAATCAAACTCTTTGCTGGTGCTCGTTATTTTGGTTCTT
ATGGTGCTAGT------------------------
>Agonoscena_bimaculata
---------------ATTACAAATGCAAGAGGTAAAGAAAATC---
ATGATCCTTTAAAAGCATCTTTTATGGCTGGTGGTGCTGCATTTGGTTATAAAATGGACGATATCAGGGTTGATGTTGAGGGACTTTACTCAC
AACTAAACAAAAACGACGTTA---
GTGGTGCAACATTTACTCCAACAACTGTTGCAAACAGTGTGGCAGCATTTTCAGGATTGGTTAACGTTTATTACGATATAGCG---
ATTGAAGATATGCCTATCACTCCATACGTTGGTGTTGGTGTTGGTGCAGCATATATCAGCAATCCTTCAGAAGCTGATGCAGTTAAAGATCAA
AAA---
GGATTTGGTTTTGCTTATCAAGCAAAAGCTGGTGTGAGTTATGATGTAACTCCAGAAATCAAACTCTTTGCTGGAGCTCGTTACTTCGGTTCTT
ATGGTGCTAGT------------------------
>Bactericera_cockerelli 1
ACAAGGATTGATGGTGTTAAATATAAATCAGGTAAGGACAACA---
ATAGTCCCTTAAAAGCATCTTTTCTCGCTGGAGGTGGTGCATTTGGTTATAAAATGGATGATATCAGGGTTGATGTTGAAGGACTTTACTCAC
AATTGAGTAAAGATGCAGA------TGTAGTAGATACTTCTCCAGAA---
GTTGTAGAAAGTTTAACAGCATTTTCAGGACTAGTTAATGTTTATTACGATATAGCA---
ATTGAAGATATGCCTATCACTCCATACGTTGGTGTTGGTGTTGGTGCAGCGTATGTAAGCAATCCTTTAGTAACAGAGGTTACTGGTGATAAA
AAATCTGGATTTGGTTTTGCTTATCAAGCAAAAGCTGGTGTTAGTTATGATGTAACCCCAGAAATCAAGCTTTATGCTGGTGCTCGTTATTTTG
GTTCTTATGGTGCTAATTTTGGTAAGGCAGCTAAAGATGAT
>wSaph
ACAAAAGTTGATGGTATTACCTATAAGAAAGACAAGAGTGATT---
ACAGTCCATTAAAACCATCTTTTATAGCTGGTGGTGGTGCATTTGGTTACAAAATGGACGACATCAGGGTTGATGTTGAAGGAGTTTATTCAT
ACCTAAACAAAAATSATGTTAAAGGTGTAACATTTGACCCAGCAAATACTATTGCAGACAGTGTAACAGCAATTTCAGGATTAGTGAACGTG
TATTACGATATAGCA---
ATTGAAGATATGCCTATCACTCCATACATTGGTGTTGGTGTTGGTGCAGCGTATATTAGCACTCCTTTGGAACCCGCTGTGAATGATCAAAAA
AGT---
AAATTTGGTTTTGCTGGTCAAGTAAAAGCTGGTGTTAGYTATGATGTAACTCCAGAARTCAAACTTTATGCTGGAGCTCGTTATTTCGGTTCTT
ATGGTGCTAATTTTGATGGAAAAAAAACAGATCCT
>Drosophila_melanogaster_wMel
ACAAAAGTTGATGGTATTACCTATAAGAAAGACAAGAGTGATT---
ACAGTCCATTAAAACCATCTTTTATAGCTGGTGGTGGTGCATTTGGTTACAAAATGGACGACATCAGGGTTGATGTTGAAGGAGTTTATTCAT
ACCTAAACAAAAATGATGTTAAAGATGTAACATTTGACCCAGCAAATACTATTGCAGACAGTGTAACAGCAATTTCAGGATTAGTGAACGTG
TATTACGATATAGCA---
ATTGAAGATATGCCTATCACTCCATACATTGGTGTTGGTGTTGGTGCAGCGTATATTAGCACTCCTTTGGAACCCGCTGTGAATGATCAAAAA
AGT---
AAATTTGGTTTTGCTGGTCAAGTAAAAGCTGGTGTTAGTTATGATGTAACTCCAGAAGTCAAACTTTATGCTGGAGCTCGTTATTTCGGTTCTT
ATGGTGCTAATTTTGATGGAAAAAAAACAGATCCT
>Xylosandrus_germanus_wXge1
ACAAAAGTTGATGGTATTACCTATAAGAAAGACAAGAGTGATT---
ACAGTCCATTAAAACCATCTTTTATAGCTGGTGGTGGTGCATTTGGTTACAAAATGGACGACATCAGGGTTGATGTTGAAGGAGTTTATTCAT
ACCTAAACAAAAATGATGTTAAAGATGTAACATTTGACCCAGCAAATACTATTGCAGACAGTGTAACAGCAATTTCAGGATTAGTGAACGTG

TATTACGATATAGCA---
ATTGAAGATATGCCTATCACTCCATACATTGGTGTTGGTGTTGGTGCAGCGTATATTAGCACTCCTTTGGAACCCGCTGTGAATGATCAAAAA
AGT---
AAATTTGGTTTTGCTGGTCAAGTAAAAGCTGGTGTTAGTTATGATGTAACTCCAGAAGTCAAACTTTATGCTGGAGCTCGTTATTTCGGTTCTT
ATGGTGCTAATTTTGATGGAAAAAAAACAGATCCT
>Euwallacea_interjectus_wEi
ACAAAAGTTGATGGTATTACCTATAAGAAAGACAAGAGTGATT---
ACAGTCCATTAAAACCATCTTTTATAGCTGGTGGTGGTGCATTTGGTTACAAAATGGACGACATCAGGGTTGATGTTGAAGGAGTTTATTCAT
ACCTAAACAAAAATGATGTTAAAGATGTAACATTTGACCCAGCAAATACTATTGCAGACAGTGTAACAGCAATTTCAGGATTAGTGAACGTG
TATTACGATATAGCA---
ATTGAAGATATGCCTATCACTCCATACATTGGTGTTGGTGTTGGTGCAGCGTATATTAGCACTCCTTTGGAACCCGCTGTGAATGATCAAAAA
AGT---
AAATTTGGTTTTGCTGGTCAAGTAAAAGCTGGTGTTAGTTATGATGTAACTCCAGAAGTCAAACTTTATGCTGGAGCTCGTTATTTCGGTTCTT
ATGGTGCTAATTTTGATGGAAAAAAAACAGATCCT
>Psyttalia_carinata_wRha
ACAAAAGTTGATGGTATTACCTATAAGAAAGACAAGAGTGATT---
ACAGTCCATTAAAACCATCTTTTATAGCTGGTGGTGGTGCATTTGGTTACAAAATGGACGACATCAGGGTTGATGTTGAAGGAGTTTATTCAT
ACCTAAACAAAAATGATGTTAAAGATGTAACATTTGACCCAGCAAATACTATTGCAGACAGTGTAACAGCAATTTCAGGATTAGTGAACGTG
TATTACGATATAGCA---
ATTGAAGATATGCCTATCACTCCATACATTGGTGTTGGTGTTGGTGCAGCGTATATTAGCACTCCTTTGGAACCCGCTGTGAATGATCAAAAA
AGT---
AAATTTGGTTTTGCTGGTCAAGTAAAAGCTGGTGTTAGTTATGATGTAACTCCAGAAGTCAAACTTTATGCTGGAGCTCGTTATTTCGGTTCTT
ATGGTGCTAATTTTGATGGAAAAAAAACAGATCCT
>Anastrepha_fraterculus_wAfraCast2A
ACAAAAGTTGATGGTATTACCTATAAGAAAGACAAGAGTGATT---
ACAGTCCATTAAAACCATCTTTTATAGCTGGTGGTGGTGCATTTGGTTACAAAATGGACGACATCAGGGTTGATGTTGAAGGAGTTTATTCAT
ACCTAAACAAAAATGATGTTAAAGATGTAACATTTGACCCAGCAAATACTATTGCAGACAGTGTAACAGCAATTTCAGGATTAGTGAACGTG
TATTACGATATAGCA---
ATTGAAGATATGCCTATCACTCCATACATTGGTGTTGGTGTTGGTGCAGCGTATATTAGCACTCCTTTGGAACCCGCTGTGAATGATCAAAAA
AGT---
AAATTTGGTTTTGCTGGTCAAGTAAAAGCTGGTGTTAGTTATGATGTAACTCCAGAAGTCAAACTTTATGCTGGAGCTCGTTATTTCGGTTCTT
ATGGTGCTAATTTTGATGGAAAAAAAACAGATCCT
>Xyleborus_seiryorensis_wXse
ACAAAAGTTGATGGTATTACCTATAAGAAAGACAAGAGTGATT---
ACAGTCCATTAAAACCATCTTTTATAGCTGGTGGTGGTGCATTTGGTTACAAAATGGACGACATCAGGGTTGATGTTGAAGGAGTTTATTCAT
ACCTAAACAAAAATGATGTTAAAGATGTAACATTTGACCCAGCAAATACTATTGCAGACAGTGTAACAGCAATTTCAGGATTAGTGAACGTG
TATTACGATATAGCA---
ATTGAAGATATGCCTATCACTCCATACATTGGTGTTGGTGTTGGTGCAGCGTATATTAGCACTCCTTTGGAACCAGCTGTGAATGATCAAAAA
AGT---
AAATTTGGTTTTGCTGGTCAAGTAAAAGCTGGTGTTAGTTATGATGTAACTCCAGAAGTCAAACTTTATGCTGGAGCTCGTTATTTCGGTTCTT
ATGGTGCTAATTTTGATGGAAAAAAAACAGATCCT
>Drosophila_bifasciata_wBif

ACAAAAGTTGATGGTATTACCTATAAGAAAGACAATAGTGATT---
ACAGTCCATTAAAAGCGTCTTTTATAGCTGGTGGTGGTGCGTTTGGTTACAAAATGGACGACATCAGGGTTGATGTTGAAGGAGTTTATTCAT
ACCTAAACAAAAATGATGTTACAGATGCAAAATTTA---
CGCCAGATACTATTGCAGACAGTTTAACAGCAATTTCAGGACTAGTTAACGTTTATTACGATATAGCA---
ATTGAAGATATGCCTATCACTCCATATATTGGTGTTGGTGTTGGTGCAGCGTATATTAGCACTCCTTTGAAAGACGCTGTGAATGATCAAAAA
AGT---
AAATTTGGTTTTGCTGGTCAAGTAAAAGCTGGTGTTAGTTATGATGTAACTCCGGAAGTCAAACTTTATGCTGGAGCTCGTTATTTCGGTTCTT
TTGGTGCTCATTTTGATAAAGATGCTGCTGCAGGC
>A_PS122
ACAAAAGTTGATGGTATTACCTATAAGAAAGACAATAGTGATT---
ACAGTCCATTAAAAGCGTCTTTTATAGCTGGTGGTGGTGCGTTTGGTTACAAAATGGACGACATCAGGGTTGATGTTGAAGGAGTTTATTCAT
ACCTAAACAAAAATGATGTTACAGATGCACAATTTA---
CGCCAGATACTATTGCAGACAGTTTAACAGCAATTTCAGGACTAGTTAACGTTTATTACGATATAGCA---
ATTGAAGATATGCCTATCACTCCATATATTGGTGTTGGTGTTGGTGCAGCGCATATTAGCACTCCTTTGAAAGACGCTGTGAATGATCAAAAA
AGT---
AAATTTGGTTTTGCTGGTCAAGTAAAAGCTGGTGTTAGTTATGATGTAACTCCGGAAGTCAAACTTTATGCTGGAGCTCGTTATTTCGGTTCTT
TTGGTGCTCATTTTGATAAAGATGCTGCTGCAGGC
>Folsomia_candida_wFcan
ACAAAGATTGATACATTTACAGCTACAAAAGATAGCGATACCACTCTTGATCCTTTTAAGGCTTCTTTCATAGGTGGTGGAGCCGAATTTGGT
TACAAAATGGAGGACTTCAGAGTTGGAGTTGAAGGGGTTTACTCACAATTGAATAAGAATGCTGACACAGGTGTAGTGCTTGCACCTGCAGA
TGCAGCTGCAGAAAAATTAACAGCAATTGCAGTATTAGTTAATGTGAATTACGATATAGCAATTATTGAAGATCTGCCTGTAGCTCCATACGT
TGGTGTGGGTGTTGGTGCAGCATATATCAACAATCCTTTAAAAGCTCCTTTTAATGAGCAAAAAAGT---
GGATTCGGTGTTGCTTATCAAGCAAAAGCTGGTGTTAGTTATGCTGTAACCCCAGAAATTAATCTCTATGCTGGTGCTCGCTACTTCGGCTCTT
ATGGTGCTAAATTTGATAAAAGTGGTGAGGAAGGT

Supplementary File 3. Concatenated alignment of the MLST genes used for the phylogenetic analysis (**Figs 2B**, S2B).
>wCpyr3
AAGATGCCACTGTTTGTTTGGTCTGTCTTGTTAACAGCATTTATGTTGATTGTTGCCTTACCAGTGCTTGCCGGTGCTATAACTATGCTTCTTAC
TGATCGCAATATTGGTACTTCCTTTTTGATCCTGCCGGTGGTGGTGACCCTGTGTTATTTCAACATTTATTTTGGTTTTTGGTCATCCGGAGG
TTTACGTAATTATTTTTCCTGCATTTGGCATCATAAGTCAGGTTGTATCAACTTTTTCTCACAGACCTGTATTTGGTTACATAGGAATGGTTTAT
GCAATGATAGGTATAGCAGTATTTGGCTTTATGGTTTGGGCTCACCATATGTTCACTGTTGGGCTTAGTGCTGACGCTGCTGCATTTTTTCTTA
TTTTGAAGCTTAATAGCGCTAACTCTTTGCACTCAAAAAGCTTAACTTCAGATCAAGCAATAACTGCCTCTGTAAAAGATGCGCTGCGTTTGG
GCTGCATGGCTGTTGGGTTTACTATATATCCTGGTTCTGCTAAGTGTTTTGATATGATGGAAGAAGCTCGCAAAATTATAGCTGAGGCTAAAT
CTTGTGGCCTTGCTGTAGTGCTATGGTCTTATCCACGTGGTGAAGGGATTTCCAAAGAAGGTGAAACAGCAGTTGATGTGATTGCTTATGCTG
CGCATATAGCGGCTTTGCTCGGTGCCAACATAATAAAAGTAAAACTTCCAACCAATCATCTGGAAAGAGAAAAAATAGAAAnnnnnnnnnnnnn
nnnnnnnnnnnnnnnnnnnnnnnnnnnnnnnnnnnnnnnnnnnnnnnnnnnnnnnnnnnnnnnnnnnnnnnnnnnnnnnnnnnnnnnnnnnnnnnnnnnnnnnnnnnnnnnnnnnnnnnnnn
nnnnnnnnnnnnnnnnnnnnnnnnnnnnnnnnnnnnnnnnnnnnnnnnnnnnnnnnnnnnnnnnnnnnnnnnnnnnnnnnnnnnnnnnnnnnnnnnnnnnnnnnnnnnnnnnnnnnnnnnn
nnnnnnnnnnnnnnnnnnnnnnnnnnnnnnnnnnnnnnnnnnnnnnnnnnnnnnnnnnnnnnnnnnnnnnnnnnnnnnnnnnnnnnnnnnnnnnnnnnnnnnnnnnnnnnnnnnnnnnnn
nnnnnnnnnnnnnnnnnnnnnnnnnnnnnnnnnnnnnnnnnnnnnnnnnnnnnnnnnnnnnnnnnnnnCGTTACATCGGTTCATGTGATGGTGATATGGAAAAGGGGTC
ACTTCGCTGTGATGCAAATGTTTCTGTTCGCCCAAAGGGCAGTAGCACATTTGGCACTCGTTGTGAAATAAAAAACTTAAATTCAATACGTTA
TATTGTACAAGCTATAGATTATGAAGCACAAAGGCAGATCAAAATTTTGGAAAGCGGAGGAGAAATAAGTCAAGATACCTTATTGTTTGATG
TCACTTTAGGAAAAACAAAAGTGATGAGAAGCAAAGAAGATTCAAGTGACTATAGATATTTCCCTGAACCTGATTTGCTACCTGTTGAAATA
AGCCAAGACAAACCACGTCTTCGCTCTGCTATCTTTGCTGCGCGAAAGGAAAATCTACCAAAAGATAAAATAGAAACAGCAATAAAAAATGC
AGCTGGTAACGTTGCTGGAGAAAGTTATGAAGAAATACAATATGAAGGCTGCGGACCTTCTGGTGCTGCACTTATTGTCCATGCTCTGACAAA
TAATCGCAACCGAACTGCTTCTGAGATACGTTATATCTTTTCTCGCAAAGGCGGTAATTTGGGAGAAACAGGATGTGTGAGTTACCTTTTCGA
TCATGTAGGCTTAATTGTCTATAAAGCAGAGGGTATAAGTTTTGAAGATTTATTTAACTATGGAATTGAATTAGAAGTATTGAATGTTGAGGA
AAATAACAAAGAAGAATTATATGTTATAACTTGTGAAGTAAAAGACTTTGGTAAAGTACGTGACGCTTTC
>wCpyr1
AAGATACCACTATTTGTTTGGTCTATTTTACTCACGTCATTTATGTTAATTGTTGCCTTACCGGTACTTGCTGGTGCTATAACTATGCTGCTCAC
TGATCGTAATATAGGTACCTCCTTTTTGATCCTGCTGGTGGTGGTGATCCTGTGTTATTTCAACATCTGTTTTGGTTTTTGGTCACCCAGAGG
TTTATATCATTATTTTTCCTGCGTTTGGCATTATAAGCCAAGTCGTGTCAACTTTTTCCCATAGACCAGTATTTGGCTATAAGGGAATGGTTTAT
GCAATGATAGGTATAGCAGCATTTGGTTTTATGGTTTGGGCTCACCATATGTTTACTGTTGGGCTTAGCGAAGATGCTGCTGTGTTTTTTCTTA
TTTTGAAGCTTAATAGTGCCAACTCCTTACACTCAAAAAGCTTAACTTCTGATCAAGCAATAACTGCTTCTGTAAAAGATGCACTGCGTTTAG
GCTGTGCGGCTGTTGGGTTTACTATATATCCTGGTTCTGCTAAATGTTTTGATATGATGGAAGAAGCTCGCGAAATTATAGCTGAGGCTAAGT
CCTTCGGACTTGCTGTGGTGCTATGGTCTTATCCACGTGGTGAAGGGATTTCCAAAGAAGGTGAAACAGCAGTTGATGTAATTGCTTATGCTG
CGCATATAGCGGCTTTGCTCGGTGCCAACATAATAAAGGTAAAACTTCCAACCAACCATCTGGAAAGAGAAAAAATAGAAGAACAGGTGCT
GCACCGGTAATTGCAAAGGCAGCCAAAGAAGCWWKAGCTGCAGTTAAAGATAAAGGAGCAAAAGAAAAAAAGATACTGACTGTTGGAGTT
GTAACCAAGCCGTTCGGTTTTGAAGGTGTGCGACGTATGCGCATTGCAGAGCTTGGACTTGAAGAGTTGCAAAAGCACGTAGATACACTTATT
GTTATTCCCAATCAAAACTTGTTTAGAATTGCTAATGAGAAAACTACATTTGCTGATGCATTTCAACTCGCCGATAATGTTCTACATATTGGCA
TAAGAGGAGTAACTGATTTGATGATCATGCCAGGACTTATTAATCTTGATTTTGCTGACATAGAAACAGTAATGAGTGAGATGGGTAAAGCA
ATGATTGGTACTGGAGAGGCAGAAGGAGAAGATAGGGCAATTAGTGAAGCTGCAGAATTCATGAAAAAATTGAGGCAGATTTTGCGTTACA
TCGGTTCATGTGATGGTGATATGGAAAAGGGGTCACTTCGCTGTGATGCAAATGTTTCTGTTCGCCCAAAGGGTAGTAGCACATTTGGCACTC
GTTGTGAAATAAAAAACTTAAATTCAATACGTTATATTGTACAAGCTATAGATTATGAAGCACAAAGGCAGATCAAAATTTTGGAAAGCGGA
GGAGAAATAAGTCAAGATACCTTATTGTTTGATGTCACTTTAGGAAAAACAAAAGTGATGAGAAGCAAAGAAGATTCAAGTGACTATAGATA
TTTCCCTGAACCTGATTTGCTACCTGTTGAAATAAGCCAAGACAAACCACGTCTTCGCTCTGCTATCTTTGCTGCGCGAAAGGAAAATCTACC
AAAAGATAAAATAGAAACAGCAATAAAAAATGCAGCTGGTAACGTTGCTGGAGAAAGTTATGAAGAAATACAATATGAAGGCTGCGGACCT
TCTGGTGCTGCACTTATTGTCCATGCTCTGACAAATAATCGCAACCGAACTGCTTCTGAGATACGTTATATCTTTTCTCGCAAAGGCGGTAATT
TGGGAGAAACAGGATGTGTGAGTTACCTTTTCGATCATGTAGGCTTAATTGTCTATAAAGCAGAGGGTATAAATTTTGAAGATTTATTTAACT

ATGGAATTGAATTAGAAGTATTGAATGTTGAGGAAAATAACAAAGAAGAATTATATGTTATAACTTGTGAAGTAAAAGACTTTGGTAAAGTA
CGTGACGCTTTC
>wSaph
AAGATGCCACTRTTTRTTTGGTCTGTCTTGCTAACAGCATTTATGTTGATTGTCGCCTTACCAGTGCTTGCCGGTGCTATAACTATGCTTCTTAC
TGATCGCAATGTTGGTACTTCCTTTTTTGATCCTGCAGGTGGTGGTGACCCTGTGTTATTTCAACATTTATTTTGGTTTTTTGGTCATCCAGAAG
TTTACGTAATTATTTTTCCTGCATTTGGCATCATAAGTCAGGTTGTATCAACTTTTTCTCACAGACCTGTATTTGGTTACATAGGGATGGTTTAT
GCAATGATAGGTATAGCAGTATTTGGCTTTATGGTTTGGGCTCATCATATGTTCACTGTTGGGCTTAGTGCTGACGCTGCTGCATTTTTTCTTAT
TTTGAAACTTAATAGTTCCAACTCCTTACATTCAAAGGATCTGACCTCTGATCAAGCAATAACCTCTTCTGTGAAAGATGCACTGCGTTTGGGC
TGCTTGGCTGTTGGATTTACTATATATCCTGGTTCTGCTAAGTGTTTCGATATGATGGAAGAAGCCCGTGGAATCATAGCTGAAGCCAAATCTT
ACGGGCTTGCAGTAGTGCTATGGTCTTATCCACGCGGTGAAGGAATTTCCAAAGAAGGTGAAACAGCGGTTGATGTTATTGCCTATGCTGCAC
ACATGGCAGCTTTGCTTGGCGCTAATATAATAAAAGTAAAACTTCCAACTAAATATTTGGAAAGGGAGAAAATAGAAAAACCGGTGCTGCAC
CGGTAATTGCAAAAGCAGCCAGAGAAGCAAGAGCCGCAGTTAAGGATAGAGCGCCAAAAGAAAAAAAGATATTGACTGTTGGAGTTGTAAC
TAAACCGTTCGGTTTTGAAGGTGTGCGCCGTATGCGCATTGCAGAGCTTGGACTTGAAGAACTGCAAAAATACGTGGATACACTTATTGTCAT
TCCAAATCAGAATTTATTTAGAATTGCAAATGAAAAAACTACATTTTCTGATGCATTTAAACTTGCTGAYAATGTTCTGCATATTGGCATCAG
AGGAGTAACTGACTTGATGGTCATGCCAGGGCTTATTAATCTTGACTTCGCTGATATAGAAACAGTAATGAGCGAGATGGGCAAAGCGATGA
TTGGCACCGGAGAGGCAGAAGGAGAAGATAGAGCAATTAGTGAAGCTGCAGAATGCATGAAAAGATTGAGGCAGATTTTGCGTTATATTGG
TTCGTGTGATGGTGATATGGAAAAGGGATCACTTCGTTGTGATGCAAATGTTTCTGTTCGCCTAAAAGGCAGTAGTACATTTGGCACTCGTTG
TGAAATAAAAAATCTGAACTCGATACGTTATATTGTGCAAGCTATAGACTATGAAATACAAAGACAAATTGAAATTTTAGAAAGTGGAAAAG
AAATAAGTCAAGATACCTTATTGTTTGATGTTGCTTTGGGAAAAACAAAAGTGATGAGAAGCAAAGAGGATGCAAGCGACTATAGATACTTC
CCTGAGCCTGATTTATTACCTGTTGAGGTAAGCCAGGAGAAACCGCGCCTTCGCTCTGCTATATTTGCTGCACGCAAGGAAAATCTACCAAAA
GATAAAATAGAAACAGCAATAAAAAATGCAACTGGTAACGTTGCTGGAGAAAATTACGAGGAAATACAATATGAAGGTCATGGGCCTTCTG
GCACTGCACTCATTGTCCATGCCTTGACTAATAACCGCAACCGTACTGCTTCTGAGGTACGTTATATCTTTTCTCGTAAAGGTGGAAATTTAGG
AGAAACAGGAAGTGTTAGTTACCTTTTCGATCATGTAGGCTTAATCGTCTATAAAGCAGAGGGTGTGAATTTTGATGATTTATTCAGTCATGG
AATCGAATTAGAAGTATTGAATGTTGAGGAAAATGACAAAGAAGGATTACACGTTATAACTTGTGAAATAAAAGATTTGGTAAAGTACGCG
ATGCCTTT
>wCpyr2
AAGATACCACTATTTGTTTGGTCTATTTTACTCACGTCATTTATGTTAATTGTTGCCTTACCGGTACTTGCTGGTGCTATAACTATGCTGCTCAC
TGATCGTAATATAGGTACCTCCTTTTTTGATCCTGCTGGTGGTGGTGATCCTGTGTTATTTCAACATCTGTTTTGGTTTTTTGGTCACCCAGAGG
TTTATATCATTATTTTTCCTGCGTTTGGCATTATAAGCCAAGTCGTGTCAACTTTTTCCCATAGACCAGTATTTGGCTATAAGGGAATGGTTTAT
GCAATGATAGGTATAGCAGCATTTGGTTTTATGGTTTGGGCTCACCATATGTTTACTGTTGGGCTTAGCGAAGATGCTGCTGTGTTTTTTCTTA
TTTTGAAGCTTAATAGTGCCAACTCCTTACACTCAAAAAGCTTAACTTCTGATCAAGCAATAACTGCTTCTGTAAAAGATGCACTGCGTTTAG
GCTGTGCGGCTGTTGGGTTTACTATATATCCTGGTTCTGCTAAATGTTTTGATATGATGGAAGAAGCTCGCGAAATTATAGCTGAGGCTAAGT
CCTTCGGACTTGCTGTGGTGCTATGGTCTTATCCACGTGGTGAAGGGATTTCCAAAGAAGGTGAAACAGCAGTTGATGTAATTGCTTATGCTG
CGCATATAGCGGCTTTGCTCGGTGCCAACATAATAAAAGTAAAACTTCCAACCAACCATCTGGAAAGAGAAAAAATAGAAGAACAGGTGCT
GCACCGGTAATTGCAAAGGCAGCCAAAGAAGCWWKAGCTGCAGTTAAAGATAAAGGAGCAAAAGAAAAAAAGATACTGACTGTTGGAGTT
GTAACCAAGCCGTTCGGTTTTGAAGGTGTGCGACGTATGCGCATTGCAGAGCTTGGACTTGAAGAGTTGCAAAAGCACGTAGATACACTTATT
GTTATTCCCAATCAAAACTTGTTTAGAATTGCTAATGAGAAAACTACATTTGCTGATGCATTTCAACTCGCCGATAATGTTCTACATATTGGCA
TAAGAGGAGTAACTGATTTGATGATCATGCCAGGACTTATTAATCTTGATTTTGCTGACATAGAAACAGTAATGAGTGAGATGGGTAAAGCA
ATGATTGGTACTGGAGAGGCAGAAGGAGAAGATAGGGCAATTAGTGAAGCTGCAGAATTCATGAAAAAATTGAGGCAGATTTTGCGTTACA
TCGGTTCATGTGATGGTGATATGGAAAAGGGGTCACTTCGCTGTGATGCAAATGTTTCTGTTCGCCCAAAGGGTAGTAGCACATTTGGCACTC
GTTGTGAAATAAAAAACTTAAATTCAATACGTTATATTGTACAAGCTATAGATTATGAAGCACAAAGGCAGATCAAAATTTTGGAAAGCGGA
GGAGAAATAAGTCAAGATACCTTATTGTTTGATGTCACTTTAGGAAAAACAAAAGTGATGAGAAGCAAAGAAGATTCAAGTGACTATAGATA
TTTCCCTGAACCTGATTTGCTACCTGTTGAAATAAGCCAAGACAAACCACGTCTTCGCTCTGCTATCTTTGCTGCGCGAAAGGAAAATCTACC

AAAAGATAAAATAGAAACAGCAATAAAAAATGCAGCTGGTAACGTTGCTGGAGAAAGTTATGAAGAAATACAATATGAAGGCTGCGGACCT
TCTGGTGCTGCACTTATTGTCCATGCTCTGACAAATAATCGCAACCGAACTGCTTCTGAGATACGTTATATCTTTTCTCGCAAAGGCGGTAATT
TGGGAGAAACAGGATGTGTGAGTTACCTTTTCGATCATGTAGGCTTAATTGTCTATAAAGCAGAGGGTATAAATTTTGAAGATTTATTTAACT
ATGGAATTGAATTAGAAGTATTGAATGTTGAGGAAAATAACAAAGAAGAATTATATGTTATAACTTGTGAAGTAAAAGACTTTGGTAAAGTA
CGTGACGCTTTC
>Folsomia_candida_wFcan
AAAATGCCTTTGTTTGTCTGGTCTGTCCTGCTCACAGCATTTATGTTGATTGTTGCGTTACCTGTACTTGCTGGCGCGATAACTATGCTTCTTAC
TGATCGAAATATCGGCACTGCCTTTTTTGACCCTGCAGGTGGTGGAGATCCTGTATTATTTCAACATCTATTTTGGTTTTTCGGCCATCCAGAA
GTGTACGTCATCATTTTTCCTGCATTTGGTATTATAAGCCAGGTGGTATCAACTTTTTCTCATAAGCCAGTGTTTGGCTACACGGGGATGGTTT
ATGCAATGATAGGTATAGCTGCATTTGGCTTTATGGTTTGGGCTCATCATATGTTCACTGTTGGGCTTAGCGCTGATGCTGCTATATTTTTTCTT
ATTTTAAAGCTTAATAGCTCCAACTCTTTACACTCAAAAAATCTAACTTCTGACCAAGCAATAACCGCTTCTGTGAAGGATGCGCTCCGCTTG
GGGTGCGTAGCTGTTGGATTTACTATATATCCTGGTTCTGCTAAGTGTTTTGATATGATGGAAGAAGCTCGTGACATTATAGCTGAAGCCAAG
TCTTATGGACTTGCAGTAGTGCTATGGTCTTATCCACGTGGCGAGAGAATTTCGAAAGAAGGCGAAACTGCGGTTGATGTTATTGCTTATGCT
GCACATATAGCAGCTTTGCTTGGTGCTAATATCATAAAAGTAAAACTTCCAACTCAGCATTTGGAAAAAGAAAAGATAGACTAACTGGTGCT
GCACCGGTAATTGCAAAAGCAGCAAGAGAAGCAAGGGCTGCAGTTAAGGATAGGGCGCCAAAAGAAAAAAAGATATTGACTGTTGGAGTTG
TAACCAAACCGTTTGGCTTTGAAGGTGTGCGTCGTATGCGCATTGCAGAGCTGGGCCTTGAAGAATTACAAAAATATGTAGATACGCTCATTG
TTATTCCAAACCAAAATTTATTTAGAATTGCAAATGACAAAACAACATTTTCTGATGCGTTTAAGCTTGCCGATAATGTGCTTCATATTGGTAT
CAGAGGAGTAACTGACTTAATGGTCATGCCAGGGCTCATTAATCTTGATTTTGCTGACATAGAAACAGTAATGAGTGAAATGGGGAAAGCGA
TGATAGGAACTGGAGAGGCAACAGGAGAAGATAGGGCAATTAGTGAAGCTGCAGAATTTATGAAAAAATTGAGGCAGATTTTACGCTATAT
TGGCTCGTGTGACGGTGATATGGAAAAAGGGTCACTTCGCTGTGATGCAAATGTATCTATTCGTCCAAAAGGCAGTGACGCATTTGGAACTCG
TTGTGAGATAAAAAATTTAAATTCAATACGTTATGTTGTACAAGCCATAGATTATGAAATACAAAGACAAATTGAAATTTTAGAAAATGGAG
GAGAAATAAGTCAAGATACCTTATTGTTCGACGTTGCTTTAGGAAAAACAAAGGTGATGCGAAATAAAGAAGATGCAAGTGACTATAGATAC
TTTCCTGAACCTGATCTGTTGCCTGTTGAGGTCAGTCAAGAAAAGCCACGCCTTCGCTCTGCTATATTTGCTGCACGTAAAGAAAATTTACCA
AAAGATAAAATAGAGTTAGCAATAAAGAATGCAGCTGGTAATATTGCAGGAGAAAATTACGAGGAAATACAATATGAAGGCCACGGACCTT
TTGCTACTTCATTTATTGTTCATATTCTGACAAACAATCGCAATCGCACTGCTTCTGAGGTGCGTTATATCTTTTCTCGCAAAGGTGGAAATTT
AGGAGAAACAGGAAGCGTTAGTTATCTTTTTGATCATGTCGGCTTGATCGTTTATAAAGCAGAAGGCACAAATTTTGAGGATCTATTTAATCA
TGGAATCGAATTAGAGGTGCTCAATGTTGAGGAAAATAATGCAGAAGAAGTATATGTTATAACTTGCGCGGTTAAAGATTTTGGTAAAGTAC
GTGATGCCTTT
>Drosophila_simulans_wNo
AAAATGCCACTGTTTGTTTGGTCTGTTCTATTAACATCGTTTATGTTAATTGTAGCTTTACCGGTACTTGCTGGTGCTATAACTATGCTGCTCAC
TGATCGCAATATTGGCACTTCCTTTTTTGATCCTGCTGGTGGTGGTGATCCTGTGTTATTTCAACATCTGTTTTGGTTTTTTGGTCACCCAGAGG
TTTATATCATTATTTTTCCTGCGTTTGGCATTATAAGCCAAGTCGTGTCAACTTTTTCCCATAGACCAGTATTTGGCTATAAGGGAATGGTTTAT
GCAATGATAGGTATAGCAGCATTTGGTTTTATGGTTTGGGCTCACCATATGTTTACTGTTGGGCTTAGCGAAGATGCTGCTGTGTTTTTTCTTA
TTTTGAAGCTTAATAGCGCTAACTCTTTGCACTCAAAAAGCTTAACTTTAGATCAAGCAATAACTTCCTCTGTAAAAGATGCTCTACGTTTGGG
CTGCGTGGCTGTTGGGTTTACTATATATCCTGGTTCTGCTAAGTGTTTTGATATGATGGAAGAAGCTCGCAAAATTATAGCTGAGGCTAAATCT
TGTGGCCTTGCTGTAGTGCTATGGTCTTATCCACGTGGTGAAGGGATTTCAAAAGAAGGTGAAACAGCAGTTGATGTCATTGCTTATGCTGCG
CATATAGGGGCTTTGCTTGGTGCCAACATAATAAAAGTAAAACTTCCAACTAACCACTTGGAAAAAGAAAAAATAGAAAAACAGGCGCTGC
ACCGGTAATTGCAAAAGCAGCCAGAGAAGCAAGAGCGGTAGTTAAAGATAAAGGAGCAAAAGAAAAAAAGATACTGACTGTTGGAGTTGTA
ACTAAGCCGTTCGGTTTTGAAGGTGTGCGACGTATGCGCATTGCAGAGCTTGGACTTGAAGAGTTGCAAAAATACGTAGATACACTTATTGTC
ATTCCTAATCAAAATTTATTTAGAATTGCTAACGAGAAAACTACATTTGCTGACGCATTTCAACTCGCCGATAATGTTCTGCATATTGGCATAA
GAGGAGTAACTGATTTGATGATCATGCCAGGACTGATTAATCTTGATTTTGCTGATATAGAAACAGTAATGAGTGAGATGGGTAAAGCAATG
ATTGGTACTGGAGAGGCAGAAGGAGAAGATAGGGCAATTAGTGAAGCTGCAGAATTCATGAAAAAATTGAGGCAGATTTTGCGTTACATCG
GTTCATGTGATGGTGATATGGAAAAGGGGTCACTTCGCTGTGATGCAAATGTTTCTGTTCGCCCAAAGGGTAGTAGCACATTTGGCACTCGTT

GTGAAATAAAAAACTTAAATTCAATACGCTATATTGTACAAGCTATAGATTATGAAGCACAAAGGCAGATCAAAATTTTGGAAAGCGGAGGA
GAAATAAGTCAAGATACCTTATTGTTTGATGTCACTTTAGGAAAAACAAAAGTGATGAGAAGCAAAGAAGATTCAAGTGACTATAGATATTT
CCCTGAACCTGATTTGCTACCTGTTGAAATAAGCCAAGACAAACCACGTCTTCGCTCTGCTATATTTGCTGCGCGAAAGGAAAATCTACCAAA
AGATAAAATAGAAACAGCAATAAAAAATGCAGCTGGTAACGTTGCTGGAGAAAGTTATGAAGAAATACAATATGAAGGCTGCGGACCTTTT
GGTGCTGCACTTATTGTCCATGCTCTGACAAATAATCGCAACCGAACTGCTTCTGAGATACGTTATATCTTTTCTCGCAAAGGCGGTAATTTGG
GAGAAACAGGATGTGTGAGTTATCTTTTCGATCATGTAGGCTTAATTGTCTATAAAGCAGAGGGTATAAATTTTGAAGATTTATTTAACTATG
GAATTGCATTAGAAGTATTGAATGTTGAGGAAAATAACAAAGAAGAATTATATGTTATAACTTGTGAAGTAAAAGACTTTGGTAAAGTACGT
GAAGCTTTC
>Drosophila_melanogaster_wMel
AAGATGCCACTGTTTGTTTGGTCTGTCTTGCTAACAGCATTTATGTTGATTGTTGCCTTACCAGTGCTTGCCGGTGCTATAACTATGCTTCTTAC
TGATCGCAATATTGGTACTTCCTTTTTTGATCCTGCCGGTGGCGGCGATCCTGTGTTATTTCAACATCTATTTTGGTTTTTTGGTCATCCAGAAG
TTTACGTAATTATTTTTCCTGCATTTGGCATCATAAGTCAGGTTGTATCAACTTTTTCTCACAGACCTGTATTTGGTTACATAGGGATGGTTTAT
GCAATGATAGGTATAGCAGTATTTGGCTTTATGGTTTGGGCTCACCATATGTTCACTGTTGGGCTTAGTGCTGACGCTGCTGCATTTTTTCTTA
TTTTGAAACTTAATAGTTCCAACTCTTTACATTCAAAGGATCTAACCTCTGATCAGGCAATAACCTCTTCTGTGAAAGATGCGCTGCGTTTGGG
ATGCTTAGCTGTCGGATTTACTATATATCCTGGTTCTGCTAAGTGTTTCGATATGATGGAGGAAGCCCGTGGAATCATAGCTGAAGCCAAATC
TTATGGACTTGCAGTAGTGCTATGGTCTTATCCACGCGGTGAAGGGATTTCCAAAGAAGGTGAAACAGCAGTTGATGTTATTGCCTATGCTGC
GCACATGGCAGCTTTGCTTGGCGCTAATATAATAAAAGTAAAACTTCCAACTAAATATTTGGAAAGGGAGAAAATAGAAAAACCGGTGCAGC
ACCGGTAATTGCAAAAGCAGCCAGAGAAGCAAGAGCCGCAGTTAAGGATAGAGCGCCAAAAGAAAAAAAGATATTGACTGTTGGAGTTGTA
ACTAAACCGTTCGGTTTTGAAGGTGTGCGCCGTATGCGCATTGCAGAGCTTGGACTTGAAGAACTGCAAAAATACGTGGATACACTTATTGTC
ATTCCAAATCAGAATTTATTTAGAATTGCAAATGAAAAAACTACATTTTCTGATGCATTTAAACTTGCTGATAATGTTCTGCACATTGGCATCA
GAGGAGTAACTGACTTGATGGTCATGCCAGGGCTTATTAATCTTGACTTCGCTGATATAGAAACAGTAATGAGCGAGATGGGCAAAGCGATG
ATCGGCACCGGAGAGGCAGAAGGAGAAGATAGAGCAATTAGTGAAGCTGCAGAATGCATGAAAAAATTGAGGCAGATTTTGCGTTACATTG
GTTCGTGTGATGGTGATATGGAAAAGGGATCACTTCGTTGTGATGCAAATGTTTCTGTCCGCCTAAAAGGCAGTAGTACATTTGGCACTCGTT
GTGAAATAAAAAATCTGAACTCGATACGTTATATTGTGCAAGCTATAGACTATGAAATACAAAGACAAATTGAAATTTTAGAAAGTGGAGAA
GAAATAAGTCAAGATACCTTATTGTTTGACGTTGCTTCGGGAAAAACAAAAGTGATGAGAAGCAAAGAGGATGCAAGCGATTATAGATACTT
CCCTGAGCCTGATTTATTACCTGTTGAGGTAAGCCAGGATAAACCGCGCCTTCGCTCTGCTATATTTGCTGCACGCAAGGAAAATCTACCAAA
AGATAAAATAGAAACAGCAATAAAAAATGCAACTGGTAACGTTGCTGGAGAAAATTACGAGGAAATCCAATATGAAGGTCATGGGCCTTCT
GGCACTGCACTCATTGTCCATGTTTTGACTAATAATCGCAACCGAACTGCTTCTGAGGTACGTTATATATTTTCTCGCAAGGGTGGAAACTTGG
GAGAAACAGGAAGTGTTAGTTACCTTTTCGATCATGTAGGCTTAATCGTCTATAAAGCAGAGGGTGTGAATTTTGATGATTTATTCAGTCATG
GAATCGAATTAGAAGTATTGAATGTTGAGGAAAATGACAAAGAAGGATTACACGTTATAACTTGTGAAATAAAAGATTTTGGTAAAGTACGC
GATGCCTTT
>Megastigmus_pistaciae_wB
AAGATGCCACTGTTTGTTTTGTCTGTCTTGTTAACAGCATTTATGTTGATTGTTGCCTTACCAGTGCTTGCCGGTGCTATAACTATGCTTCTTAC
TGATCGCAATATTGGTACTTCCTTTTTTGATCCTGCCGGTGGTGGTGACCCTGTGTTATTTCAACATTTATTTTGGTTTTTTGGTCATCCGGAGG
TTTACGTAATTATTTTTCCTGCATTTGGCATCATAAGTCAGGTTGTATCAACTTTTTCTCACAGACCTGTATTTGGTTACATAGGAATGGTTTAT
GCAATGATAGGTATAGCAGTATTTGGCTTTATGGTTTGGGCTCACCATATGTTCACTGTTGGGCTTAGTGCTGACGCTGCTGCATTTTTTCTTA
TTTTnnnnnnnnnnnnnnnnnnnnnnnnnnnnnnnnnnnnnnnnnnnnnnnnnnnnnnnnnnnnnnnnnnnnnnnnnnnnnnnnnnnnnnnnnnnnnnnnnnnnnnnnnnnnnnnnnnnnnnnnnnnnnn
nnnnnnnnnnnnnnnnnnnnnnnnnnnnnnnnnnnnnnnnnnnnnnnnnnnnnnnnnnnnnnnnnnnnnnnnnnnnnnnnnnnnnnnnnnnnnnnnnnnnnnnnnnnnnnnnnnnnnnnnnnnnnnnn
nnnnnnnnnnnnnnnnnnnnnnnnnnnnnnnnnnnnnnnnnnnnnnnnnnnnnnnnnnnnnnnnnnnnnnnnnnnnnnnnnnnnnnnnnnnnnnnnnnnnnnnnnnnnnnnnAACAGGTGCTGCA
CCGGTAATTGCAAAAGCAGCAAGAGAAGCAAGAGCGGTAGTTAAAGATAAAGGAGCAAAAGAAAAAAAGATACTGACTGTTGGAGTTGTA
ACTAAGCCGTTCGGTTTTGAAGGTGTGCGACGTATGCGCATTGCAGAGCTTGGACTTGAAGAGTTGCAAAAATACGTAGATACACTTATTGTC
ATTCCCAATCAAAATTTATTTAGAATTGCTAACGAGAAAACTACATTTGCTGACGCATTTCAACTCGCCGATAATGTTCTGCATATTGGCATA
AGAGGAGTAACTGATTTGATGATCATGCCAGGACTGATTAATCTTGATTTTGCTGATATAGAAACAGTAATGAGTGAGATGGGTAAAGCAAT

GATTGGTACTGGAGAGGCAGAAGGAGAAGATAGGGCAATTAGTGAAGCTGCAGAATTCATGAAAAAATTGAGGCAGATTTTGCGTTACATC
GGTTCATGTGATGGTGATATGGAAAAGGGGTCACTTCGCTGTGATGCAAATGTTTCTGTTCGCCCAAAGGGCAGTAGCACATTTGGCACTCGT
TGTGAAATAAAAAACTTAAATTCAATACGTTATATTGTACAAGCTATAGATTATGAAGCACAAAGGCAGATCAAAATTTTGGAAAGCGGAGG
AGAAATAAGTCAAGATACCTTATTGTTTGATGTCACTTTAGGAAAAACAAAAGTGATGAGAAGCAAAGAAGATTCAAGTGACTATAGATATT
TCCCTGAACCTGATTTGCTACCTGTTGAAATAAGCCAAGACAAACCACGTCTTCGCTCTGCTATCTTTGCTGCGCGAAAGGAAAATCTACCAA
AAGATAAAATAGAAACAGCAATAAAAAATGCAGCTGGTAACGTTGCTGGAGAAAGTTATGAAGAAATACAATATGAAGGCTGCGGACCTTC
TGGTGCTGCACTTATTGTCCATGCTCTGACAAATAATCGCAACCGAACTGCTTCTGAGATACGTTATATCTTTTCTCGCAAAGGCGGTAATTTG
GGAGAAACAGGATGTGTGAGTTACCTTTTCGATCATGTAGGCTTAATTGTCTATAAAGCAGAGGGTATAAATTTTGAAGATTTATTTAACTAT
GGAATTGAATTAGAAGTATTGAATGTTGAGGAAAATAACAAAGAAGAATTATATGTTATAACTTGTGAAGTAAAAGACTTTGGTAAAGTACG
TGACGCTTTC
>Ostrinia_scapulalis_wOscaB
AAGATACCACTATTTGTTTGGTCTATTTTACTCACATCATTTATGTTAATTGTTGCCTTACCGGTACTTGCTGGTGCTATAACTATGCTGCTCAC
TGATCGTAATATAGGTACCTCCTTTTTTGATCCTGCTGGTGGTGGTGATCCTGTGTTATTTCAACATCTGTTTTGGTTTTTTGGTCATCCAGAAG
TTTACATAATTATTTTTCCTGCATTTGGTATCATCAGTCAGGTTGTGTCAACTTTTTCTCATAGGCCAGTATTTGGTTATATGGGAATGGTTTAT
GCTATGATAGGAATAGCAACGTTTGGCTTTATGGTTTGGGCTCACCATATGTTTACTGTTGGGCTTAGCGAGGATGCTGCTATATTTTTTCTTA
TTTTGAAGCTTAATAGCGCTAACTCTTTGCACTCAAAAAGCTTAACTTCAGATCAAGCAATAACTTCCTCTGTAAAAGATGCTCTACGTTTGGG
CTGCGTGGCTGTTGGGTTTACTATATATCCTGGTTCTGCTAAGTGTTTTGATATGATGGAAGAAGCTCGCAAAATTATAGCTGAGGCTAAATCT
TGTGGCCTTGCTGTAGTGCTATGGTCTTATCCACGTGGTGAAGGGATTTCCAAAGAAGGTGAAACAGCAGTTGATGTCATTGCTTATGCTGCG
CATATAGCGGCTTTGCTCGGTGCCAACATAATAAAAGTAAAACTTCCAACCAACCATATGGAAAGAGAAAAAATAGAAAAACAGGTGCTGC
ACCGGTAATTGCAAAAGCAGCCAGAGAAGCAAGAGCGGTAGTTAAAGATAAAGGAGCAAAAGAAAAAAAGATACTGACTGTTGGAGTCGT
AACTAAGCCGTTCGGTTTTGAAGGTGTGCGGCGTATGCGCATTGCAGAGCTTGGACTTGAAGAGTTGCAAAAATACGTAGATACACTTATTGT
CATTCCCAATCAAAATTTATTTAGAATTGCTAACGAGAAAACTACATTTGCTGACGCATTTCAACTCGCCGATAATGTTCTGCATATTGGCATA
AGAGGAGTAACTGATTTGATGATCATGCCAGGACTGATTAATCTTGATTTTGCTGATATAGAAACAGTCATGAGTGAGATGGGTAAAGCAAT
GATTGGTACTGGAGAGGCAGAAGGAGAAGATAGGGCAATTAGTGAAGCTGCAGAATTCATGAAAAAATTGAGGCAGATTTTGCGTTACATC
GGTTCATGTGATGGTGATATGGAAAAGGGGTCACTTCGCTGTGATGCAAATGTTTCTGTTCGCCCAAAGGGTAGTAGCACATTTGGCACTCGT
TGTGAAATAAAAAACTTAAATTCAATACGTTATATTGTACAAGCTATAGATTATGAAGCACAAAGGCAGATCAAAATTTTGGAAAGCGGAGG
AGAAATAAGTCAAGATACCTTATTGTTTGATGTCACTTTAGGAAAAACAAAAGTGATGAGAAGCAAAGAAGATTCAAGTGACTATAGATATT
TCCCTGAACCTGATTTGCTACCTGTTGAAATAAGCCAAGACAAACCACGTCTTCGCTCTGCTATCTTTGCTGCGCGAAAGGAAAATCTACCAA
AAGATAAAATAGAAACAGCAATAAAAAATGCAGCTGGTAACGTTGCTGGAGAAAGTTATGAAGAAATACAATATGAAGGCTGCGGACCTTC
TGGTGCTGCACTTATTGTCCATGCTCTGACAAATAATCGCAACCGAACTGCTTCTGAGATACGTTATATCTTTTCTCGCAAAGGCGGTAATTTG
GGAGAAACAGGATGTGTGAGTTACCTTTTTGATCATGTAGGCTTAATTGTCTATAAAGCAGAGGGTATAAATTTTGAAGATTTATTTAACTAT
GGAATTGAATTAGAAGTATTGAATGTTGAGGAAAATAACAAAGAAGAATTATATGTTATAACTTGTGAAGTAAAAGACTTTGGTAAAGTACG
TGACGCTTTC
>Drosophila_bifasciata_wBif
AAGATGCCACTGTTTGTTTGGTCTGTCTTGCTAACAGCATTTATGTTGATTGTTGCTTTACCAGTGCTTGCCGGTGCTATAACTATGCTTCTTAC
TGATCGCAATATTGGCACTTCCTTTTTTGATCCTGCCGGTGGTGGCGATCCTGTGTTATTTCAACATCTATTTTGGTTTTTTGGTCATCCAGAAG
TTTACGTAATTATTTTTCCTGCATTTGGCATCATAAGTCAGGTTGTATCAACTTTTTCTCACAGACCTGTATTTGGTTACATAGGGATGGTTTAT
GCAATGATAGGTATAGCAGTATTTGGCTTTATGGTTTGGGCTCATCATATGTTCACTGTTGGGCTTAGTGCTGACGCTGCTGCATTTTTTCTTAT
TTTGAAACTTAATAGTTCCAACTCCTTACATTCAAAGGATCTGACCTCTGATCAAGCAATAACCTCTTCTGTGAAAGATGCACTGCGTTTGGGC
TGCTTGGCTGTTGGATTTACTATATATCCTGGTTCTGCTAAGTGTTTCGATATGATGGAAGAAGCCCGTGGAATCATAGCTGAAGCCAAATCTT
ACGGGCTTGCAGTAGTGCTATGGTCTTATCCACGCGGTGAAGGAATTTCCAAAGAAGGTGAAACAGCGGTTGATGTTATTGCCTATGCTGCAC
ACATGGCAGCTTTGCTTGGCGCTAATATAATAAAAGTAAAACTTCCAACTAAATATTTGGAAAGGGAGAAAATAGAAAAACCGGTGCAGCAC
CGGTAATTGCAAAAGCAGCCAGAGAAGCAAGAGCCGCAGTTAAGGATAGAGCGCCAAAAGAAAAAAAGATATTGACTGTTGGAGTTGTAAC

TAAACCGTTCGGTTTTGAAGGTGTGCGCCGTATGCGCATTGCAGAGCTTGGACTTGAAGAACTGCAAAAATACGTGGATACACTTATTGTCAT
TCCAAATCAGAATTTATTTAGAATTGCAAATGAAAAAACCACATTTTCTGATGCATTTAAACTTGCTGATAATGTTCTGCACATTGGTATCAG
AGGAGTAACTGACTTGATGGTCATGCCAGGGCTTATCAATCTTGACTTCGCTGATATAGAAACAGTAATGAGCGAGATGGGCAAAGCGATGA
TCGGCACCGGAGAGGCAGAAGGAGAAGATAGAGCAATTAGTGAAGCTGCAGAATGCATGAAAAAATTGAGGCAGATTTTGCGTTACATTGG
TTCGTGTGATGGTGATATGGAAAAGGGATCACTTCGTTGTGATGCAAATGTTTCTGTCCGCCTAAAAGGCAGTAGTACATTTGGCACCCGCTG
TGAAATAAAAAATCTGAACTCGATACGTTATATTGTGCAAGCTATAGACTATGAAATACAAAGACAAATTGAAATTTTAGAAAGTGGGGAAG
AAATAAGTCAAGATACCTTATTGTTTGATGTTGCTTTGGGAAAAACAAAAGTGATGAGAAGCAAAGAGGATGCAAGCGACTATAGATACTTC
CCTGAGCCTGATTTATTACCTGTTGAGGTAAGCCAGGATAAACCGCGCCTTCGCTCTGCTATATTTGCTGCACGCAAGGAAAATCTACCAAAA
GATAAAATAGAAACAGCAATAAAAAATGCAACTGGTAACGTTGCTGGAGAAAATTACGAGGAAATACAATATGAAGGTCATGGGCCTTCTG
GCACTGCACTCATTGTCCATGCCTTGACTAATAACCGCAACCGTACTGCTTCTGAGGTACGTTATATCTTTTCTCGTAAAGGTGGAAATTTAGG
AGAAACAGGAAGTGTTAGTTACCTTTTCGATCATGTAGGCTTAATCGTCTATAAAGCAGAGGGTGTGAATTTTGATGATTTATTCAGTCATGG
AATCGAATTAGAAGTATTGAATGTTGAGGAAAATGACAAAGAAGGATTACACGTTATAACTTGTGAAATAAAAGATTTTGGTAAAGTACGCG
ATGCCTTT
>Tribolium_confusum_wCon
AAGATGCCACTATTTGTTTGGTCTGTTTTACTAACGTCATTTATGTTAATTGTTGCCTTACCGGTACTTGCTGGTGCTATAACTATGCTGCTAAC
AGATCGCAATATTGGTACTTCCTTTTTTGATCCTGCTGGTGGCGGTGATCCTGTGTTATTTCAACACCTGTTTTGGTTTTTTGGTCATCCAGAAG
TTTACATAATTATTTTTCCTGCATTTGGCATTATAAGCCAAGTCGTGTCAACTTTTTCCCATAGGCCAGTTTTTGGTTATAAGGGAATGGTTTAT
GCCATGATAGGTATAGCAGCATTTGGTTTTATGGTTTGGGCTCACCATATGTTTACTGTTGGACTTAGCGAAGATGCTGCTGTATTTTTCCTTA
TTTTGAAGCTTAATAGTGCCAACTCCTTACACTCAAAAAGCTTAACTTCTGATCAAGCAATAACTGCTTCTGTAAAAGATGCACTGCGTTTAG
GCTGTACGGCTGTTGGGTTTACTATATATCCTGGTTCTGCTAAATGTTTTGATATGATGGAAGAAGCTCGCGAAATTATAGCTGAGGCTAAGT
CCTTCGGACTTGCTGTGGTGCTATGGTCTTATCCACGTGGTGAAGGGATTTCCAAAGAAGGTGAAACAGCAGTTGATGTAATTGCTTATGCTG
CGCATATAGCGGCTTTGCTTGGTGCCAACATAATAAAAGTAAAACTTCCAACCAACCATCTGGAAAGAGAAAAAATAGAAAAACAGGTGCTG
CACCGGTAATTGCAAAGGCAGCCAGAGAAGCAAGAGCGATAGTTAAAGATAAAGGAGCAAAAGAAAAAAAGATACTGACTGTTGGAGTTGT
AACTAAGCCGTTCGGTTTTGAAGGTGTGCGACGTATGCGCATTGCAGAGCTTGGACTTGAAGAGTTGCAAAAATACGTAGATACACTTATTGT
CATTCCCAATCAAAATTTATTTAGAATTGCTAACGAGAAAACTACATTTGCTGACGCATTTCAACTCGCCGATAATGTTCTGCATATTGGCATA
AGAGGAGTAACTGATTTGATGATCATGCCAGGACTGATTAATCTTGATTTTGCTGATATAGAAACAGTAATGAGTGAGATGGGTAAGGCAAT
GATTGGTACTGGAGAGGCAGAAGGAGAAGATAGGGCAATTAGTGAAGCTGCAGAATTCATGAAAAAATTGAGGCAGATTTTGCGTTATATC
GGTTCATGTGATGGTGATATGGAAAAGGGATCACTTCGCTGTGATGCAAATGTTTCCATTCGCCCAAAGGGTAGTAGCGTATTTGGCACTCGC
TGTGAAATAAAAAATTTAAATTCAATACGTTATATTGTACAAGCTATAGATTATGAAGCACAAAGGCAAATCAAAATTCTGGAAAGCGGAGG
AGAAATAAGTCAAGATACCTTATTGTTTGATGTCACTTTAGGAAAAACAAAAGTGATGAGAAGCAAAGAAGATTCAAGTGACTATAGATATT
TCCCTGAACCTGATTTGCTACCTATTGAAATAAGCCAAGACAAACCACGTCTTCGCTCTGCTATCTTTGCTGCGCGAAAGGAAAATCTACCAA
AAGATAAAATAGAAACAGCAATAAAAAATGCAGCTGGTAACGTTGCTGGAGAAAGTTATGAAGAAATACAATATGAAGGCTGCGGACCTTC
TGGTGCTGCACTTATTGTCCATGCTCTGACAAATAATCGCAACCGAACTGCTTCTGAGATACGTTATATCTTTTCTCGCAAAGGCGGTAATTTG
GGAGAAACAGGATGTGTGAGTTACCTTTTCGATCATGTAGGCTTAATTGTCTATAAAGCAGAGGGTATAAATTTTGAAGATTTATTTAACTAT
GGAATTGAATTAGAAGTATTGAATGTTGAGGAAAATAACAAAGAAGAATTATATGTTATAACTTGTGAAGTAAAAGACTTTGGTAAAGTACG
TGACGCTTTC
>Trichogramma_deion_wDei
AAGATGCCACTATTTGTCTGGTCTATTTTACTCACATCATTCATGTTAATTGTTGCCTTGCCGGTACTTGCTGGTGCTATAACTATGCTGCTCAC
TGATCGTAATATAGGTACCTCCTTTTTTGATCCTGCTGGTGGTGGTGATCCTGTGTTATTTCAACATCTGTTTTGGTTTTTTGGTCATCCAGAAG
TTTACATAATTATTTTTCCTGCATTTGGTATCATTAGTCAGATTGTGTCAACTTTTTCTCATAGGCCAGTATTTGGTTATATGGGAATGGTTTAT
GCTATGATAGGAATAGCAACGTTTGGCTTTATGGTTTGGGCTCACCATATGTTTACTGTTGGGCTTGGCGAGGATGCTACTATATTTTTTCTTA
TTTTGAAGCTTAATAGCGCTAACTCTTTGCACTCAAAAAGCTTAACTTCAGATCAAGCAATAACTTCCTCTGTAAAAGATGCTCTACGTTTGGG
CTGCGTTGCTGTTGGGTTTACTATATATCCTGGTTCTGCTAAGTGTTTTGATATGATGGAAGAAGCTCGCAAAATTATAGCTGAGGCTAAATCT

TGTGGCCTTGCTGTAGTGTTATGGTCTTATCCACGTGGTGAAGGGATTTCCAAAGAAGGTGAGACAGCAGTTGATGTCATTGCTTATGCTGCG
CATATAGCGGCTTTGCTCGGTGCCAACATAATAAAAGTAAAACTTCCAACCAACCATATGGAAAGAGAAAAAATAGAAAAACAGGTGCTGC
ACCGGTAATTGCAAAAGCAGCCAGAGAAGCAAGAGCGGTGGTTAAAGATAAAGGAGCAAAAGAAAAAAAGATACTGACTGTTGGAGTTGTA
ACTAAGCCGTTCGGTTTTGAAGGTGTGCGACGTATGCGCATTGCAGAGCTTGGACTTGAAGAGTTGCAAAAATACGTAGATACACTTATTGTC
ATTCCCAATCAAAATTTATTTAGAATTGCTAACGAGAAAACTACATTTGCTGATGCATTTCAACTCGCTGATAATGTTCTGCATATTGGCATAA
GAGGAGTAACTGATTTAATGGTCATGCCAGGACTGATTAATCTTGATTTTGCTGATATAGAAACGGTAATGAGTGAGATGGGTAAAGCAATG
ATTGGTACTGGGGAGGCGGAAGGAGAAGATAGGGCAATTAGTGAAGCTGCAGAATTCATGAAAAAATTGAGGCAGATTTTGCGTTACATCG
GTTCATGTGATGGTAATATGGAAAAGGGGTCACTTCGCTGTGATGCAAATGTTTCTGTTCGCCCAAAGGGTAGTAGCACATTTGGCACTCGTT
GTGAAATAAAAAACTTAAATTCAATACGTTATATTGTACAAGCTATAGATTATGAAGCACAAAGGCAGATCAAAATTTTGGAAAGCGGAGGA
AAAATAAGTCAAGATACCTTATTGTTTGATGTCACTTTAGGAAAAACAAAAGTGATGAGAAGCAAAGAAGATTCAAGTGACTATAGATATTT
CCTTGAACCTGATTTGCTACCTGTTGAAATAAGCCAACACAAACCACGTCTTCGCTCTGCTATCTTTGCTGCGCGAAAGGAAAATCTACCAAA
AGATAAAATAGAAACAGCAATAAAAAATGCAGCTGGTAACGTTGCTGGAGAAAGTTATGAAGAAATACAATATGAAGGCTGCGGACCTTCT
GGTGCTGCACTTATTGTCCATGCTCTGACAAATAATCGCAACCGAACTGCTTCTGAGATACGTTATATCTTTTCTCGCAAAGGCGGTAATTTGG
GAGAAACAGGATGTGTGAGTTACCTTTTCGATCATGTAGGCTTAATTGTCTATAAAGCAGAGGGTGTGGATTTTGATGATTTATTTAACTATG
GAATTGAATTAGAAGTATTGAATGTTGAGAAAAATAACAAAGAAGAATTGTATGTTATAACTTGTGAAGTAAAAGACTTTGGTAAAGTACGT
GATACTTTC
>Culex_quinquefasciatus_wPip
AAGATACCACTATTTGTTTGGTCTATTTTACTCACGTCATTTATGTTAATTGTTGCCTTACCGGTACTTGCTGGTGCTATAACTATGCTGCTCAC
TGATCGTAATATAGGTACCTCCTTTTTTGATCCTGCTGGTGGTGGTGATCCTGTGTTATTTCAACATCTGTTTTGGTTTTTTGGTCATCCAGAAG
TTTACATAATTATTTTTCCTGCATTTGGTATCATCAGTCAGATTGTGTCAACTTTTTCTCATAGGCCAGTATTTGGTTATATGGGAATGGTTTAT
GCCATGATAGGAATAGCAACGTTTGGCTTCATGGTTTGGGCTCACCATATGTTTACTGTTGGGCTTAGCGAGGATACTGCTATATTTTTTCTTA
TTTTGAAGCTTAATAGCGCTAACTCTTTGCACTCAAAAAGCTTAACTTCAGATCAAGCAATAACTGCCTCTGTAAAAGATGCGCTGCGTTTGG
GCTGCATGGCTGTTGGGTTTACTATATATCCTGGTTCTGCTAAGTGTTTTGATATGATGGAAGAAGCTCGCAAAATTATAGCTGAGGCTAAAT
CTTGTGGCCTTGTTGTAGTGCTATGGTCTTATCCACGTGGTGAAGGGGTTTCCAAAGAAGGTGAAACAGCAGTTGATGTGATTGCCTATGCTG
CGCATATAGCGGCTTTGCTCGGTGCCAACATAATAAAAGTAAAACTTCCAACCAATCATCTGGAAAGAGAAAAAATAGAAAAACAGGCGCT
GCACCGGTAATTGCAAAAGCAGCCAGAGAAGCAAGAGCGGTAGTTAAAGATAAAGGAGCAAAAGAAAAAAAGATACTGACTGTTGGAGTT
GTAACTAAGCCGTTCGGTTTTGAAGGTGTGCGACGTATGCGCATTGCAGAGCTTGGACTTGAAGAGTTGCAAAAATACGTAGATACACTTATT
GTCATTCCTAATCAAAATTTATTTAGAATTGCTAACGAGAAAACTACATTTGCTGACGCATTTCAACTCGCCGATAATGTTCTGCATATTGGCA
TAAGAGGAGTAACTGATTTGATGATCATGCCAGGACTGATTAATCTTGATTTTGCTGATATAGAAACAGTAATGAGTGAGATGGGTAAAGCA
ATGATTGGTACTGGAGAGGCAGAAGGAGAAGATAGGGCAATTAGTnnnnnnnnnnnnnnnnnnnnnnnnnnnnnnnnnnnnnnnnnnnnnnnnnnnnnnnnnnnnnnnn
nnnnnnnnnnnnnnnnnnnnnnnnnnnnnnnnnnnnnnnnnnnnnnnnnnnnnnnnnnnnnnnnnnnnnnnnnnnnnnnnnnnnnnnnnnnnnnnnnnnnnnnnnnnnnnnnnnnnnnnnnnnnnnnnnn
nnnnnnnnnnnnnnnnnnnnnnnnnnnnnnnnnnnnnnnnnnnnnnnnnnnnnnnnnnnnnnnnnnnnnnnnnnnnnnnnnnnnnnnnnnnnnnnnnnnnnnnnnnnnnnnnnnnnnnnnnnnnnnnnnnnn
nnnnnnnnnnnnnnnnnnnnnnnnnnnnnnnnnnnnnnnnnnnnnnnnnnnnnnnCCACGTCTTCGCTCTGCTATCTTTTCTGCGCGAAAGGAAAATCTACCAAAAGAT
AAAATAGAAACAGCAATAAAAAATGCAGCTGGTAACGTTGCTGGAGAAAGTTATGAAGAAATACAATATGAAGGCTGCGGACCTTCTGGTG
CTGCACTTATTGTCCATGCTCTGACAAATAATCGCAACCGAACTGCTTCTGAGATACGTTATATCTTTTCTCGCAAAGGCGGTAATTTGGGAG
AAACAGGATGTGTGAGTTACCTTTTCGATCATGTAGGCTTAATTGTCTATAAAGCAGAGGGTATAAATTTTGAAGATTTATTTAACTATGGAA
TTGAATTAGAAGTATTGAATGTTGAGGAAAATAACAAGGAAGAATTATATGTTATAACTTGTGGAGTGAAAGATTTTGGTAGAGTGCGTGAT
GCTTTC
>Eurema_blanda_wEbl
AAGATACCACTATTTGTTTGGTCTATTTTACTCACGTCATTTATGTTAATTGTTGCCTTACCGGTACTTGCTGGTGCTATAACTATGCTGCTCAC
TGATCGTAATATAGGTACCTCCTTTTTTGATCCTGCTGGTGGTGGTGATCCTGTGTTATTTCAACATCTGTTTTGGTTTTTTGGTCATCCAGAAG
TTTACATAATTATTTTTCCTGCATTTGGTATCATCAGTCAGATTGTGTCAACTTTTTCTCATAGGCCAGTATTTGGTTATATGGGAATGGTTTAT
GCCATGATAGGAATAGCAACGTTTGGCTTCATGGTTTGGGCTCACCATATGTTTACTGTTGGGCTTAGCGAGGATGCTGCTATATTTTTTCTTA

TTTTGAAGCTTAATAGCGCTAACTCTTTGCACTCAAAAAGCTTAACTTCAGATCAAGCAATAACTGCCTCTGTAAAAGATGCGCTGCGTTTGG
GCTGCATGGCTGTTGGGTTTACTATATATCCTGGTTCTGCTAAGTGTTTTGATATGATGGAAGAAGCTCGCAAAATTATAGCTGAGGCTAAAT
CTTGTGGCCTTGTTGTAGTGCTATGGTCTTATCCACGTGGTGAAGGGGTTTCCAAAGAAGGTGAAACAGCAGTTGATGTGATTGCCTATGCTG
CGCATATAGCGGCTTTGCTCGGTGCCAACATAATAAAAGTAAAACTTCCAACCAATCATCTGGAAAGAGAAAAAATAGAAAAACAGGTGCTG
CACCGGTAATTGCAAAAGCAGCCAGAGAAGCAAGAGCGGTAGTTAAAGATAAAGGAGCAAAAGAAAAAAAGATACTGACTGTTGGAGTCGT
AACTAAGCCGTTCGGTTTTGAAGGTGTGCGGCGTATGCGCATTGCAGAGCTTGGACTTGAAGAGTTGCAAAAATACGTAGATACACTTATTGT
CATTCCCAATCAAAATTTATTTAGAATTGCTAACGAGAAAACTACATTTGCTGACGCATTTCAACTCGCCGATAATGTTCTGCATATTGGCATA
AGAGGAGTAACTGATTTGATGATCATGCCAGGACTGATTAATCTTGATTTTGCTGATATAGAAACAGTCATGAGTGAGATGGGTAAAGCAAT
GATTGGTACTGGAGAGGCAGAAGGAGAAGATAGGGCAATTAGTnnnnnnnnnnnnnnnnnnnnnnnnnnnnnnnnnnnnnnCGTTACATCGGTTCATGTGA
TGGTGATATGGAAAAGGGGTCACTTCGCTGTGATGCAAATGTTTCTGTTCGCCCAAAGGGCAGTAGCACATTTGGCACTCGTTGTGAAATAAA
AAACTTAAATTCAATACGTTATATTGTACAAGCTATAGATTATGAAGCACAAAGGCAGATCAAAATTTTGGAAAGCGGAGGAGAAATAAGTC
AAGATACCTTATTGTTTGATGTCACTTTAGGAAAAACAAAAGTGATGAGAAGCAAAGAAGATTCAAGTGACTATAGATATTTCCCTGAACCT
GATTTGCTACCTGTTGAAATAAGCCAAGACAAACCACGTCTTCGCTCTGCTATCTTTTCTGCGCGAAAGGAAAATCTACCAAAAGATAAAATA
GAAACAGCAATAAAAAATGCAGCTGGTAACGTTGCTGGAGAAAGTTATGAAGAAATACAATATGAAGGCTGCGGACCTTCTGGTGCTGCACT
TATTGTCCATGCTCTGACAAATAATCGCAACCGAACTGCTTCTGAGATACGTTATATCTTTTCTCGCAAAGGCGGTAATTTGGGAGAAACAGG
ATGTGTGAGTTACCTTTTCGATCATGTAGGCTTAATTGTCTATAAAGCAGAGGGTATAAATTTTGAAGATTTATTTAACTATGGAATTGAATTA
GAAGTATTGAATGTTGAGGAAAATAACAAGGAAGAATTATATGTTATAACTTGTGAAGTGAAAGATTTTGGTAGAGTGCGTGATGCTTTC
>Philaenus_spumarius
AAAATGCCACTATTTGTTTGGTCTGTTTTACTCACGTCATTTATGTTAATTGTTGCCTTACCGGTACTTGCTGGTGCTATAACTATGCTGCTCAC
TGATCGCAATATTGGTACTTCCTTTTTTGATCCTGCTGGTGGTGGTGATCCTGTGTTATTTCAACATCTGTTTTGGTTTTTTGGTCATCCAAAAG
TTTACATAATTATTTTTCCTGCATTTGGTATCATAAGTCAAATCGTATCAACTTTTTCCCATAGGCCAGTATTTGGTTATATGGGAATGGTTTAT
GCCATGATAGGAATAGCAACGTTTGGCTTCATGGTTTGGGCTCACCATATGTTTACTGTTGGGCTTAGCGAAGATGCTGCTATATTTTTTCTTA
TTTTnnnGCTTAATAGTGCTAACTCCTTGCACTCGAAAAACTTAACTTCTGATCAAGCAATAACTGCTTCTGTAAAAGATGCGCTGCGTTTGGGT
TGCGTAGCTGTTGGGTTTACTATATATCCTGGTTCTGCTAAGTGCTTCGATATGATGGAAGAAGCTCGCGAAATTATAGCTGAGGCTAAGTCT
TGCGGCCTTGCCGTAGTGCTATGGTCTTATCCACGCGGTGAAGGAATTTCCAAAGAAGGCGAAACAGCAGTTGATGTCATTGCTTATGCTGCG
CATATAGCGGCTTTACTTGCCGCTAATATAATCAAAGTAAAACTTCCAATTAACTATTTGGAAAGGGAGAAAATAGAAAAACAGGTGCTGCA
CCGGTAATTGCAAAAGCAGCCAGAGAAGCAAGAGCGGTAGTTAAAGATAAAGGAGCAAAAGAAAAAAAGATACTGACTGTTGGAGTTGTAA
CTAAGCCGTTCGGTTTTGAAGGTGTGCGACGTATGCGCATTGCAGAGCTTGGACTTGAAGAGTTGCAAAAATATGTAGACACACTTATTGTCA
TTCCCAATCAAAATTTATTTAGAATTGCTAACGAGAAAACTACATTTGCTGACGCATTTCAACTCGCCGATAATGTTCTGCATATTGGCATAA
GAGGAGTAACTGATTTGATGATCATGCCAGGACTGATTAATCTTGATTTTGCTGATATAGAAACAGTAATGAGTGAGATGGGTAAAnnnnnnnnn
nnnnnnnnnnnnnnnnnnnnnnnnnnnnnnnnnnnGAAGCTGCAGAATTCATGAAAAAATTGAGGCAGATTTTGCGTTACATCGGTTCATGTGATGGTG
ATATGGAAAAGGGGTCCCTTCGTTGTGATGCAAATGTTTCTGTTCGCCCAAAGGGTAGTAGCGCATTTGGCACTCGTTGTGAAATAAAAAACT
TAAATTCAATACGTTATATTGTACAAGCTATAGATTATGAAGCACAAAGGCAAATCAAATTTTTGGAAAGCGGAGGAAAAATAAGTCAAGAT
ACCTTATTGTTTGATGTCACTTTAGGAAAAACAAAAGTGATGAGAAGCAAAAAAGATTCAAGTGACTATAGATATTTCCCTGAACCTGATTTG
CTACCTGTTGAAATAAGCCAAGACAAACCACGTCTTCGCTCTGCTATCTTTGCTGCGCGAAAGGAAAATCTACCAAAAGATAAAATAGAAAC
AGCAATAAAAAATGCAGCTGGTAACGTTGCTGGAGAAAGTTATGAAGAAATACAATATGAAGGCTGCGGACCTTCTGGTGCTGCACTTATTG
TCCATGCTCTGACAAATAATCGCAACCGAACTGCTTCTGAGATACGTTATATCTTTTCTCGCAAAGGCGGTAATTTGGGAGAAACAGGATGTG
TGAGTTACCTTTTCGATCATGTAGGCTTAATTGTCTATAAAGCAGAGGGTATAAATTTTGAAGATTTATTTAACTATGGAATTGAATTAGAAGT
ATTGAATGTTGAGGAAAATAACAAAGAAGAATTATATGTTATAACTTGTGAAGTAAAAGACTTTGGTAAAGTACGTGACGCTTTC
>Xyleborus_seiryorensis_wXse
AAGATGCCACTATTTATTTGGTCTGTCTTGCTAACAGCATTTATGTTGATTGTCGCCTTACCAGTGCTTGCCGGTGCTATAACTATGCTTCTTAC
TGATCGCAATATTGGCACTTCCTTTTTTGATCCTGCAGGTGGTGGTGACCCTGTGTTATTTCAACATTTATTTTGGTTTTTTGGTCATCCAGAAG
TTTACGTAATTATTTTTCCTGCATTTGGCATCATAAGTCAGGTTGTATCAACTTTTTCTCACAGACCTGTATTTGGTTACATAGGGATGGTTTAT

GCAATGATAGGTATAGCAGTATTTGGCTTTATGGTTTGGGCTCATCATATGTTCACTGTTGGGCTTAGTGCTGACGCTGCTGCATTTTTTCTTAT
TTTnnnnnnnnnnnnnnnnnnnnnnnnnnnnnnnnnnnnnnnnnnnnnnnnnnnnnnnnnnnnnnnnnnnnnnnnnnnnnnnnnnnnnnnnnnnnnnnnnnnnnnnnnnnnnnnnnnnnnnnnnnnnnnnnnnn
nnnnnnnnnnnnnnnnnnnnnnnnnnnnnnnnnnnnnnnnnnnnnnnnnnnnnnnnnnnnnnnnnnnnnnnnnnnnnnnnnnnnnnnnnnnnnnnnnnnnnnnnnnnnnnnnnnnnnnnnnnnnnnnnnnnnnnnnnn
nnnnnnnnnnnnnnnnnnnnnnnnnnnnnnnnnnnnnnnnnnnnnnnnnnnnnnnnnnnnnnnnnnnnnnnnnnnnnnnnnnnnnnnnnnnnnnnnnnnnnnnnnnnnnnnnnnnAACCGGTGCTGCAC
CGGTAATTGCAAAAGCAGCCAGAGAAGCAAGAGCTGCAGTTAAGGATAGAGCGCCAAAAGAAAAAAAGATATTGACTGTTGGAGTTGTAAC
TAAACCGTTCGGTTTTGAAGGTGTGCGCCGTATGCGCATTGCAGAGCTTGGACTTGAAGAACTGCAAAAATACGTGGATACACTTATTGTCAT
TCCAAATCAGAATTTATTTAGAATTGCAAATGAAAAAACTACATTTCTGATGCATTTAAACTTGCTGATAATGTTCTGCATATTGGCATCAGA
GGAGTAACTGACTTGATGGTCATGCCAGGGCTTATTAATCTTGACTTCGCTGATATAGAAACAGTAATGAGCGAGATGGGCAAAGCGATGAT
CGGCACCGGAGAGGCAGAAGGAGAAGATAGAGCAATTAGTGAAGCTGCAGAATGCATGAAAAAATTGAGGCAGATTTTGCGTTACATTGGT
TCGTGTGATGGTGATATGGAAAAGGGATCACTTCGTTGTGATGCAAATGTTTCTGTCCGCCTAAAAGGCAGTAGCACATTTGGCACTCGTTGT
GAGATAAAAAATCTGAACTCGATACGTTATATTGTGCAAGCTATAGACTATGAAATACAAAGACAAATTGAAATTTTAGAAGGTGGGGAAGA
AATAAGTCAAGATACCTTATTGTTTGACGTTGCTTCGGGAAAAACAAAAGTGATGCGAAACAAAGAAGATGCAAGCGACTATAGATACTTCC
CTGAGCCTGATTTATTACCTGTTGAGGTAAGCCAGGAGAAACCGCGCCTTCGCTCTGCTATATTTGCTGCACGCAAGGAAAATCTACCAAAAG
ATAAAAGAGAAACAGCAATAAAAAATGCAACTGGTAACGTTGCTGGAGAAAATTACGAGGAAATACAATATGAAGGTCATGGGCCTTTTGG
CACTGCACTCATTGTCCATGCCTTGACTAATAACCGCAACCGTACTGCTTCTGAGGTGCGTTATATTTTTTCTCGCAAAGGTGGAAATTTAGGA
GAAACAGGAAGCGTTAGTTATCTTTTTGATCATGTAGGTTTAATTGTCTATAAAGCAGAGGGTGTGAATTTTGACGATTTATTCAGTCATGGA
ATCGAATTAGAAGTATTGAATGTTGAGGAAAATGACAAAGAAGGATTACACGTTATAACTTGTGAAATAAAAGATTTTGGTAAAGTACGCGA
TGCCTTT
>Culex_decens_wDec
AAGATACCACTATTTGTTTGGTCTATTTTACTCACGTCATTTATGTTAATTGTTGCCTTACCGGTACTTGCCGGTGCTATAACTATGCTGCTCAC
TGATCGTAATATAGGTACCTCCTTTTTTGATCCTGCTGGTGGTGGTGATCCTGTTTTATTTCAACATCTGTTTTGGTTTTTTGGTCATCCAGAAG
TTTACATAATTATTTTTCCTGCATTTGGTATCATCAGTCAGATTGTGTCAACTTTTTCTCATAGGCCAGTATTTGGTTATATGGGAATGGTTTAT
GCCATGATAGGAATAGCAACGTTTGGCTTCATGGTTTGGGCTCACCATATGTTTACTGTTGGGCTTAGCGAGGATGCTGCTATATTTTTTCTTA
TTTTGAAGCTTAATAGTGCCAACTCCTTACACTCAAAAAGCTTAACTTCTGATCAAGCAATAACTGCTTCTGTAAAAGATGCACTGCGTTTAG
GCTGTGCGGCTGTTGGGTTTACTATATATCCTGGTTCTGCTAAATGTTTTGATATGATGGAAGAAGCTCGCAAAATTATAGCTGAGGCTAAGT
CCTTCGGACTTGCTGTGGTGCTATGGTCTTATCCACGTGGTGAAGGGATTTCCAAAGAAGGTGAAACAGCAGTTGATGTAATTGCTTATGCTG
CGCATATAGCGGCTTTGCTCGGTGCCAACATAATAAAAGTAAAACTTCCAACCAACCATCTGGAAAGAGAAAAAATAGAAGAACAGGTGCT
GCACCGGTAATTGCAAAAGCAGCCAGAGAAGCAAGAGCGGTAGTTAAAGATAAAGGAGCAAAAGAAAAAAAGATACTGACTGTTGGAGTT
GTAACTAAGCCGTTCGGTTTTGAAGGTGTGCGACGTATGCGCATTGCAGAGCTTGGACTTGAAGAGTTGCAAAAATACGTCGATACACTTATT
GTCATTCCCAATCAAAATTTATTTAGAATTGCTAACGAGAAAACTACATTTGCTGACGCATTTCAACTCGCCGATAATGTTCTGCATATTGGCA
TAAGAGGAGTAACTGATTTGATGATCATGCCAGGACTGATTAATCTTGATTTTGCTGATATAGAAACAGTAATGAGTGAGATGGGTAAAGCA
ATGATTGGTACTGGAGAAGCAGAAGGAGAAGATAGGGCAATTAGTGAAGCTGCAGAATTCATGAAAAAATTGAGGCAGATTTTGCGTTACA
TCGGTTCATGTGATGGTGATATGGAAAAGGGGTCACTTCGCTGTGATGCAAATGTTTCTGTTCGCCCAAAGGGTAGTAGCACATTTGGCACTC
GTTGTGAAATAAAAAACTTAAATTCAATACGTTATATTGTACAAGCTATAGATTATGAAGCACAAAGGCAGATCAAAATTTTGGAAAGCGGA
GGAGAAATAAGTCAAGATACCTTATTGTTTGATGTCACTTTAGGAAAAACAAAAGTGATGAGAAGCAAAGAAGATTCAAGTGACTATAGATA
TTTCCCTGAACCTGATTTGCTACCTGTTGAAATAAGCCAAGACAAACCACGTCTTCGCTCTGCTATCTTTGCTGCGCGAAAGGAAAATCTACC
AAAAGATAAAATAGAAACAGCAATAAAAAATGCAGCTGGTAACGTTGCTGGAGAAAGTTATGAAGAAATACAATATGAAGGCTGCGGACCT
TCTGGTGCTGCACTTATTGTCCATGCTCTGACAAATAATCGCAACCGAACTGCTTCTGAGATACGTTATATCTTTTCTCGCAAAGGCGGTAATT
TGGGAGAAACAGGATGTGTGAGTTACCTTTTCGATCATGTAGGCTTAATTATCTATAAAGCATGGGGTATAAATTTTGAAGATTTATTTAACT
ATGGAATTGAATTAGAAGTATTGAATGTTGAGGAAAATAACAAAGAAGAATTATATGTTATAACTTGTGAAGTAAAAGGCTTTGGTAAAGTA
CGTGACGCTTTC
>Euwallacea_interjectus_wEi

AAGATGCCACTATTTATTTGGTCTGTCTTGCTAACAGCATTTATGTTGATTGTCGCCTTACCAGTGCTTGCCGGTGCTATAACTATGCTTCTTAC
TGATCGCAATATTGGCACTTCCTTTTTTGATCCTGCAGGTGGTGGTGACCCTGTGTTATTTCAACATTTATTTTGGTTTTTTGGTCATCCAGAAG
TTTACGTAATTATTTTTCCTGCATTTGGCATCATAAGTCAGGTTGTATCAACTTTTTCTCACAGACCTGTATTTGGTTACATAGGGATGGTTTAT
GCAATGATAGGTATAGCAGTATTTGGCTTTATGGTTTGGGCTCATCATATGTTCACTGTTGGGCTTAGTGCTGACGCTGCTGCATTTTTTCTTAT
TTTGAAACTTAATAGTTCCAACTCCTTACATTCGAAGAATCTGACTTCTGATCAAGCAATAACCTCTTCTGTGAAAGATGCACTGCGTTTGGGC
TGCTTGGCTGTTGGATTTACTATATATCCTGGTTCTGCTAAGTGTTTCGATATGATGGAAGAGGCCCGTGAAATCGTAGCTGAAGCCAAATCTT
ATGGGCTTGCAGTAGTGCTATGGTCTTATCCACGCGGTAAAGGAATTTCCAAAGAAGGTGAAACAGCGGTTGATGTTATTGCCTATGCTGCAC
ACATGGCAGCTTTGCTTGGCGCTAATATAATAAAAGTAAAACTTCCAACTAAATATTTGGAAAGGGAGAAAATAGAAAAACCGGTGCTGCAC
CGGTAATTGCAAAAGCAGCCAGAGAAGCAAGAGCTGCAGTTAAGGATAGAGCGCCAAAAGAAAAAAAGATATTGACTGTTGGAGTTGTAAC
TAAACCGTTCGGTTTTGAAGGTGTGCGCCGTATGCGCATTGCAGAGCTTGGACTTGAAGAACTGCAAAAATACGTGGATACACTTATTGTCAT
TCCAAATCAGAATTTATTTAGAATTGCAAATGAAAAAACTACATTTTCTGATGCATTTAAACTTGCTGATAATGTTCTGCATATTGGCATCAGA
GGAGTAACTGACTTGATGGTCATGCCAGGGCTTATTAATCTTGACTTCGCTGATATAGAAACAGTAATGAGCGAGATGGGCAAAGCGATGAT
CGGCACCGGAGAGGCAGAAGGAGAAGATAGAGCAATTAGTGAAGCTGCAGAATGCATGAAAAAATTGAGGCAGATTTTGCGTTACATTGGT
TCGTGTGATGGTGATATGGAAAAGGGATCACTTCGTTGTGATGCAAATGTTTCTGTCCGCCTAAAAGGCAGTAGCACATTTGGCACTCGTTGT
GAGATAAAAAATCTGAACTCGATACGTTATATTGTGCAAGCTATAGACTATGAAATACAAAGACAAATTGAAATTTTAGAAGGTGGGGAAGA
AATAAGTCAAGATACCTTATTGTTTGACGTTGCTTCGGGAAAAACAAAAGTGATGCGAAACAAAGAAGATGCAAGCGACTATAGATACTTCC
CTGAGCCTGATTTATTACCTGTTGAGGTAAGCCAGGAGAAACCGCGCCTTCGCTCTGCTATATTTGCTGCACGCAAGGAAAATCTACCAAAAG
ATAAAAGAGAAACAGCAATAAAAAATGCAACTGGTAACGTTGCTGGAGAAAATTACGAGGAAATACAATATGAAGGTCATGGGCCTTTTGG
CACTGCACTCATTGTCCATGCCTTGACTAATAACCGCAACCGTACTGCTTCTGAGGTGCGTTATATTTTTTCTCGCAAAGGTGGAAATTTAGGA
GAAACAGGAAGCGTTAGTTATCTTTTTGATCATGTAGGTTTAATTGTCTATAAAGCAGAGGGTGTGAATTTTGACGATTTATTCAGTCATGGA
ATCGAATTAGAAGTATTGAATGTTGAGGAAAATGACAAAGAAGGATTACACGTTATAACTTGTGAAATAAAAGATTTTGGTAAAGTACGCGA
TGCCTTT
>Xylosandrus_germanus_wXge1
AAGATGCCACTATTTATTTGGTCTGTCTTGCTAACAGCATTTATGTTGATTGTCGCCTTACCAGTGCTTGCCGGTGCTATAACTATGCTTCTTAC
TGATCGCAATATTGGCACTTCCTTTTTTGATCCTGCAGGTGGTGGTGACCCTGTGTTATTTCAACATTTATTTTGGTTTTTTGGTCATCCAGAAG
TTTACGTAATTATTTTTCCTGCATTTGGCATCATAAGTCAGGTTGTATCAACTTTTTCTCACAGACCTGTATTTGGTTACATAGGGATGGTTTAT
GCAATGATAGGTATAGCAGTATTTGGCTTTATGGTTTGGGCTCATCATATGTTCACTGTTGGGCTTAGTGCTGACGCTGCTGCATTTTTTCTTAT
TTTGAAACTTAATAGTTCCAACTCCTTACATTCGAAGAATCTGACTTCTGATCAAGCAATAACCTCTTCTGTGAAAGATGCACTGCGTTTGGGC
TGCTTGGCTGTTGGATTTACTATATATCCTGGTTCTGCTAAGTGTTTCGATATGATGGAAGAAGCCCGTGAAATCGTAGCTGAAGCCAAATCTT
ATGGGCTTGCAGTAGTGCTATGGTCTTATCCACGCGGTGAAGGAATTTCCAAAGAAGGTGAAACAGCGGTTGATGTTATTGCCTATGCTGCAC
ACATGGCAGCTTTGCTTGGCGCTAATATAATAAAAGTAAAACTTCCAACTAAATATTTGGAAAGGGAGAAAATAGAAAAACCGGTGCTGCAC
CGGTAATTGCAAAAGCAGCCAGAGAAGCAAGAGCTGCAGTTAAGGATAGAGCGCCAAAAGAAAAAAAGATATTGACTGTTGGAGTTGTAAC
TAAACCGTTCGGTTTTGAAGGTGTGCGCCGTATGCGCATTGCAGAGCTTGGACTTGAAGAACTGCAAAAATACGTGGATACACTTATTGTCAT
TCCAAATCAGAATTTATTTAGAATTGCAAATGAAAAAACTACATTTTCTGATGCATTTAAACTTGCTGATAATGTTCTGCATATTGGCATCAGA
GGAGTAACTGACTTGATGGTCATGCCAGGGCTTATTAATCTTGACTTCGCTGATATAGAAACAGTAATGAGCGAGATGGGCAAAGCGATGAT
CGGCACCGGAGAGGCAGAAGGAGAAGATAGAGCAATTAGTGAAGCTGCAGAATGCATGAAAAAATTGAGGCAGATTTTGCGTTACATTGGT
TCGTGTGATGGTGATATGGAAAAGGGATCACTTCGTTGTGATGCAAATGTTTCTGTCCGCCTAAAAGGCAGTAGCACATTTGGCACTCGTTGT
GAGATAAAAAATCTGAACTCGATACGTTATATTGTGCAAGCTATAGACTATGAAATACAAAGACAAATTGAAATTTTAGAAGGTGGGGAAGA
AATAAGTCAAGATACCTTATTGTTTGACGTTGCTTCGGGAAAAACAAAAGTGATGCGAAACAAAGAAGATGCAAGCGACTATAGATACTTCC
CTGAGCCTGATTTATTACCTGTTGAGGTAAGCCAGGAGAAACCGCGCCTTCGCTCTGCTATATTTGCTGCACGCAAGGAAAATCTACCAAAAG
ATAAAAGAGAAACAGCAATAAAAAATGCAACTGGTAACGTTGCTGGAGAAAATTACGAGGAAATACAATATGAAGGTCATGGGCCTTTTGG
CACTGCACTCATTGTCCATGCCTTGACTAATAACCGCAACCATACTGCTTCTGAGGTGCGTTATATTTTTTCTCGCAAAGGTGGAAATTTAGGA
GAAACAGGAAGCGTTAGTTATCTTTTTGATCATGTAGGTTTAATTGTCTATAAAGCAGAGGGTGTGAATTTTGACGATTTATTCAGTCATGGA

ATCGAATTAGAAGTATTGAATGTTGAGGAAAATGACAAAGAAGGATTACACGTTATAACTTGTGAAATAAAAGATTTGGTAAAGTACGCGA
TGCCTTT
>Neptis_rivularis_wNriv1
AAGATACCACTATTTGTTTGGTCTATTTACTCACGTCATTTATGTTAATTGTTGCCTTACCGGTACTTGCTGGTGCTATAACTATGCTGCTCAC
TGATCGTAATATAGGTACCTCCTTTTTTGATCCTGCTGGTGGTGGTGATCCTGTGTTATTTCAACATCTGTTTTGGTTTTTTGGTCATCCAGAAG
TTTACATAATTATTTTTCCTGCATTTGGTATCATCAGTCAGATTGTGTCAACTTTTTCTCATAGGCCAGTATTTGGTTATATGGGAATGGTTTAT
GCCATGATAGGAATAGCAACGTTTGGCTTCATGGTTTGGGCTCACCATATGTTTACTGTTGGGCTTAGCGAGGATGCTGCTATATTTTTCTTA
TTTTGAAGCTTAATAGCGCTAACTCTTTGCACTCAAAAAGCTTAACTTCAGATCAAGCAATAACTGCCTCTGTAAAAGATGCGCTGCGTTTGG
GCTGCATGGCTGTTGGGTTTACTATATATCCTGGTTCTGCTAAGTGTTTTGATATGATGGAAGAAGCTCGCAAAATTATAGCTGAGGCTAAAT
CTTGTGGCCTTGTTGTAGTGCTATGGTCTTATCCACGTGGTGAAGGGGTTTCCAAAGAAGGTGAAACAGCAGTTGATGTGATTGCCTATGCTG
CGCATATAGCGGCTTTGCTCGGTGCCAACATAATAAAAGTAAAACTTCCAACCAATCATCTGGAAAGAGAAAAAATAGAAAAACAGGTGCTG
CACCGGTAATTGCAAAGGCAGCCAGAGAAGCAAGAGCGGTAGTTAAAGATAAGGGAGCAAAAGAAAAAAAGATACTGACTGTTGGAGTTGT
AACTAAGCCGTTCGGTTTTGAAGGTGTGCGACGTATGCGCATTGCAGAGCTTGGACTTGAAGAGTTGCAAAAATACGTAGATACACTTATTGT
CATTCCCAATCAAAATTTATTTAGAATTGCTAACGAGAAAACTACATTTGCTGACGCATTTCAACTCGCCGATAATGTTCTGCATATTGGCATA
AGAGGAGTAACTGATTTGATGATCATGCCAGGACTGATTAATCTTGATTTTGCTGATATAGAAACAGTAATGAGTGAGATGGGTAAAGCAAT
GATTGGTACTGGAGAGGCAGAAGGAGAAGATAGGGCAATTAGTGAAGCTGCAGAATTCATGAAAAAATTGAGGCAGATTTTGCGTTACATC
GGTTCATGTGATGGTGATATGGAAAAGGGGTCACTTCGCTGTGATGCAAATGTTTCTGTTCGCCCAAAGGGTAGTAGCACATTTGGCACTCGT
TGTGAAATAAAAAACTTAAATTCAATACGTTATATTGTACAAGCTATAGATTATGAAGCACAAAGGCAGATCAAAATTTTGGAAAGCGGAGG
AGAAATAAGTCAAGATACCTTATTGTTTGATGTCACTTTAGGAAAAACAAAAGTGATGAGAAGCAAAGAAGATTCAAGTGACTATAGATATT
TCCCTGAACCTGATTTGCTACCTGTTGAAATAAGCCAAGACAAACCACGTCTTCGCTCTGCTATCTTTGCTGCGCGAAAGGAAAATCTACCAA
AAGATAAAATAGAAACAGCAATAAAAAATGCAGCTGGTAACGTTGCTGGAGAAAGTTATGAAGAAATACAATATGAAGGCTGCGGACCTTC
TGGTGCTGCACTTATTGTCCATGCTCTGACAAATAATCGCAACCGAACTGCTTCTGAGATACGTTATATCTTTTCTCGCAAAGGCGGTAATTTG
GGAGAAACAGGATGTGTGAGTTACCTTTTCGATCATGTAGGCTTAATTGTCTATAAAGCAGAGGGTATAAATTTTGAAGATTTATTTAACTAT
GGAATTGAATTAGAAGTATTGAATGTTGAGGAAAATAACAAAGAAGAATTATATGTTATAACTTGTGAAGTAAAAGACTTTGGTAAAGTACG
TGACGCnTTT
>Psyttalia_rhagoleticola_wRha
AAGATGCCACTGTTTGTTTGGTCTGTCTTGCTAACAGCATTTATGTTGATTGTTGCCTTACCAGTGCTTGCCGGTGCTATAACTATGCTTCTTAC
TGATCGCAATGTTGGTACTTCCTTTTTTGATCCTGCAGGTGGTGGTGACCCTGTGTTATTTCAACATTTATTTTGGTTTTTTGGTCATCCAGAAG
TTTACGTAATTATTTTTCCTGCATTTGGCATCATAAGTCAGGTTGTATCAACTTTTTCTCACAGACCTGTATTTGGTTACATAGGGATGGTTTAT
GCAATGATAGGTATAGCAGTATTTGGCTTTATGGTTTGGGCTCATCATATGTTCACTGTTGGGCTTAGTGCTGACGCTGCTGCATTTTTTCTTAT
TTTGAAACTTAATAGTTCCAACTCTTTACATTCAAAGGATCTAACCTCTGATCAGGCAATAACCTCTTCTGTGAAAGATGCGCTGCGTTTGGGC
TGCTTAGCTGTCGGATTTACTATATATCCTGGTTCTGCTAAGTGTTTCGATATGATGGAGGAAGCCCGTGGAATCATAGCTGAAGCCAAATCT
TACGGGCTTGCAGTAGTGCTATGGTCTTATCCACGCGGTGAAGGGATTTCCAAAGAAGGTGAAACAGCAGTTGATGTTATTGCCTATGCTGCA
CACATAGCAGCTTTGCTTGGCGCTAATATAATAAAAGTAAAACTTCCAACTAAATATTTGGAAAGGGAGAAAATAGAAAAACCGGTGCTGCA
CCGGTAATTGCAAAAGCAGCCAGAGAAGCAAGAGCCGCAGTTAAGGATAGAGCGCCAAAAGAAAAAAAGATATTGACTGTTGGAGTTGTAA
CTAAACCGTTCGGTTTTGAAGGTGTGCGCCGTATGCGCATTGCAGAGCTTGGACTTGAAGAACTGCAAAAATACGTGGATACACTTATTGTCA
TTCCAAATCAGAATTTATTTAGAATTGCAAATGAAAAAACTACATTTTCTGATGCATTTAAACTTGCTGACAATGTTCTGCATATTGGCATCAG
AGGGGTAACTGACTTGATGGTCATGCCAGGGCTTATTAATCTTGACTTCGCTGATATAGAAACAGTAATGAGCGAGATGGGCAAAGCGATGA
TTGGCACCGGAGAGGCAGAAGGAGAAGATAGAGCAATTAGTGAAGCTGCAGAATGCATGAAAAGATTAAGGCAGATTTTGCGTTATATTGG
TTCGTGTGATGGTGATATGGAAAAGGGATCACTTCGTTGTGATGCAAATGTTTCTGTTCGCCTAAAAGGCAGTAGTACATTTGGCACTCGTTG
TGAAATAAAAAATCTGAACTCGATACGTTATATTGTGCAAGCTATAGACTATGAAATACAAAGACAAATTGAAATTTTAGAAAGTGGAGAAG
AAATAAGTCAAGATACCTTATTGTTTGATGTTGCTTTGGGAAAAACAAAAGTGATGAGAAGCAAAGAGGATGCAAGCGACTATAGATACTTC
CCTGAGCCTGATTTATTACCTGTTGAGGTAAGCCAGGAGAAACCGCGCCTTCGCTCTGCTATATTTGCTGCACGCAAGGAAAATCTACCAAAA

GATAAAATAGAAACAGCAATAAAAAATGCAACTGGTAACGTTGCTGGAGAAAATTACGAGGAAATACAATATGAAGGTCATGGGCCTTCTG
GCACTGCACTCATTGTCCATGCCTTGACTAATAACCGCAACCGTACTGCTTCTGAGGTACGTTATATCTTTTCTCGTAAAGGTGGAAATTTAGG
AGAAACAGGAAGTGTTAGTTACCTTTTCGATCATGTAGGCTTAATCGTCTATAAAGCAGAGGGTGTGAATTTTGATGATTTATTCAGTCATGG
AATCGAATTAGAAGTATTGAATGTTGAGGAAAATGACAAAGAAGGATTACACGTTATAACTTGTGAAATAAAAGATTTGGTAAAGTACGCG
ATGCCTTT
>Anastrepha_fraterculus_wAfraCast2A
AAGATGCCACTGTTTGTTTGGTCTGTCTTGCTAACAGCATTTATGTTGATTGTTGCCTTACCAGTGCTTGCCGGTGCTATAACTATGCTTCTTAC
TGATCGCAATATTGGTACTTCCTTTTTTGATCCTGCCGGTGGCGGCGATCCTGTGTTATTTCAACATCTATTTTGGTTTTTTGGTCATCCAGAAG
TTTACGTAATTATTTTTCCTGCATTTGGCATCATAAGTCAGGTTGTATCAACTTTTTCTCACAGACCTGTATTTGGTTACATAGGGATGGTTTAT
GCAATGATAGGTATAGCAGTATTTGGCTTTATGGTTTGGGCTCACCATATGTTCACTGTTGGGCTTAGTGCTGACGCTGCTGCATTTTTTCTTA
TTTTGAAACTTAATAGTTCCAACTCTTTACATTCAAAGGATCTAACCTCTGATCAGGCAATAACCTCTTCTGTGAAAGATGCGCTGCGTTTGGG
ATGCTTAGCTGTCGGATTTACTATATATCCTGGTTCTGCTAAGTGTTTCGATATGATGGAGGAAGCCCGTGGAATCATAGCTGAAGCCAAATC
TTATGGACTTGCAGTAGTGCTATGGTCTTATCCACGCGGTGAAGGGATTTCCAAAGAAGGTGAAACAGCAGTTGATGTTATTGCCTATGCTGC
GCACATGGCAGCTTTGCTTGGCGCTAATATAATAAAAGTAAAACTTCCAACTAAATATTTGGAAAGGGAGAAAATAGAAAAACCGGTGCAGC
ACCGGTAATTGCAAAAGCAGCCAGAGAAGCAAGAGCCGCAGTTAAGGATAGAGCGCCAAAAGAAAAAAAGATATTGACTGTTGGAGTTGTA
ACTAAACCGTTCGGTTTTGAAGGTGTGCGCCGTATGCGCATTGCAGAGCTTGGACTTGAAGAACTGCAAAAATACGTGGATACACTTATTGTC
ATTCCAAATCAGAATTTATTTAGAATTGCAAATGAAAAAACTACATTTTCTGATGCATTTAAACTTGCTGATAATGTTCTGCACATTGGCATCA
GAGGAGTAACTGACTTGATGGTCATGCCAGGGCTTATCAATCTTGACTTCGCTGATATAGAAACAGTAATGAGCGAGATGGGCAAAGCGATG
ATCGGCACCGGAGAGGCAGAAGGAGAAGATAGAGCAATTAGTGAAGCTGCAGAATGCATGAAAAAATTGAGGCAGATTTTGCGTTACATTG
GTTCGTGTGATGGTGATATGGAAAAGGGATCACTTCGTTGTGATGCAAATGTTTCTGTCCGCCTAAAAGGCAGTAGTACATTTGGCACTCGTT
GTGAAATAAAAAATCTGAACTCGATACGTTATATTGTGCAAGCTATAGACTATGAAATACAAAGACAAATTGAAATTTTAGAAAGTGGAGAA
GAAATAAGTCAAGATACCTTATTGTTTGACGTTGCTTCGGGAAAAACAAAAGTGATGAGAAGCAAAGAGGATGCAAGCGATTATAGATACTT
CCCTGAGCCTGATTTATTACCTGTTGAGGTAAGCCAGGATAAACCGCGCCTTCGCTCTGCTATATTTGCTGCACGCAAGGAAAATCTACCAAA
AGATAAAATAGAAACAGCAATAAAAAATGCAACTGGTAACGTTGCTGGAGAAAATTACGAGGAAATCCAATATGAAGGTCATGGGCCTTCT
GGCACTGCACTCATTGTCCATGTTTGACTAATAATCGCAACCGAACTGCTTCTGAGGTACGTTATATATTTTCTCGCAAGGGTGGAAACTTGG
GAGAAACAGGAAGTGTTAGTTACCTTTTCGATCATGTAGGCTTAATCGTCTATAAAGCAGAGGGTGTGAATTTTGATGATTTATTCAGTCATG
GAATCGAATTAGAAGTATTGAATGTTGAGGAAAATGACAAAGAAGGATTACACGTTATAACTTGTGAAATAAAAGATTTGGTAAAGTACGC
GATGCCTTT
>Aedes_albopictus_wAlbB
AAGATGCCACTGTTTGTTTGGTCTGTCTTGTTAACAGCATTTATGCTGATTGTTGCCTTACCAGTGCTTGCCGGTGCTATAACTATGCTTCTTAC
TGATCGCAATATTGGTACTTCCTTTTTTGATCCTGCTGGTGGTGGTGATCCTGTGTTATTTCAACATCTGTTTTGGTTTTTTGGTCATCCGGAGG
TTTACGTAATTATTTTTCCTGCATTTGGCATCATAAGTCAGGTTGTATCAACTTTTTCTCACAGACCTGTATTTGGTTACATAGGAATGGTTTAT
GCAATGATAGGTATAGCAGTATTTGGCTTTATGGTTTGGGCTCACCATATGTTCACTGTTGGGCTTAATGCTGACGCTGCTGCATTTTTTCTTA
TTTTGAAGCTTAATAGCGCTAACTCTTTGCACTCAAAAAGCTTAACTTCAGATCAAGCAATAACTGCCTCT-----
AGATGCGCTGCGTTTGGGCTGCATGGCTGTTGGGTTTACTATATATCCTGGTTCTGCTAAGTGTTTTGATATGATGGAAGAAGCTCGCAAAATT
ATAGCTGAGGCTAAATCTTGTGGCCTTGCTGTAGTGCTATGGTCTTATCCACGTGGTGAAGGGATTTCCAAAGAAGGTGAAACAGCAGTTGAT
GTGATTGCTTATGCTGCGCATATAGCGGCTTTGCTCGGTGCCAACATAATAAAAGTAAAACTTCCAACCAATCATCTGGAAAGAGAAAAAAT
AGAAAAACAGGCGCTGCACCGGTAATTGCAAAAGCAGCAAGAGAAGCAAGAGCGGTAGTTAAAGATAAAGGAGCAAAAGAAAAAAAGATA
CTGACTGTTGGAGTTGTAACTAAGCCGTTCGGTTTTGAAGGTGTGCGACGTATGCGCATTGCAGAGCTTGGACTTGAAGAGTTGCAAAAATAC
GTAGATACACTTATTGTCATTCCTAATCAAAATTTATTTAGAATTGCTAACGAGAAAACTACATTTGCTGACGCATTTCAACTCGCCGATAATG
TTCTGCATATTGGCATAAGAGGAGTAACTGATTTGATGATCATGCCAGGACTGATTAATCTTGATTTTGCTGATATAGAAACAGTAATGAGTG
AGATGGGTAAAGCAATGATTGGTACTGGAGAGGCAGAGGGAGAAGATAGGGCAATTAGTGAAGCTGCAGAATTCGTGAAAAAATTGAGGCA
GATTTTGCGTTACATCGGTTCATGTGATGGTGATATGGAAAAGGGGTCACTTCGCTGTGATGCAAATGTTTCTGTTCGCCCAAAGGGTAGTAG

CACATTTGGCACTCGTTATGAAATAAAAAACTTAAATTCAATACGTTATATTGTACAAGCTATAGATTATGAAGCACAAAGGCAGATCAAAA
TTTTGGAAAGCGGAGGAGAAATAAATCAAGATACCTTATTGTTTGATGCCACTTTAGGAAAAACAAAAGTGATGAGAAGCAAAGAAGATTCA
AGTGACTATAGATATTTCCCTGAACCTGATTTACTACCTGTTGAAATAAGCCAAGACAAACCACGTCTTCGCTCTGCTATATTTGCTGCGCGA
AAGGAAAATCTACCAAAAGATAAAATAGAAACAGCAATAAAAAATGCAGCTGGTAACGTTGCTGGAGAAAGTTATGAAGAAATACAATATG
AAGGCTGCGGACCTTTTGGTGCTGCACTTATTGTCCATGCTCTGACAAATAATCGCAACCGAACTGCTTCTGAGATACGTTATATCTTTTCTCG
CAAAGGCGGTAATTTGGGAGAAACAGGATGTGTGAGTTATCTTTTCGATCATGTAGGCTTAATTGTCTATAAAGCAGAGGGTATAAATTTTGA
AGATTTATTTAACTATGGAATTGCATTAGAAGTATTGAATGTTGAGGAAAATAACAAAGAAGAATTATATGTTATAACTTGTGAAGTAAAAG
ACTTTGGTAAAGTACGTGACGCTTTC
>Drosophila_simulans_wMA
AAAATGCCACTGTTTGTTTGGTCTGTTCTATTAACATCGTTTATGTTAATTGTAGCTTTACCGGTACTTGCTGGTGCTATAACTATGCTGCTCAC
TGATCGCAATATTGGCACTTCCTTTTTTGATCCTGCTGGTGGTGGTGATCCTGTGTTATTTCAACATCTGTTTTGGTTTTTTGGTCACCCAGAGG
TTTATATCATTATTTTTCCTGCGTTTGGCATTATAAGCCAAGTCGTGTCAACTTTTTCCCATAGACCAGTATTTGGCTATAAGGGAATGGTTTAT
GCAATGATAGGTATAGCAGCATTTGGTTTTATGGTTTGGGCTCACCATATGTTTACTGTTGGGCTTAGCGAAGATGCTGCTGTGTTTTTTCTTA
TTTTGAAGCTTAATAGCGCTAACTCTTTGCACTCAAAAAGCTTAACTTCAGATCAAGCAATAACTTCCTCTGTAAAAGATGCTCTACGTTTGGG
CTGCGTGGCTGTTGGGTTTACTATATATCCTGGTTCTGCTAAGTGTTTTGATATGATGGAAGAAGCTCGCAAAATTATAGCTGAGGCTAAATCT
TGTGGCCTTGCTGTAGTGCTATGGTCTTATCCACGTGGTGAAGGGATTTCAAAAGAAGGTGAAACAGCAGTTGATGTCATTGCTTATGCTGCG
CATATAGCGGCTTTGCTTGGTGCCAACATAATAAAAGTAAAACTTCCAACTAACCACTTGGAAAAAGAAAAAATAGAAAAACAGGCGCTGCA
CCGGTAATTGCAAAAGCAGCCAGAGAAGCAAGAGCGGTAGTTAAAGATAAAGGAGCAAAAGAAAAAAAGATACTGACTGTTGGAGTTGTAA
CTAAGCCGTTCGGTTTTGAAGGTGTGCGACGTATGCGCATTGCAGAGCTTGGACTTGAAGAGTTGCAAAAATACGTAGATACACTTATTGTCA
TTCCTAATCAAAATTTATTTAGAATTGCTAACGAGAAAACTACATTTGCTGACGCATTTCAACTCGCCGATAATGTTCTGCATATTGGCATAAG
AGGAGTAACTGATTTGATGATCATGCCAGGACTGATTAATCTTGATTTTGCTGATATAGAAACAGTAATGAGTGAGATGGGTAAAGCAATGA
TTGGTACTGGAGAGGCAGAAGGAGAAGATAGGGCAATTAGTGAAGCTGCAGAATTCATGAAAAAATTGAGGCAGATTTTGCGTTACATCGGT
TCATGTGATGGTGATATGGAAAAGGGGTCACTTCGCTGTGATGCAAATGTTTCTGTTCGCCCAAAGGGTAGTAGCACATTTGGCACTCGTTGT
GAAATAAAAAACTTAAATTCAATACGCTATATTGTACAAGCTATAGATTATGAAGCACAAAGGCAGATCAAAATTTTGGAAAGCGGAGGAG
AAATAAGTCAAGATACCTTATTGTTTGATGTCACTTTAGGAAAAACAAAAGTGATGAGAAGCAAAGAAGATTCAAGTGACTATAGATATTTC
CCTGAACCTGATTTGCTACCTGTTGAAATAAGCCAAGACAAACCACGTCTTCGCTCTGCTATATTTGCTGCGCGAAAGGAAAATCTACCAAAA
GATAAAATAGAAACAGCAATAAAAAATGCAGCTGGTAACGTTGCTGGAGAAAGTTATGAAGAAATACAATATGAAGGCTGCGGACCTTTTG
GTGCTGCACTTATTGTCCATGCTCTGACAAATAATCGCAACCGAACTGCTTCTGAGATACGTTATATCTTTTCTCGCAAAGGCGGTAATTTGGG
AGAAACAGGATGTGTGAGTTATCTTTTCGATCATGTAGGCTTAATTGTCTATAAAGCAGAGGGTATAAATTTTGAAGATTTATTTAACTATGG
AATTGCATTAGAAGTATTGAATGTTGAGGAAAATAACAAAGAAGAATTATATGTTATAACTTGTGAAGTAAAAGACTTTGGTAAAGTACGTG
ACGCTTTC
>Erebia_jeniseiensis_Ejen
AAGATGCCACTGTTTGTTTGGTCTGTCTTGTTAACAGCATTTATGTTGATTGTTGCCTTACCAGTGCTTGCCGGTGCTATAACTATGCTTCTTAC
TGATCGCAATATTGGTACTTCCTTTTTTGATCCTGCCGGTGGTGGTGACCCTGTGTTATTTCAACATTTATTTTGGTTTTTTGGTCATCCGGAGG
TTTACGTAATTATTTTTCCTGCATTTGGCATCATAAGTCAGGTTGTATCAACTTTTTCTCACAGACCTGTATTTGGTTACATAGGAATGGTTTAT
GCAATGATAGGTATAGCAGTATTTGGCTTTATGGTTTGGGCTCACCATATGTTCACTGTTGGGCTTAGTGCTGACGCTGCTGCATTTTTTCTTA
TTTTGAAGCTTAATAGCGCTAACTCTTTGCACTCAAAAAGCTTAACTTCAGATCAAGCAATAACTGCCTCTGTAAAAGATGCGCTGCGTTTGG
GCTGCATGGCTGTTGGGTTTACTATATATCCTGGTTCTGCTAAGTGTTTTGATATGATGGAAGAAGCTCGCAAAATTATAGCTGAGGCTAAAT
CTTGTGGCCTTGCTGTAGTGCTATGGTCTTATCCACGTGGTGAAGGGATTTCCAAAGAAGGTGAAACAGCAGTTGATGTGATTGCTTATGCTG
CGCATATAGCGGCTTTGCTCGGTGCCAACATAATAAAAGTAAAACTTCCAACCAATCATCTGGAAAGAGAAAAAATAGAAAAACAGGTGCTG
CACCGGTAATTGCAAAAGCAGCAAGAGAAGCAAGAGCGGTAGTTAAAGATAAAGGAGCAAAAGAAAAAAAGATACTGACTGTTGGAGTTGT
AACTAAGCCGTTCGGTTTTGAAGGTGTGCGACGTATGCGCATTGCAGAGCTTGGACTTGAAGAGTTGCAAAAATACGTAGATACACTTATTGT
CATTCCCAATCAAAATTTATTTAGAATTGCTAACGAGAAAACTACATTTGCTGACGCATTTCAACTCGCCGATAATGTTCTGCATATTGGCATA

AGAGGAGTAACTGATTTGATGATCATGCCAGGACTGATTAATCTTGATTTTGCTGATATAGAAACAGTAATGAGTGAGATGGGTAAAGCAAT
GATTGGTACTGGAGAGGCAGAAGGAGAAGATAGGGCAATTAGTGAAGCTGCAGAATTCATGAAAAAATTGAGGCAGATTTTGCGTTACATC
GGTTCATGTGATGGTGATATGGAAAAGGGGTCACTTCGCTGTGATGCAAATGTTTCTGTTCGCCCAAAGGGCAGTAGCACATTTGGCACTCGT
TGTGAAATAAAAAACTTAAATTCAATACGTTATATTGTACAAGCTATAGATTATGAAGCACAAAGGCAGATCAAAATTTTGGAAAGCGGAGG
AGAAATAAGTCAAGATACCTTATTGTTTGATGTCACTTTAGGAAAAACAAAAGTGATGAGAAGCAAAGAAGATTCAAGTGACTATAGATATT
TCCCTGAACCTGATTTGCTACCTGTTGAAATAAGCCAAGACAAACCACGTCTTCGCTCTGCTATCTTTGCTGCGCGAAAGGAAAATCTACCAA
AAGATAAAATAGAAACAGCAATAAAAAATGCAGCTGGTAACGTTGCTGGAGAAAGTTATGAAGAAATACAATATGAAGGCTGCGGACCTTC
TGGTGCTGCACTTATTGTCCATGCTCTGACAAATAATCGCAACCGAACTGCTTCTGAGATACGTTATATCTTTTCTCGCAAAGGCGGTAATTTG
GGAGAAACAGGATGTGTGAGTTACCTTTTCGATCATGTAGGCTTAATTGTCTATAAAGCAGAGGGTATAAATTTTGAAGATTTATTTAACTAT
GGAATTGAATTAGAAGTATTGAATGTTGAGGAAAATAACAAAGAAGAATTATATGTTATAACTTGTGAAGTAAAAGACTTTGGTAAAGTACG
TGACGCTTTC
>A_PS122
AAGATGCCACTGTTTGTTTGGTCTGTCTTGCTAACAGCATTTATGTTGATTGTTGCCTTACCGGTGCTTGCCGGTGCTATAACTATGCTTCTTAC
TGATCGCAATATTGGTACTTCCTTTTTTGATCCTGCCGGTGGTGGTGACCCTGTGTTATTTCAACATTTATTTTGGTTTTTTGGTCATCCAGAAG
TTTACGTAATTATTTTTCCTGCATTTGGCATCATAAGTCAGGTTGTATCAACTTTTTCTCACAGACCTGTATTTGGTTACATAGGGATGGTTTAT
GCAATGATAGGTATAGCAGTATTTGGCTTTATGGTTTGGGCTCATCATATGTTCACTGTTGGGCTTAGTGCTGACGCTGCTGCATTTTTTCTTAT
TTTGAAACTTAATAGTTCCAACTCCTTACATTCGAAGAATCTGACTTCTGATCAAGCAATAACCTCTTCTGTGAAAGATGCACTGCGTTTGGGC
TGCTTGGCTGTTGGATTTACTATATATCCTGGTTCTGCTAAGTGTTTCGATATGATGGAAGAAGCACGTAAAATCGTAGCTGAAGCCAAATCT
TATGGGCTTGCAGTAGTGTTATGGTCTTATCCACGCGGTGAAGGAATTTCCAAAGAAGGTGAAACAGCGGTTGATGTTATTGCCTATGCTGCA
CACATGGCAGCTTTGCTTGGCGCTAATATAATAAAAGTAAAACTTCCAACTAAATATTTGGAAAGGGAGAAAATAGAAAAACCGGTGCAGCA
CCGGTAATTGCAAAAGCAGCCAGAGAAGCAAGAGCCGCAGTTAAGGATAGAGCGCCAAAAGAAAAAAAGATATTGACTGTTGGAGTTGTAA
CTAAACCGTTCGGTTTTGAAGGTGTGCGCCGTATGCGCATTGCAGAGCTTGGACTTGAAGAACTGCAAAAATACGTGGATACACTTATTGTCA
TTCCAAATCAGAATTTATTTAGAATTGCAAATGAAAAAACTACATTTTCTGATGCATTTAAACTTGCTGATAATGTTCTGCACATTGGCATCAG
AGGAGTAACTGACTTGATGGTCATGCCAGGGCTTATCAATCTTGACTTCGCTGATATAGAAACAGTAATGAGCGAGATGGGCAAAGCGATGA
TCGGCACCGGAGAGGCAGAAGGAGAAGATAGAGCAATTAGCGAAGCTGCAGAATGCATGAAAAAATTGAGGCAGATTTTGCGTTACATTGG
TTCGTGTGATGGTGATATGGAAAAGGGATCACTTCGTTGTGATGCAAATGTTTCTGTCCGCCTAAAAGGCAGTAGCGCACTTGGCACTCGTTG
TGAGATAAAAAATCTGAACTCGATACGTTATATTGTGCAAGCTATAGACTATGAAATACAAAGACAAATTGAAATTTTAGAAAGTGGGGAAG
AAATAAGTCAAGATACCTTATTGTTTGATGTTGCTTCGGGAAAAACAAAAGTGATGAGAAGCAAAGAGGATGCAAGCGACTATAGATACTTC
CCTGAGCCTGATTTATTACCTGTTGAGGTAAGCCAGGAGAAACCGCGCCTTCGCTCTGCTATATTTGCTGCACGCAAGGAAAATCTACCAAAA
GATAAAATAGAAACAGCAATAAAAAATGCAACTGGTAACGTTGCTGGAGAAAATTACGAGGAAATACAATATGAAGGTCATGGGCCTTCTG
GCACTGCACTCATTGTCCATGCCTTGACTAATAACCGCAACCGTACTGCTTCTGAGGTACGTTATATCTTTTCTCGTAAAGGTGGAAATTTAGG
AGAAACAGGAAGTGTTAGTTACCTTTTCGATCATGTAGGCTTAATCGTCTATAAAGCAGAGGGTGTGAATTTTGATGATTTATTCAGTCATGG
AATCGAATTAGAAGTATTGAATGTTGAGGAAAATGACAAAGAAGGATTACACGTTATAACTTGTGAAATAAAAGATTTTGGTAAAGTACGCG
ATGCCTTT

Supplementary File 4. Gene partitions with estimated nucleotide substitution models used in the Bayesian inference (BI) analysis based on a comparison of scores from Akaike Information Criterion (AIC) and Bayesian Information Criterion (BIC) in JModeltest.

Gene partitions	Substitution models
Wsp trees:	
wsp = 1-474 (Figure S2A)	GTR + gamma
wsp = 1-477 (Figure S3A)	GTR + I + gamma
MLST trees (Figure S2A):	
coxA = 1-384	GTR + gamma
fbpA = 385-748	GTR + gamma
ftsZ = 749-1172	GTR + gamma
gatB = 1173-1541	GTR + I + gamma
hcpA = 1542-1970	GTR + I
